# Plasma proteomic profiles of Alzheimer’s disease and neurodegeneration in African cohorts

**DOI:** 10.1038/s41467-026-74971-4

**Published:** 2026-07-01

**Authors:** Ilaria Pola, Tolulope Akinyemi, Kubra Tan, Oladotun Olalusi, Wiebke Traichel, Giulia Giacomucci, Joseph Yaria, Nesrine Rahmouni, Gabriel Ogunde, Olabode Oguntiloye, Arthur C. Macedo, Ayotomiwa Fagbemi, Eniola Cadmus, Joseph Therriault, Femi Popoola, Stella-Maria Paddick, Catherine Dotchin, Alyoce Kisoli, Richard Walker, Mayowa Ogunronbi, Dorcas Olujobi, Olaoluwa Famuyiwa, Joshua Akinyemi, Mayowa Owolabi, Chinedu T. Udeh–Momoh, Olusola Ladokun, Anthony Griswold, Roman Romero-Ortuno, Margaret Pericak-Vance, Adesola Ogunniyi, Stephanie Debette, Brian Lawlor, Nicholas J. Ashton, Rajesh Kalaria, Henrik Zetterberg, Pedro Rosa-Neto, Rufus O. Akinyemi, Andrea L. Benedet

**Affiliations:** 1 Department of Psychiatry and Neurochemistry, Institute of Neuroscience and Physiology, Sahlgrenska Academy, University of Gothenburg, Mölndal, Sweden; 2 Department of Biological Sciences, Faculty of Natural and Applied Science, Lead City University, Ibadan, Nigeria; 3 Department of Biomedical Laboratory Science, Faculty of Basic Medical Sciences, College of Medicine, University of Ibadan, Ibadan, Nigeria; 4 Neuroscience and Ageing Research Unit, Institute for Advanced Medical Research and Training, College of Medicine, University of Ibadan, Ibadan, Nigeria; 5 Department of Neurology, University College Hospital, Ibadan, Oyo, Nigeria; 6 Department of Neuroscience, Psychology, Drug Research and Child Health, University of Florence, Florence, Italy; 7 McGill Centre for Studies in Aging, Douglas Hospital Research Centre (CIUSSS de l’Ouest-de-l’Île-de-Montréal), Verdun, Quebec, MC Canada; 8 Department of Neurology and Neurosurgery, Faculty of Medicine, McGill University, Montreal, Quebec, MC Canada; 9 Department of Community Medicine, Faculty of Public Health, College of Medicine, University of Ibadan, Ibadan, Nigeria; 10 Translational and Clinical Research Institute, Biomedical Research Centre, Newcastle University, Campus for Ageing & Vitality, Newcastle upon Tyne, UK; 11 Gateshead Health NHS Foundation Trust, Gateshead, UK; 12 Millennium Institute for Care Research (MiCARES), University Andres Bello, Santiago, Chile; 13 AGE Research Group, Translational and Clinical Research Institute, Faculty of Medical Sciences, Newcastle University, Newcastle upon Tyne, UK; 14 NIHR Newcastle Biomedical Research Centre, Newcastle upon Tyne, UK; 15 Newcastle upon Tyne Hospitals NHS Foundation Trust, Newcastle upon Tyne, UK; 16 Cumbria, Northumberland, Tyne and Wear NHS Foundation Trust, Newcastle upon Tyne, UK; 17 Northumbria Healthcare NHS Foundation Trust, North Shields, Tyne and Wear, Newcastle upon Tyne, UK; 18 Kilimanjaro Clinical Research Institute, Kilimanjaro Christian Medical College, Moshi, Tanzania; 19 Population Health Sciences Institute, Newcastle University, Newcastle upon Tyne, UK; 20 Department of Epidemiology and Medical Statistics, College of Medicine, University of Ibadan, Ibadan, Nigeria; 21 Centre for Genomic and Precision Medicine, College of Medicine, University of Ibadan, Ibadan, Nigeria; 22 Division of Public Health Sciences, Wake Forest University School of Medicine, Winston-, Salem, NC USA; 23 Global Brain Health Institute, Trinity College Dublin, Dublin, Ireland; 24 Brain and Mind Institute, Aga Khan University, Nairobi, Kenya; 25 John P. Hussman Institute for Human Genomics, Miller School of Medicine, University of Miami, Miami, FL USA; 26 Discipline of Medical Gerontology, School of Medicine, Trinity College Dublin, Dublin, Ireland; 27 Bordeaux Population Health Center, INSERM UMR U1219, University of Bordeaux, Bordeaux, France; 28 Department of Neurology, Institute for Neurodegenerative Diseases, CHU de Bordeaux, Bordeaux, France; 29 Institut du Cerveau (ICM), Paris Brain Institute, INSERM U1127, CNRS UMR 7225, Sorbonne Université, AP-HP, Paris, France; 30 Trinity College Institute of Neuroscience, School of Psychology, Trinity College Dublin, Dublin, Ireland; 31 Banner Alzheimer’s Institute, Phoenix, AZ USA; 32 Banner Sun Health Research Institute, Sun City, AZ USA; 33 Clinical Neurochemistry Laboratory, Sahlgrenska University Hospital, Mölndal, Sweden; 34 Department of Neurodegenerative Disease, UCL Institute of Neurology, Queen Square, London, UK; 35 UK Dementia Research Institute at UCL, London, UK; 36 Wisconsin Alzheimer’s Disease Research Center, University of Wisconsin School of Medicine and Public Health, Madison, WI USA; 37 Department of Pathology and Laboratory Medicine, University of Wisconsin School of Medicine and Public Health, Madison, WI USA; 38 Peter O’Donnell Jr. Brain Institute, University of Texas Southwestern Medical Center, Dallas, TX USA

**Keywords:** Diagnostic markers, Alzheimer's disease

## Abstract

Alzheimer’s disease and related dementia (ADRD) represents a growing public health burden, especially in low- and middle-income countries. Yet, most studies focus on Non-Hispanic white (NHW) populations from high-income countries. This study investigates plasma proteomic signatures associated with amyloid pathology in African populations. Nigerian older adults from the VALIANT study and participants from a Tanzanian study, with available biomarker quantification in plasma are employed. For proteomic comparison, participants from the Canadian TRIAD cohort are included, capturing a distinct population. Here, we show that multiple proteins are differentially abundant in the plasma of p-tau217-positive individuals and in the different cognitive groups, with findings largely consistent amongst African cohorts. Comorbidities are significantly associated with protein levels and differences in plasma biomarkers levels are found between sexes. Lastly, VALIANT and TRIAD show both shared and unique protein profiles in relation to amyloid-pathology. These findings support the utility of fluid biomarkers in ADRD in African populations.

## Abstract (web summary)

This study examines the relationship between plasma proteomic signatures and amyloid pathology in African individuals to advance understanding of blood-based biomarkers for Alzheimer’s disease and improve their applicability across diverse populations.

## Introduction

Alzheimer’s disease and related dementias (ADRD) represent a considerable burden on society, particularly in the elderly population^1^. With the increase of the aging population in the last decade, the number of individuals affected by dementia globally is expected to reach 74.7 million in 2030 and increase to 132 million in 2050^2^. This scenario is all the more complex in the African context, where the number of individuals with Alzheimer’s disease (AD) is projected to increase far more disproportionately in the future^3,4^. Such scenario can be explained by multiple factors, such as the higher growth rate of the aging population in Africa in combination with challenges related to healthcare infrastructure, socioeconomic inequality, and environmental stressors as also described in other low- and middle-income countries (LMIC)^5,6^. To address these challenges, this study explores plasma proteomic signatures in relation to amyloid pathology in African individuals.

The AD research in the African context has been primarily focused on genetic studies. Indeed, genome wide association studies (GWAS) have reported stronger associations of *ABCA7* gene with AD risk in individuals with African American ancestry rather than in individuals with European ancestry^7^. Furthermore, previous research has shown that the influence of *APOE*-ε4 on AD is attenuated in African populations, compared to non-Hispanic white (NHW) populations^8^. Such difference has been reported to lie in the differential ancestral background surrounding the *APOE* locus, altering how the gene is expressed and its functions^8^.

While GWAS studies have provided crucial insights, plasma proteomic studies and blood-based biomarkers are essential for the diagnostic aspect, as they offer a non-invasive, scalable and low-resource alternative^9^. Major proteomic studies have been performed among NHW populations^10,11^, and though multi-ancestry studies of biomarkers have been recently undertaken^12^, other ethnic groups have been underrepresented. To date, biofluid biomarker studies in African populations have been limited. Recent studies in a Congolese cohort^13–15^ have focused on core AD markers (inlcuding GFAP, NfL and p-tau species) and their associations with cognitive performance and dementia, providing reference values for plasma AD biomarkers in the congolese settings. Additionally, and a narrow set of inflammatory markers were also explored. Furthermore, studies in indigenous Nigerian cohorts have expanded such findings by evaluating plasma Aβ42/40, p-tau217, GFAP and NfL and their associations with cognition and dementia^16^. However, existing studies are often restricted to small cohorts, limiting their statistical robustness and generalizability. While emerging studies in African-ancestry cohorts are beginning to capture ancestry-specific biology, addressing a critical gap at both the proteomic and metabolomic levels^17,18^, biomarker research in these populations remain  scarse.

Proteomic studies provide novel insights into proteins linked to pathology and can uncover biomarker signatures for diagnosis, disease staging and monitoring^11^. In African populations, such studies are particularly valuable given the continent’s high genetic diversity, which may yield unique proteomic signatures and test the generalizability of current biomarker panels. Moreover, the high prevalence of vascular, metabolic, and infectious comorbidities—including diabetes, hypercholesterolemia, HIV and malaria—adds complexity to the biological landscape of dementia^19–22^. Proteomic data obtained in African cohorts addresses the important need of understanding population variances in biomarkers; it is essential for testing biomarker generalizability, discovering novel biology shaped by genetics, lifestyle, and environmental interactions, while disentangling the influence of comorbidities, and developing affordable, locally relevant diagnostic and prevention strategies. Well-designed proteomic programs, integrated with genomics and clinical data and grounded in capacity building, have the potential to generate insights that advance both African and global dementia research.

This study aimed to evaluate the plasma concentrations of several proteins related to central nervous system in a cohort of community-dwelling older Nigerian Africans and explore their expression in relation to cognitive status, amyloid pathology and comorbidities. The primary analyses were further replicated in an independent indigenous African cohort from northern Tanzania. To disentangle population-specific risks and underlying pathological mechanisms, findings were then compared with those of participants from the Translational Biomarkers in Aging and Dementia (TRIAD) cohort, a deeply phenotyped Canadian longitudinal study with available NULISA data on plasma. This cohort served as a comparator, representing populations from high-income settings in which most biomarker research has been conducted so far, and providing a contrast to the Nigerian cohort.

## Results

### Participants and biomarker characteristics

**Fig. 1:**
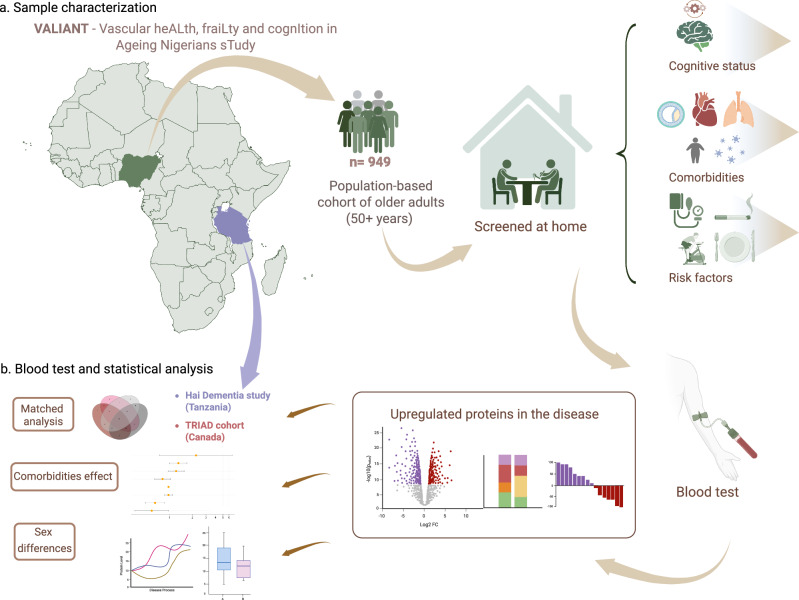
Graphical abstract. **a** Participants from VALIANT cohort (*n* = 949), in Nigeria, were recruited as a population-based cohort of older adults ( > 50 years of age). The participants enrolled in the study were screened in their homes, and assessed for cognitive status, comorbidities and risk factors. For replication, participants from a study conducted in Tanzania (*n* = 196) and with available NULISA data were included. **b** Blood samples were collected, and protein levels were measured with the NULISA (Alamar^TM^) methodology. Statistical analysis (volcano plots, stacked bar charts, gene ontology, linear models, local weighted regression method) were performed to evaluate the relevance of these proteins in AD in the African context. Additionally, participants from the Canadian TRIAD cohort, representing a distinct population context were included for proteomic comparison. Schematic created in BioRender. Pola, I. (2026). https://BioRender.com/3rgxqsp. n = Number; TRIAD = Translational Biomarkers in Aging and Dementia; VALIANT= Vascular health, frailty and cognition in Ageing Nigerians study.

Overall, 1031 participants were enrolled in the Vascular heAlth, fraiLty, and cognItion in Ageing Nigerians sTudy (VALIANT) study, of which 949 had available blood-based biomarkers and sociodemographic data. The average age of this sample was 65.1 (SD ± 10.4) years, 26.6% were male, the average years of formal education was 5.7 (SD ± 4.8) and 14.5% had cognitive impairment, as mild cognitive impairment (MCI) or dementia (Table 1). Participants also had available information on comorbidities, such as cardiovascular disorders (44.3%) and history of infectious diseases (68.2%), predominantly malaria (66.0%). As further described in the methods, the likely presence of amyloid pathology is here proxied by p-tau217 positivity as measured on the Single Molecule Array (Simoa) platform and, from now on, participants will be referred to as amyloid-positive or amyloid-negative accordingly, to facilitate the narrative. To replicate the main analysis, 196 participants from the Tanzanian study were included, of which 196 had available blood-based biomarker data together with age, sex and sociodemographic data; 131 participants had available information on education level (66.5%). The average age of this cohort was 80.3 (SD ± 10.4) years, 34.1% were male, and 61.1% and 40.8% had cognitive impairment as mild cognitive impairment (MCI) or dementia, respectively (Supplementary Table 1). For a comparative analysis, we utilized matched data from the TRIAD cohort from Canada. Match 1 included 315 participants, matched by age, sex and clinical status (cognitively unimpaired (CU), MCI, Dementia); Match 2 included 376 participants matched by age, sex and plasma p-tau217 levels (Simoa); and Match 3 included 290 participants matched by age, sex and clinicopathological groups, this being a combination of clinical status and amyloid pathology groups–here proxied by Simoa plasma p-tau217 status. The rationale for these matching strategies was to assess whether differences in proteomic profiles would remain consistent across amyloid-related disease burden as well as clinical stages between cohorts. For Match 1 mean age was 70.0 (SD ± 8.9) years, 40.6% were male, individuals had on average 15.3 (SD ± 3.7) years of education and 39.1% had cognitive impairment. For Match 2 mean age was 68.9 (SD ± 9.1) years, 38.5% were male, individuals had on average 15.1 (SD ± 3.7) years of education and 48.9% had cognitive impairment. For Match 3 mean age was 69.8 (SD ± 9.2) years, 43.1% were male, individuals had on average 15.3 (SD ± 3.6) years of education and 33.8% had cognitive impairment. The demographic and biomarker information for the whole cohort, as well as for the matched data (Matches 1–3) are presented in Supplementary Tables 2 and 3. Figure 1 displays an overview of the study design and analyses performed.

**Table 1:** Demographics of the participants by clinical group

	CU (N = 811)	MCI (N = 106)	Dem (N = 32)	p-value
Sex				0.03
Male	227 (28%)	17 (16%)	8 (25%)	
Female	581 (72%)	89 (84%)	24 (75%)	
Age	64 (10)	73 (10)	77 (11)	1.10e-22
Years of education	6.2 (4.8)	2.5 (3.7)	1.9 (3.7)	6.74e-18
Aß status				4.35e- 3
Negative	692 (85%)	84 (79%)	21 (66%)	
Positive	119 (15%)	22 (21%)	11 (34%)	

Continuous variables were compared across diagnostic groups using the Kruskal-Wallis rank-sum test. Categorical variables were compared using Pearson’s chi-squared test. Both tests were performed as two-sided omnibus tests. Exact P values are reported where applicable. N(CU) = 811, N(MCI) = 106, N(Dem)= 32.

*Aß* amyloid, *CU* cognitively unimpaired, *Dem* dementia, *MCI* mild cognitively impaired, *N* number.

### Proteomic profiles related to amyloid pathology status

**Fig. 2:**
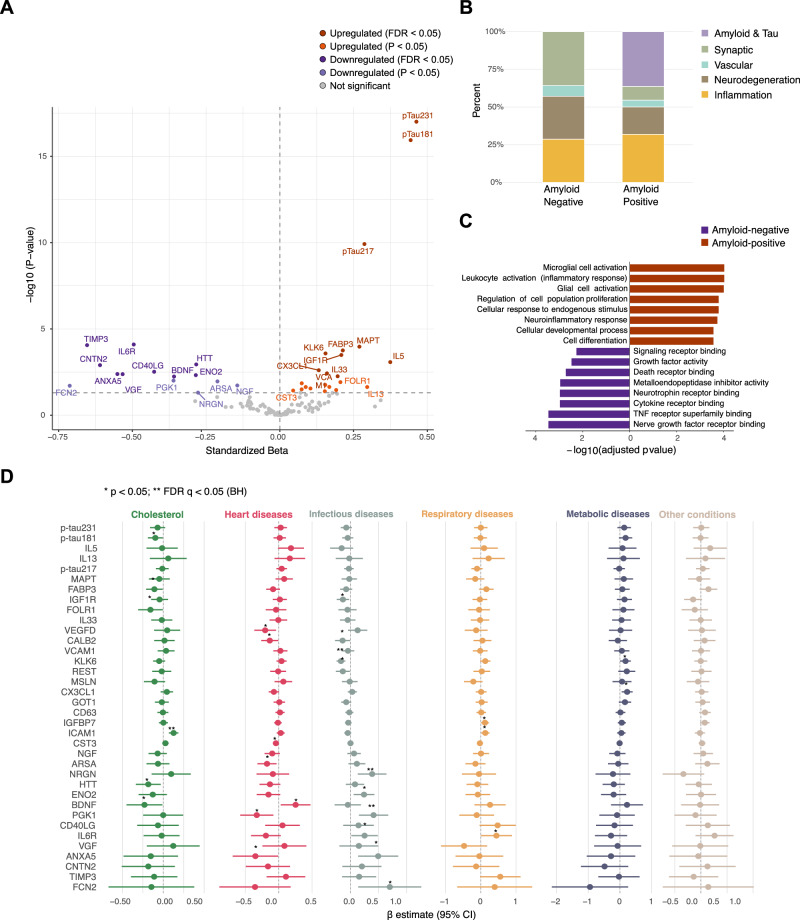
Characterization of proteomic profiles related to amyloid pathology status. **A** The volcano plot displays the standardized beta values of the protein associations in plasma (x-axis) against statistical significance −log10(P value) (y-axis) between amyloid-positive (*N* = 152) and amyloid-negative (*N* = 797) individuals. Models were adjusted for age, sex and average of the proteins. P values were derived from two-sided tests of the regression coefficient. Multiple comparisons were adjusted using the Benjamini–Hochberg (BH) false discovery rate (FDR) method. Significant proteins are colored according to direction of association and significance level, as indicated in the key (**B**). Stacked bar charts represent the percentages of proteins as linked to amyloid and tau, synaptic, vascular, neurodegeneration and inflammation in amyloid-positive and amyloid-negative individuals. The analysis is restricted to proteins identified as significantly differentially expressed between amyloid-positive and amyloid-negative individuals. Colors correspond to the processes as indicated in the figure key. **C** The top enriched biological processes identified by Gene Ontology (GO) enrichment analysis using clusterProfiler. The analysis is restricted to proteins identified as significantly differentially expressed between amyloid-positive and amyloid-negative individuals, with upregulated and downregulated proteins analyzed separately. P values were adjusted for multiple comparisons (FDR). Bars show −log10(adjusted P value), positive values indicate enrichment among proteins higher in amyloid-positive participants and negative values indicate enrichment among proteins higher in amyloid-negative participants. **D** Estimated effects (β) of pathology groups on amyloid-related protein levels, shown as points with 95% CIs; vertical dashed line marks no effect (β = 0). Each point represents the β estimate from the linear regression model and horizontal lines indicate the corresponding 95% CI. Models were adjusted for amyloid-β status, age, sex and average protein level. Significance: * *p* < 0.05; ** FDR *q* < 0.05 (BH). Source data are provided as a Source Data file. BH Benjamini–Hochberg, CI confidence interval, FDR false discovery rate, TNF tumor necrosis factor.

When comparing amyloid-positive with amyloid-negative individuals, 22 proteins were found differentially upregulated in amyloid-positive individuals and 14 proteins were differentially upregulated in amyloid-negative individuals, of which in total 20 passed multiple testing correction. Proteins including p-tau231, p-tau181, p-tau217, MAPT, FABP3, KLK6, IGF1R, IL5, CX3CL1, VCAM1 and IL33 were found elevated in amyloid-positive individuals (Padj <0.05), while IL6R, TIMP3, HTT, CD40LG, ANXA5,VGF, ENO2, BDNF had lower levels in comparison with amyloid-negative individuals (Padj <0.05) (Fig. 2A and Supplementary Table 4). At a descriptive level, the amyloid-negative individuals showed proteomic profiles majorly linked to neurodegeneration, synaptic and vascular pathologies, while the amyloid-positive individuals showed proteomic profiles majorly linked to inflammation and amyloid and tau pathology (Fig. 2B). When further profiling the significant proteins based on the biological processes they are involved with (Fig. 2C, Supplementary Table 5), the proteins found elevated in plasma of amyloid-positive individuals were mostly associated with immune and developmental processes. The most significant immune-related pathways included leukocyte (Padj = 9.30×10^−05^) and microglial cell activation (Padj = 9.30×10^−05^). These pathways involved proteins already reported to be involved in AD, namely MAPT, CX3CL1, IL13 and IL33. Processes involved in developmental pathways included neuroinflammatory response (Padj = 1.83×10^−04^) and cellular developmental process (Padj = 2.69×10^−04^), of which proteins involved were CX3CL1, FABP3, IGF1R, IGFBP7 and VCAM1. Besides, the proteins found elevated in plasma of amyloid-negative individuals were mostly associated with signaling and enzymatic regulation. The most significant signaling related pathways included nerve growth factor receptor binding (Padj = 3.46×10^−04^) and tumor necrosis factor receptor superfamily binding (Padj = 3.46×10^−05^), of which the proteins involved included BDNF, NGF and CD40LG. Processes involved in enzymatic regulation were mostly represented by metalloendopeptidase inhibitor activity (Padj = 1.10×10^−03^), in particular by NFG and TIMP3.

Next, the influence of comorbidities on the differentially expressed proteins was assessed (Fig. 2D). These analyses accounted for plasma p-tau217 levels (Simoa), age and sex as covariates. Cholesterol was significantly associated with five proteins, with largest effect sizes observed on BDNF (*β* = −0.23, 95% CI [−0.45, −0.01], *P* = 0.04) and HTT (*β* = −0.18, 95% CI [−0.33, −0.03], *P* = 0.02) in which high cholesterol was linked to lower protein levels. Seven proteins showed significant negative associations with heart diseases. The largest effect sizes were observed on BDNF (*β* = 0.25, 95% CI [0.03, 0.48], *P* = 0.03) and ANXA5 (*β* = −0.35, 95% CI [−0.69, −0.01], *P* = 0.04). Infectious diseases were found to affect the plasma levels of ten proteins. The occurrence of infectious diseases has shown largest effect in high levels of plasma FCN2 (*β* = 0.70, 95% CI [0.15, 1.26], *P* = 0.01) and high levels of plasma ANXA5 (*β* = 0.49, 95% CI [0.14, 0.85], *P* = 0.007). Only two proteins showed significant associations with respiratory diseases: IL6R (*β* = 0.44, 95% CI [0.002, 0.88], *P* = 0.05) and IGFBP7 (*β* = 0.12, 95% CI [0.008, 0.23], *P* = 0.04). Similarly, metabolic diseases had a positive effect on higher levels of CX3CL1 (*β* = 0.23, 95% CI [0.05, 0.40], *P* = 0.01) and KLK6 (*β* = 0.18, 95% CI [0.008, 0.35], *P* = 0.04). No associations between proteins and other conditions could be observed. Detailed reports related to these analyses were included in Supplementary Table 6, Supplementary Table 7 and Supplementary Fig. 1. Importantly, when comparing amyloid-positive with amyloid-negative individuals in both VALIANT and the Tanzanian cohorts, proteins, such as p-tau217, p-tau231, p-tau181, MAPT, NEFL, NRGN, NPTX2, CX3CL1 and FABP3 were consistently found upregulated in amyloid-positive compared to amyloid-negative participants, while proteins, such as HTT, PARK7 and S100A12 were consistently downregulated. Detailed reports related to the Tanzanian analyses were included in (Supplementary Fig. 2A, B and Supplementary Table 8).

Furthermore, the use of the two different cutoffs for amyloid positivity within the VALIANT cohort (Simoa p-tau217 cutoff or as defined by NULISA p-tau217 values exceeding the 70th percentile in cognitively unimpaired individuals) yielded similar results; proteins including p-tau231, p-tau181, p-tau217, MAPT, FABP3, KLK6 and FABP3 were found elevated in amyloid-positive individuals (Padj <0.05), while IL6R, TIMP3, HTT, CD40LG, ANXA5, BDNF had lower levels in comparison with amyloid-negative individuals (Padj <0.05) (Supplementary Fig. 2C, D, Supplementary Table 9).

### Proteomic profiles related to cognitive status in amyloid-positive participants

**Fig. 3:**
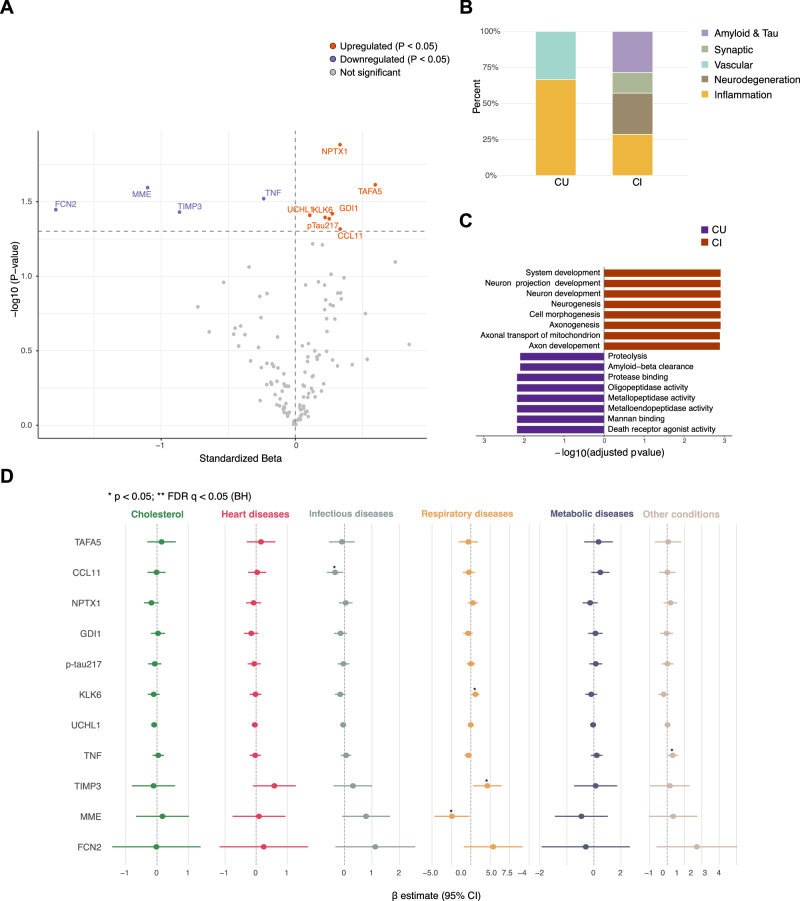
Characterization of proteomic profiles related to cognitive status in amyloid-positive participants. **A** The volcano plot displays the standardized beta values of the protein associations in plasma (x-axis) against statistical significance −log10(P value) (y-axis) between CU (*N* = 119) and CI (*N* = 33) in amyloid-positive individuals. Models were adjusted for age, sex and average of the proteins. P values were derived from two-sided tests of the regression coefficient. Multiple comparisons were adjusted using the Benjamini–Hochberg (BH) false discovery rate (FDR) method. Significant proteins are colored according to direction of association and significance level, as indicated in the key. **B** Stacked bar charts represent the percentages of proteins as linked to amyloid and tau, synaptic, vascular, neurodegeneration and inflammation in CU and CI individuals. The analysis is restricted to proteins identified as significantly differentially expressed between CU and CI in amyloid-positive individuals. Colors correspond to the processes as indicated in the figure key. **C** The top enriched biological processes identified by Gene Ontology (GO) enrichment analysis using clusterProfiler. The analysis is restricted to proteins identified as significantly differentially expressed between CU and CI in amyloid-positive individuals, with upregulated and downregulated proteins analyzed separately. P values were adjusted for multiple comparisons (FDR). Bars show −log10(adjusted P value), positive values indicate enrichment among proteins higher in CI amyloid-positive participants and negative values indicate enrichment among proteins higher in CU amyloid-positive participants. **D** Estimated effects (β) of pathology groups on CU and CI protein levels in amyloid-positive individuals, shown as points with 95% CIs; vertical dashed line marks no effect (β = 0). Each point represents the β estimate from the linear regression model and horizontal lines indicate the corresponding 95% CI. Models were adjusted for amyloid-β status, age, sex and average protein level. Significance: * *p* < 0.05; ** FDR *q* < 0.05 (BH). Source data are provided as a Source Data file. BH Benjamini–Hochberg, CI confidence interval, FDR false discovery rate.

To better understand which plasma proteins could be reflective of cognitive decline in the likely presence of amyloid pathology, protein levels were contrasted in amyloid positive individuals with and without cognitive impairment. Compared with cognitively unimpaired (CU), seven proteins had higher expression in cognitively impaired individuals (CI) while 4 proteins had lower expression, although none of them passed multiple testing correction (Fig. 3A and Supplementary Table 10). At a descriptive level, the CU amyloid-positive individuals showed proteomic profiles majorly linked to neuroinflammation and vascular pathologies, while the CI amyloid-positive individuals showed proteomic profiles majorly linked to neurodegeneration, synaptic and amyloid and tau pathology (Fig. 3B). When further profiling the significant proteins based on the biological processes they were involved with (Fig. 3C, Supplementary Table 11), the proteins found elevated in plasma of CU amyloid-positive individuals were mostly associated with protein metabolic process and signaling receptor binding. The most significant protein metabolic process pathways included amyloid-beta clearance (Padj = 8.05×10^−03^) and proteolysis (Padj = 8.05×10^−03^), processes disrupted in the AD context. These pathways involved proteins, such as MME, TNF and TIMP3. Besides, the proteins found elevated in plasma of CI amyloid-positive individuals, were mostly associated with developmental process and cell component organization. The most significant signaling related pathways axonogenesis (Padj = 1.23×10^−03^) and neurogenesis (Padj = 1.23×10^−03^) of which the main proteins involved included KLK6, NPTX1, MAPT and UCHL1. Processes involved in cell component organization were mostly represented by neuron development (Padj = 1.23×10^−03^) and axon development (Padj = 1.28×10^−03^), in particular by GDI1, NPTX1, MAPT and UCHL1. Next, we tested the associations between the proteins and comorbidities (Fig. 3D). For other conditions, only one protein showed significant associations: TNF (*β* = 0.32, 95% CI [0.02, 0.62], *P* = 0.04) and similarly for infectious diseases: CCL11 (*β* = −0.34, 95% CI [−0.63, −0.04], *P* = 0.02). For respiratory diseases, three proteins showed significant associations. The largest effect sizes were observed for TIMP3 (*β* = 2.12, 95% CI [0.27, 3.96], *P* = 0.02) and MME (*β* = −2.44, 95% CI [−4.72, −0.18], *P* = 0.03). No associations between proteins and cholesterol, heart diseases or metabolic diseases were observed. Effects of comorbidities on protein levels (Supplementary Table 12), and comparison of plasma protein expression after adjusting for all comorbidities (Supplementary Table 13 and Supplementary Fig. 3) are included for more clarity.

### Proteomic profiles related to cognitive status in amyloid-negative participants

**Fig. 4:**
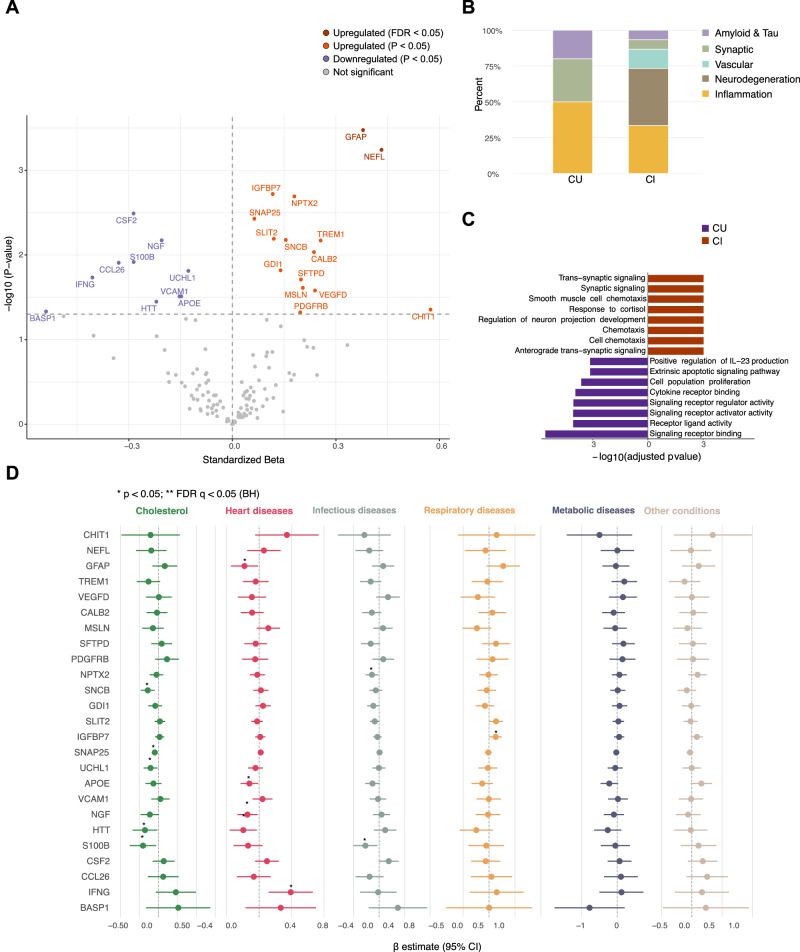
Characterization of proteomic profiles related to cognitive status in amyloid-negative participants. **A** The volcano plot displays the standardized beta values of the protein associations in plasma (x-axis) against statistical significance −log10(P value) (y-axis) between CU (*N* = 692) and CI (*N* = 105) in amyloid-negative individuals. Models were adjusted for age, sex and average of the proteins. P values were derived from two-sided tests of the regression coefficient. Multiple comparisons were adjusted using the Benjamini–Hochberg (BH) false discovery rate (FDR) method Significant proteins are colored according to direction of association and significance level, as indicated in the key. **B** Stacked bar charts represent the percentages of proteins as linked to amyloid and tau, synaptic, vascular, neurodegeneration and inflammation in CU and CI individuals The analysis is restricted to proteins identified as significantly differentially expressed between CU and CI in amyloid-negative individuals. Colors correspond to the processes as indicated in the figure key. **C** The top enriched biological processes identified by Gene Ontology (GO) enrichment analysis using clusterProfiler. The analysis is restricted to proteins identified as significantly differentially expressed between CU and CI in amyloid-negative individuals, with upregulated and downregulated proteins analyzed separately. P values were adjusted for multiple comparisons (FDR). Bars show −log10(adjusted P value), positive values indicate enrichment among proteins higher in CI amyloid-negative participants and negative values indicate enrichment among proteins higher in CU amyloid- negative participants. **D** Estimated effects (β) of pathology groups on CU and CI protein levels in amyloid-negative individuals, shown as points with 95% CIs; vertical dashed line marks no effect (*β* = 0). Each point represents the β estimate from the linear regression model and horizontal lines indicate the corresponding 95% CI. Models were adjusted for amyloid-β status, age, sex and average protein level. Significance: * *p* < 0.05; ** FDR *q* < 0.05 (BH). Source data are provided as a Source Data file. BH Benjamini–Hochberg, CI confidence interval, FDR false discovery rate, IL − 23= interleukin-23.

To better comprehend if plasma protein profiles could indicate the presence of cognitive impairment, likely unrelated to underlying amyloid pathology, amyloid-negative individuals with and without cognitive impairment were compared. In the plasma of cognitively impaired compared to cognitively unimpaired (CU), we observed 15 differential upregulated proteins in cognitively impaired individuals (CI) and 10 differential upregulated proteins in cognitively unimpaired individuals, of which in total two passed multiple testing correction. Proteins including GFAP and NEFL were found elevated in CI amyloid-negative individuals (Padj <0.05) (Fig. 4A and Supplementary Table 14). At a descriptive level, the CU amyloid-negative individuals showed proteomic profiles majorly linked to amyloid and tau, synaptic and neuroinflammation pathology, while the CI amyloid-negative individuals showed proteomic profiles majorly linked to neurodegeneration and vascular pathology (Fig. 4B). When further profiling the significant proteins based on the biological processes they are involved with (Fig. 4C and Supplementary Table 15), the proteins found elevated in plasma of CU amyloid-negative individuals were mostly associated with signaling receptor binding. The most significant protein metabolic process pathways included signaling receptor binding (Padj = 5.40×10^−06^) and receptor ligand activity (Padj = 1.45×10^−04^). These pathways involved proteins, such as IFNG, CCL26, CSF2, NGF and APOE. Similarly, the proteins found elevated in plasma of CI amyloid-negative individuals, were mostly associated with signaling, as well as chemotaxis. The most significant signaling related pathway was synaptic signaling (Padj = 1.40×10^−03^) of which the main proteins involved included SNAP25, SNCB, NPTX2, CALB2, GFAP and NEFL. Processes involved in chemotaxis were mostly represented by cell chemotaxis (Padj = 1.40×10^−03^), in particular by SLIT2 and PDGFRB. Next, we tested the associations between the proteins and comorbidities (Fig. 4D). For cholesterol, five proteins showed significant associations. The largest effect sizes were observed for S100B (*β* = −0.20, 95% CI [−0.38, −0.03], *P* = 0.02) and HTT (*β* = −0.18, 95% CI [−0.34, −0.01], *P* = 0.03). Directionally, effects were exclusively negative. For heart diseases, 5 proteins showed significant associations. The largest effect sizes were observed for IFNG (*β* = 0.39, 95% CI [0.11, 0.66], *P* = 0.005) and HTT (*β* = −0.19, 95% CI [−0.36, −0.03], *P* = 0.02). Directionally, effects were predominantly negative. For infectious diseases, two proteins showed significant associations: S100B (β = −0.21, 95% CI [−0.40, −0.02], *P* = 0.03) and NPTX2 (β = −0.11, 95% CI [−0.21, −0.01], *P* = 0.02). Directionally, effects were exclusively negative. For respiratory diseases, only one protein showed significant associations: IGFBP7 (*β* = 0.12, 95% CI [0.01, 0.25], *P* = 0.03). No associations between proteins and other conditions or metabolic diseases could be observed. Effects of comorbidities on protein levels (Supplementary Table 16), and comparison of plasma protein expression after adjusting for all comorbidities (Supplementary Table 17 and Supplementary Fig. 4) are included for more clarity.

### Proteomic changes and sex differences in protein levels

**Fig. 5:**
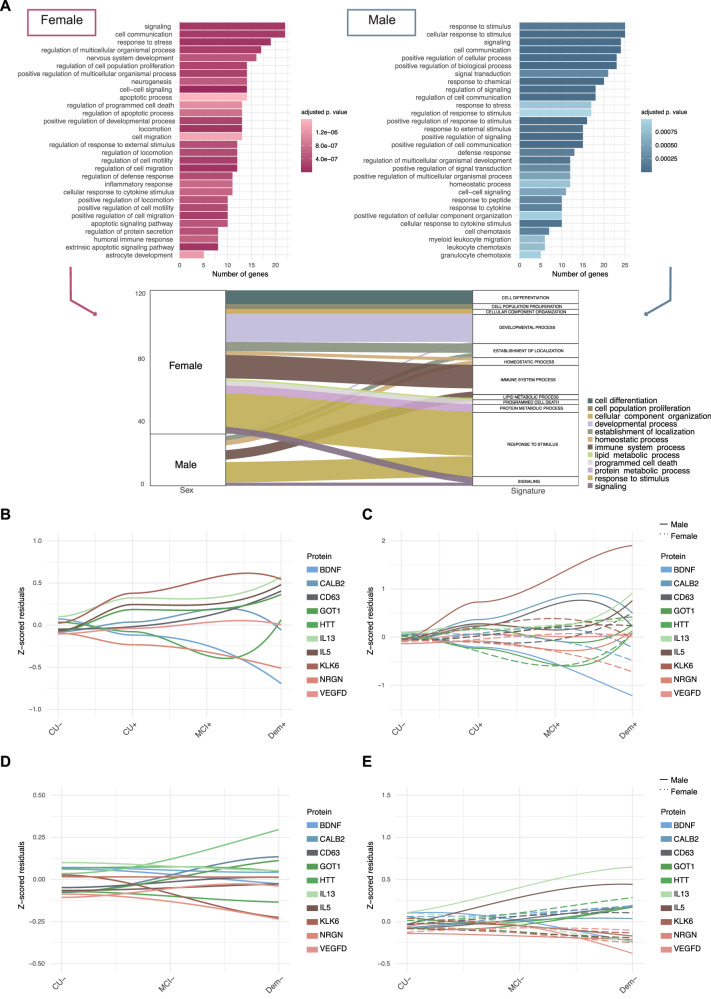
Proteomic changes and sex differences in protein levels. **A** Barplot showing the top 30 GO terms for females (pink) and males (blue). GO enrichment analysis was performed as a functional enrichment analysis using clusterProfiler, with default parameters. The results were adjusted for multiple comparisons (FDR) using the Benjamini–Hochberg method. The bars represent the number of genes involved, with colors indicating the adjusted *p*-values (FDR) (gradient from lighter to darker indicating increasing significance). The *x*-axis shows the GO term descriptions, ordered by number of genes, and the *y*-axis represents the pathways involved. GO BP terms were clustered by semantic similarity and represented the similarity matrices as GO networks. The alluvial represents the link of each sex with biological terms (BT) identified, with line thickness reflecting the number of pathways contributing to the biological terms. Colors correspond to the processes as indicated in the figure key. **B–E** Biomarker trajectories using a local weighted regression method (Loess curve). Changes in plasma biomarker levels are represented as Z-scores (anchored to the CU-). **B** Loess represents changes of the biomarkers in amyloid-positive participants. **C** Loess represents changes of the biomarkers in amyloid-positive participants, stratified by sex. Solid lines represent male participants, while dashed lines represent female participants. **D** Loess represents changes of the biomarkers in amyloid-negative participants **E** Loess represents changes of the biomarkers in amyloid-negative participants, stratified by sex. Solid lines represent male participants, while dashed lines represent female participants. Source data are provided as a Source Data file. CU- = cognitively unimpaired amyloid-negative; CU + = cognitively unimpaired amyloid-positive; Dem-= dementia amyloid-negative; Dem + = dementia amyloid-positive; MCI-= mild cognitive impairment amyloid-negative; MCI + = mild cognitive imparement amyloid-positive.

Although not the primary objective of this work, sex differences in protein levels were briefly explored. Gene ontology (GO) enrichment (Fig. 5A) revealed pathways, such as cell differentiation, lipid and protein metabolic processes to be enriched in females, while male participants showed enrichment in a smaller number of pathways and broader signatures, such as signaling and homeostasis pathways. Immune system processes were enriched in both sexes, with a major contribution in females. When exploring sex differences at the protein level, 10 proteins showed significant differences between females and males, with IL5, BDNF, IL13, HTT, KLK6, CD63, GOT1 showing higher levels in males (*p* < 0.05), and CALB2, NRGN, VEGFD, and CXCL1 showing higher levels in females (*p* < 0.05) (Supplementary Fig. 5). Thereafter, levels of significant proteins were assessed throughout a disease pseudo-time, in amyloid-positive and amyloid-negative individuals (Fig. 5B–D). The locally estimated scatterplot smoothing (Loess) curves suggested trends towards increased protein changes in the amyloid-positive subset compared to the amyloid-negative subset, throughout the disease pseudo-time. When stratifying the participants according to amyloid-positivity, the Loess suggested trends towards greater changes in males, while female individuals suggested trends towards lower changes throughout the disease pseudo-time, both in the amyloid-negative and amyloid-positive subsets. Analyses within disease groups supported these differences. Proteins, such as IL5, and VEGDF showed an increase from CU- to MCI- (*p* < 0.05), and IL13 an increase from CU- to Dem - (*p* < 0.05) in the male individuals, while CALB2 showed an increase from CU- to MCI- (*p* < 0.05) and from from CU- to Dem- (*p* < 0.05) in female individuals (Supplementary Fig. 6A). Differences could be also identified in the amyloid-positive comparisons, with KLK6 showing more marked increases from CU+ to MCI+ (*p* < 0.01) and from from CU+ to Dem+ (*p* < 0.05) in males, and CALB2 showing higher increases from CU+ to MCI+ (*p* < 0.05) in females (Supplementary Fig. 6B). The boxplots of sex differences within each subgroup, both for amyloid-negative and amyloid-positive subsets are included in (Supplementary Fig. 7 and Supplementary Fig. 8).

### Proteomic changes differ between cohorts

**Fig. 6:**
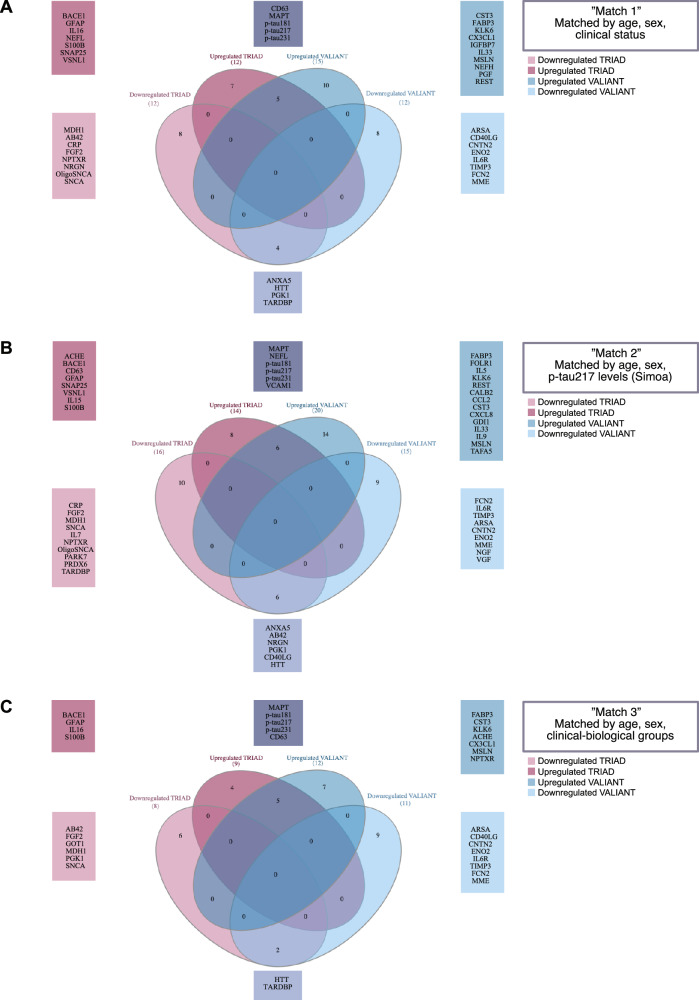
Proteomic changes differ between VALIANT and TRIAD cohorts. **A** Venn diagram summarizing the comparison of significant proteins derived from the comparison between amyloid-positive and amyloid-negative individuals in VALIANT and TRIAD cohorts for Match 1. Cohorts were matched by age, sex and clinical status (cognitively unimpaired (CU), MCI, Dementia). Intersections indicate proteins shared between cohorts, while non-overlapping regions indicate cohort-specific proteins. Colors correspond to the cohorts as indicated in the figure key. **B** Venn diagram summarizing the comparison of significant proteins derived from the comparison between amyloid-positive and amyloid-negative individuals in VALIANT and TRIAD cohorts for Match 2. Cohorts were matched by age, sex, and plasma p-tau217 levels (Simoa). Intersections indicate proteins shared between cohorts, while non-overlapping regions indicate cohort-specific proteins. Colors correspond to the cohorts as indicated in the figure key. **C** Venn diagram summarizing the comparison of significant proteins derived from the comparison between amyloid-positive and amyloid-negative individuals in VALIANT and TRIAD cohorts for Match 3. Cohorts were matched by age, sex, and clinicopathological groups. Intersections indicate proteins shared between cohorts, while non-overlapping regions indicate cohort-specific proteins. Colors correspond to the cohorts as indicated in the figure key. Source data are provided as a Source Data file. TRIAD Translational Biomarkers in Aging and Dementia, VALIANT Vascular health, frailty, and cognition in Ageing Nigerians study.

Next, we aimed at exploring to what extent proteomic profiles would overlap or be different across two cohorts with different ethnic and racial backgrounds. When assessing the comparison of significant proteins between amyloid-positive and amyloid-negative individuals in VALIANT and TRIAD cohorts in all the Match datasets (Fig. 6A–C), proteins, such as p-tau217, p-tau181, p-tau231, and MAPT were consistently upregulated both in VALIANT and TRIAD cohorts in amyloid-positive compared to amyloid-negative participants, while proteins, such as ANXA5 and HTT, were consistently downregulated in both cohorts in the same group. Some proteins, such as KLK6, CST3, FABP3 and MSLN showed upregulation uniquely in VALIANT cohort in amyloid-positive participants, compared to amyloid-negative participants, while others, such as ARSA, CNTN2, ENO2, IL6R, TIMP3 showed downregulation uniquely in VALIANT cohort in amyloid-positive participants, compared to amyloid negative participants.

## Discussion

The present study explored plasma biomarker profiles of AD pathology and cognitive status in a population-based African cohort of older adults. Several proteins were found to be differentially abundant in the plasma of amyloid-positive individuals and in the different cognitive groups, unveiling the altered expression patterns in different stages of the disease and further evidencing the relevance of core plasma biomarkers regardless of ethnic, racial, and socioeconomic backgrounds. Such findings were consistently observed in all cohorts, but more similarities in protein expression were found among the African cohorts as compared with the North American cohort. Comorbidities, particularly heart disease, cholesterol, and infectious diseases, were shown to have an effect on some protein levels. Additionally, biomarkers showed different trends in females and males throughout the disease stages. The current research scenario has shown the value and the relevance of plasma biomarkers as tools for AD diagnosis, which would especially benefit the low- and middle-income countries (LMICs), where the limited resources hinder the use of PET imaging or cerebrospinal fluid (CSF) biomarker assays. Markers, such as phosphorylated tau (p-tau) have shown utility in identifying AD at early stages, and particularly p-tau217 will represent an essential marker from a clinical standpoint^23–25^. However, p-tau markers do not entirely explain the etiology of this multifactorial disease, characterized by several other processes, such as synaptic disruption and neuroinflammation^26^. In this endeavor, GFAP and NfL have been shown to reflect processes, such as astrocytic activation and neurodegeneration^27,28^, but additional markers are needed to gain a more comprehensive understanding of the disease, along with improving disease staging and monitoring.

Our findings from the comparison of amyloid-positive and amyloid-negative individuals reinforced the utility of p-tau biomarkers as the leading biomarkers in plasma (p-tau217, p-tau231, and p-tau181) in the context of amyloid pathology and AD and were consistent with other investigations conducted with the NULISA method^29–31^. Such findings were observed in both African cohorts, with p-tau217, p-tau181, and p-tau231 appearing as the most significant biomarkers. Interestingly, although all plasma p-tau markers were significantly associated with amyloid pathology status, p-tau181 and p-tau231 showed larger effect sizes than p-tau217. A similar pattern was observed in the Tanzanian cohort. However, since amyloid PET data were unavailable for these cohorts, and amyloid pathology status was proxied using plasma p-tau217, direct comparisons will require further investigations. Importantly, the quantification of these biomarkers did not seem to be impacted by the presence of the comorbidities here tested. Although associations between core biomarkers were replicated as expected, further studies using CSF or PET biomarkers as the standard of truth for amyloid pathology are needed to further confirm these associations.

Additionally, in concordance with previous work^11^, ancillary biomarkers of AD like MAPT (responsible for encoding tau protein) and proteins related to immune response (IL5, IL13, IL33, CX3CL1, IGF1R, KLK6, FABP3, and VCAM1)^32^ were found to be upregulated among amyloid-positive participants. As for the amyloid-negative subset, upregulation of proteins, such as IL6R and TIMP3, was in concordance with previous research in AD, showing a reduction in response to amyloid in plasma and CSF^33,34^. Additionally, TIMP3 has been shown to play a role in small vessel disease (SVD) in mouse models^35^. Indeed, gene ontology unveiled the relevance of these proteins mainly in cell-to-cell communication and brain homeostasis maintenance, processes consistent with healthy brain functions, when amyloid pathology is absent. It is important to highlight that proteomic platforms, including additional proteins, might expand the identification of potentially novel proteins beyond established AD-related pathways^36,37^. Therefore, future studies leveraging larger and more comprehensive panels may further expand the spectrum of amyloid-associated proteins, particularly in a context such as that in Africa.

When contrasting CU and CI amyloid-positive individuals, proteins related to synaptic dysfunction (NPTX1), inflammation (CCL11, KLK6) were upregulated in the CI status, highlighting the involvement of processes related to protein degradation and apoptosis in this context. Repeatedly, TIMP3 was upregulated in the CU amyloid-positive individuals and linked with an involvement in catalytic activity and protein metabolic process, potentially involving the efficiency of amyloid degradation as well as potential compensatory mechanisms. When contrasting CU and CI amyloid-negative individuals, proteins involved in vascular, inflammatory (GFAP, TREM1), synaptic (NPTX2, SNAP25), vascular (PDGFRB), and neurodegenerative (NfL) mechanisms were overexpressed in CI individuals, highlighting the relevance of these proteins in relation to cognitive impairment. Proteins downregulated in CI amyloid-negative mostly consisted of pro-inflammatory signatures. Interestingly, UCHL1 showed an opposite association with cognitive function depending on amyloid status. UCHL1 is a neuron-specific protein that in physiological conditions has been implicated in synaptic plasticity and proteostasis^38–40^. In our results, UCHL1 levels did not differ between CU amyloid-negative and amyloid-positive individuals, suggesting physiological levels at the preclinical stage regardless of amyloid status. In contrast, divergent patterns were observed in cognitively impaired individuals, with decreased UCHL1 in CI amyloid-negative and increased levels in CI amyloid-positive relative to their respective CU groups, suggesting that different pathological processes may underlie these changes. Potentially, reduced UCHL1 might indicate synaptic loss in amyloid-negative impairment, while increased levels in amyloid-positive individuals might reflect neuronal stress or increased release associated with neurodegeneration.

An important factor when interpreting biomarker profiles is the high proportion of individuals living with multimorbidity^41^, which is associated with poorer health outcomes and adds complexity to the biological landscape of dementia. In our study, several differentially expressed proteins were also associated with high cholesterol and heart disease, in line with literature implicating lipid dysregulation and vascular pathology as important contributors to AD^42–46^. Notably, proteins, such as KLK6, NRGN, and PGK1 were associated with a previous history of infectious diseases—predominantly malaria—independent of plasma p-tau217 levels. Beyond the documented long-term cognitive effects of malaria, a recent study has reported differential expression of these same proteins during acute malaria infection relative to healthy controls, lending biological support to our observations^47^.

Nevertheless, interpreting these associations remains challenging. They may reflect long-lasting neuroinflammatory or neuroinjury processes triggered by early or repeated infections, potentially increasing susceptibility to AD pathology later in life, as previously proposed^48–50^. Furthermore, these associations may also be influenced by socioeconomic and cultural factors that shape exposure to infections, access to healthcare, and lifestyle patterns. Many individuals in African settings are affected by multiple concurrent conditions—including infectious diseases—and recent economic transitions have contributed to lifestyle changes including changing of dietary patterns, increased alcohol consumption and tobacco use, which in turn elevate the risk of metabolic and cardiovascular disorders^51^.

As biomarker-based approaches move toward clinical implementation, it will be essential to systematically record and integrate comorbidities to improve diagnostic specificity and accuracy^52,53^. In our analyses, the overall effect of comorbidities on protein levels was modest, although heart disease, infectious disease history, and hypercholesterolemia exerted the most notable influence. These findings suggest that certain proteins may require careful interpretation in the presence of specific comorbid conditions. Another important aspect to consider is the potential difference in biomarker levels between females and males. Previous research has highlighted sex-related differences in AD, both in terms of cognitive psychiatric symptoms, where females usually showed a faster decline as the disease progresses, as well as in biomarker levels^54,55^. These differences reflect the distinct metabolic, cerebrovascular and socio-economic risk factors^56^. All these factors hold notable importance in the African context, considering that gender-specific differences might be influenced by sociocultural and economic factors, directly impacting on health outcomes^3^. Besides, these differences might also be due to distinct biological features inherent to the African ancestry population. In accordance with this, our findings highlighted specific proteins with higher levels in males compared to females (such as KLK6), or otherwise with higher levels in females compared to males (such as CALB2) throughout the disease stages. This might reflect the forementioned factors and will need further evaluation in future studies in the African context.

Last, the comparison between the African and North American cohorts aimed at exploring which biomarkers are relevant to AD pathology regardless of ethnic, racial, and socioeconomic conditions. Regardless of how the matching was performed between the two populations, tau-related biomarkers and CD63 were positively associated with plasma p-tau217 positivity, while ANXA5, HTT, PGK1, and TARDBP were negatively associated in both cohorts. As regards the associations that were not concordant between populations, several questions remain unanswered. Did these discrepancies arise because expression levels are indirectly influenced by socioeconomic factors, technical variability, or other unmeasured confounders, or were they simply due to limited statistical power? These are questions that require further studies.

This study leverages a unique cohort and constitutes the largest sample size of any AD biomarker study conducted in Africa to date, allowing for the investigation of proteomic profiles in this setting. The nature of the study provides insights into the AD biomarker scenario in underrepresented groups, like the ones in Africa. However, strengths are balanced by several limitations that highlight directions for future research. First, this work focused solely on plasma biomarkers, and the lack of PET and CSF data to confirm amyloid status reflects current limitations in local resources and infrastructure. To mitigate this issue, we applied a previously validated cut-off based on plasma p-tau217 levels, to determine amyloid pathology^57^. In this context, the low proportion of amyloid-positive individuals likely reflects the design of the study, based on a population-based cohort of community dwelling older adults aged > 50 years. As the prevalence of amyloid pathology is strongly age-dependent^58,59^, the lower prevalence of p-tau217 positivity in the VALIANT cohort could be explained by the younger age of inclusion in the study. Additionally, the cohort showed predominance of CU individuals, rather than MCI and dementia, and females rather than males. The higher proportion of CU participants, as compared to other AD research cohorts can also be potentially explained by the age criteria for inclusion in the study. Furthermore, differences in survival following dementia diagnosis across settings—potentially impacted by higher mortality in low- and middle-income countries—may contribute to variation in the proportion of diagnostic groups observed between research- and population-based studies between the cohorts here studied. As for what concerns the prevalence of women, this reflects both the design of the study and sociocultural characteristics of the Nigerian population. As participants were met in their homes, women were often more available to participate in the study than men, partly because many female participants were petty traders or housewives who did not work outside the home. These demographic and clinical differences highlight important distinctions between the present cohort and the predominantly North American cohorts commonly represented in AD research, such as TRIAD. To attenuate these differences and improve comparability while retaining an adequate sample size, neighbor propensity score matching was applied to pair participants across c3ohorts prior to statistical analyses. Nevertheless, exact matching between cohorts was not fully achievable, and despite adjustment for relevant covariates in the models, this limitation should be acknowledged when interpreting the findings. Additionally, given the substantial ethnic and sociodemographic heterogeneity across the African continent, it is important to note that this study was conducted in representative populations from Nigeria and rural Tanzania, and findings may not generalize fully to all African regions. Finally, further proteomic profiling in other African cohorts, as well as genetic profiling, will be required in the future, to gain more in-depth insights into the AD proteomic scenario in the African population.

Overall, leveraging proteomic data, we provided insights on key proteins and molecular pathways that co-occur in AD in the African settings. These data provide resource for further investigation on AD biomarkers in different racial/ethnic groups, emphasizing the importance of distinct genetic and social factors, as well as multimorbidity, ultimately reflecting on the proteomic level and consequently on the diagnostic and therapeutic strategies. This study addresses key AD biomarker generalizability research gaps and establishes groundwork for the future equitable AD biomarker use and treatment across diverse populations.

## Methods

### Study design and participants

This study included participants from Nigeria (VALIANT cohort), Tanzania (Hai Dementia study) and Canada (TRIAD cohort). VALIANT study obtained ethical approval from the University of Ibadan/University College Hospital (UI/UCH) Health Research Ethics Committee (HREC) and all participants provided written informed consent prior to participation. Ethical approval for the Tanzanian study was granted by the National Institute for Medical Research, Tanzania. Informed consent for each participant was obtained after verbal explanation of the information sheet from a Tanzanian medical officer or nurse. Individuals unable to give written consent due to illiteracy or severe visual impairment indicated consent using a thumbprint. Where participants were deemed to lack capacity to consent due to cognitive impairment, assent was taken from a close relative provided the participant actively demonstrated willingness to participate. TRIAD was approved by the MNI PET working committee and the Douglas Mental Health University Institute Research Ethics Board (MP-18-2017-157). Written informed consent was obtained for all participants.

The Vascular heAlth, fraiLty and cognition in Ageing Nigerians (VALIANT) study is a Nigerian community-based longitudinal study focusing on the investigation of the association between cardiovascular health, cognition, and frailty. 1031 study participants ( ≥ 50 years of age) from an urban community in Yemetu, Ibadan North Local Government Area, Oyo State, Southwest Nigeria, were recruited using a multistage cluster sampling method. Details on the recruitment of the participants has been described previously^60^. Nine hundred and forty nine participants had available blood-based biomarkers and sociodemographic data.

To replicate the main analysis, participants from the rural Hai district of northern Tanzania were included. The cohort consists of community-dwelling adults aged 65 years and over, recruited from six villages within Hai and selected to be representative of upland and lowland areas and different economic status. The study was a community-based door to door dementia prevalence study using the 10/66 protocol. Dementia diagnosis was made by consensus following a detailed interview, and based on the DSM-IV criteria. Mild cognitive impairment (MCI) was defined by Petersen criteria. More detailed information about the recruitment process for this cohort has been previously reported^61–63^. Blood samples were obtained at a home visit, transported on ice within two hours of being taken, spun into aliquots and stored in a −80 freezer at Kilimanjaro Christian Research Institute before transport for proteomic analysis. 196 participants had available blood-based biomarkers and sociodemographic data.

For comparative analysis of the proteomic signatures between the two distinct population groups, participants from the Translational Biomarkers in Aging and Dementia cohort (TRIAD), a longitudinal imaging and biofluid cohort study of aging and AD in Canada, were included. TRIAD cohort consists of individuals who were recruited from a tertiary care memory clinic and cognitively unimpaired individuals who are adult volunteers from the community. These individuals are largely representative of the white population from a high-income country and detailed description of TRIAD’s inclusion and exclusion criteria can be found elsewhere^64^. Match 1 matched VALIANT and TRIAD by diagnosis, age and sex (*n* = 315), Match 2 matched VALIANT and TRIAD by p-tau217, age and sex (*n* = 376) and Match 3 matched VALIANT and TRIAD by clinical*-*biological group, age and sex (*n* = 290).

### Sociodemographic data

For VALIANT cohort, sociodemographic data were collected from all participants, including sex (male and female), age and years of education (primary [< 8 years of studies], secondary [between 8 and 12 years of studies] and beyond secondary [> 12 years of study] education). Gender information was not collected. Sex-stratified analyses were performed in the VALIANT cohort to explore potential sex-related differences in biomarker levels.

For the Tanzanian cohort, sociodemographic data were collected from all participants, including sex (male and female) and age. Information about education were available for 131 participants (66.5%) and categorized as no education at all, 4 years or less of education, more than 4 years of education. Education was coded as 1 for no education and as 0 for some primary education or higher level. Gender information was not collected.

For TRIAD cohort sociodemographic data were collected from all participants, including sex (male and female), age and years of education. Gender information was not collected.

### Cognitive assessment

For VALIANT participants, cognitive function was assessed using Montreal Cognitive Assessment (MoCA), and the Identification and Intervention for Dementia in Elderly Africans (IDEA) cognitive screen^61,65^. The IDEA and MoCA are tests of general cognitive functioning that have been well-validated in the African setting^66,67^. Participants were initially screened for cognitive status using the IDEA ( < 9; cognitively impaired vs cognitively unimpaired)^66^ and MoCA ( < 19; cognitively impaired/cognitively unimpaired)^67^, and functional impairment was assessed using the IDEA-ADL ( < 11)^68^ and FAS scores ( > 9)^69^. Cognitive status [cognitively unimpaired (CU), mild cognitive impairment (MCI) and dementia (Dem)] was determined based on consensus diagnosis given by two neurologists.

For the Tanzanian participants, cognition was assessed at baseline using the IDEA cognitive screen, as described previously^61^.

For TRIAD participants diagnosis consisted in cognitively unimpaired (CU), mild cognitive impairment (MCI), AD dementia, documented as previously described^70^. Specifically, cognitively unimpaired individuals had showed no objective cognitive impairment, a CDR score of 0, and were asked to report any subjective cognitive decline in a questionnaire provided during screening. Individuals with MCI showed relatively preserved activities of daily living, cognitive impairment and a CDR score of 0.5. Patients with Mild-to-moderate Alzheimer’s clinical syndrome dementia had a CDR score between 0.5 and 2 and met the National Institute on Aging—Alzheimer’s Association (NIA-AA) criteria for probable AD determined by a dementia specialist^71,72^.

### Cardiovascular health evaluation (CVH) and evaluation of comorbidities

VALIANT participants underwent a battery of baseline cardiovascular and other health assessments. Components of the CVH metric included blood pressure, fasting glucose, total cholesterol, body mass index (BMI), physical activity, diet, and smoking. Systolic blood pressure and diastolic blood pressure were measured by a mercury sphygmomanometer on the right arm with the subject in a sitting position after 10 min of rest. Venous blood samples were drawn for the measurement of glucose and lipid profiles after an overnight fast. Fasting lipid profile and fasting plasma glucose were assessed using standard laboratory techniques. BMI was calculated from the height and weight measurements of participants. Physical activity, smoking status, and dietary pattern were assessed using a standardized questionnaire. Cholesterol was dichotomized into high if LDL cholesterol was ≥160 mg/dL, or total cholesterol was ≥240 mg/dL; otherwise, it was considered normal. Furthermore, information about other existing or previous conditions was self-reported. These conditions were categorized into distinct groups, namely heart disease including hypertension, stroke, transient ischemic attack (TIA), arrhythmia, atrial fibrillation and haemoglobinopathies; metabolic disease including hyperlipidaemia, diabetes mellitus and obesity; respiratory diseases including chronic bronchitis, bronchitis and asthma/wheezing; infectious disease including infectious/tropical, HIV, tuberculosis and malaria; other conditions including sleep disorders, migraine and chronic kidney disease;. These are reported and summarized in the Supplementary Table 18.

For TRIAD, inclusion criteria for all participants are the ability to speak English or French, good general health (no diseases expected to interfere with study participation over time), absence of claustrophobia, and adequate visual and auditory capacities to follow neuropsychologic evaluation. Additionally, participants were excluded if they had inadequately treated conditions, active substance abuse, recent head trauma, or major surgery, or if they had MRI/PET safety contraindication^73^.

### Blood-based biomarker analysis

Biomarker data were obtained for 949 study participants from VALIANT, 196 study participants from Tanzanian cohort and 376 study participants for TRIAD cohort. For the three cohorts, blood samples were collected according to clinical best practices. For VALIANT, Ten (10) milliliters of whole blood sample were collected at the study site from each participant into EDTA tubes and transported to the laboratory under standard conditions. The blood samples were centrifuged at 4000 x g for 10 min within 2 h of collection and the separated fractions aliquoted into polypropylene tubes (plasma for biomarker analysis, buffy coat for genomic analysis and red cell concentrates for lipid analysis). These were stored frozen at −20 °C for up to 4 weeks and then transferred into −80 °C freezer until shipment for analysis^74^. For the Tanzanian cohort, 10 milliliters of whole blood samples were also collected in EDTA tubes and transported in coolers (ice-boxes) to the laboratory at the Kilimanjaro Clinical Research Institute (KCRI) within ~3 h after collection. They were similarly separated into three fractions (plasma, buffy coat and RBC) after centrifugation at 4 °C, 4000 × g for 10 min and stored at −80oC at KCRI. One ml duplicates of the original plasma samples were eventually received at the Campus for Ageing and Vitality, Newcastle upon Tyne for further analyses. Two hundred and fifty μl aliquots of these plasma samples were then sent to Gothenburg from Newcastle for the NULISA analysis. TRIAD whole blood samples were collected using EDTA-treated tubes, which were gently inverted several times to ensure proper mixing of blood with the anticoagulant. The tubes were centrifuged at 2200 x g for 10 min at 20 °C and the resulting plasma transferred into 1 mL polypropylene tubes and stored at −80 °C until analysis^75^. Plasma samples were shipped frozen on dry ice to and analyzed at the Department of Psychiatry and Neurochemistry, University of Gothenburg, Gothenburg, Sweden. All the biomarker analyses were done blinded to diagnosis and sample information.

For VALIANT and TRIAD cohorts, measurements of plasma p-tau217 were performed using Simoa HD-X instrument (Quanterix, Billerica, MA, USA) at the Department of Psychiatry and Neurochemistry, University of Gothenburg, employing the ALZpath commercial kit (#104371) as described previously^23^ For VALIANT cohort, brain amyloid pathology positivity was defined using a cutoff of >0.42 pg/mL as previously defined^23^.

The three cohorts were also analyzed on the ARGO-HT platform, with the NULISA^TM^ CNS-disease assay, targeting neurodegenerative disease–related targets, inflammation and immune response–related cytokines and chemokines at the Department of Psychiatry and Neurochemistry, University of Gothenburg^31^. Briefly, plasma samples were thawed and centrifuged at 10,000 x g for 10 min. 10uL of samples were plated in 96-well plates and analyzed with Alamar Biosciences CNS Disease Panel. The NULISAseq workflow employs a Hamilton-based automation instrument. After immunocomplex formation, the following steps involved capturing and washing immunocomplexes on paramagnetic oligo-dT beads, followed by release into a low-salt buffer, and capturing and washing on streptavidin beads. Finally, the proximal ends of the DNA strands on each immunocomplex were ligated to generate a DNA reporter molecule, containing both target- and sample- specific barcodes. DNA reporter molecules were pooled and amplified by PCR, purified and sequenced on Illumina NextSeq 2000. A detailed description on control samples, NULISA protein quantification (NPQ) and LOD calculations can be found here^31^.

To allow for comparison with the Tanzanian cohort, another cutoff was employed, where cutoff positivity was defined as values exceeding the 70th percentile of p-tau217 (NULISA) among cognitively unimpaired participants. Such approach was derived by prior meta-analysis, which reported that approximately 30% of the cognitively unimpaired individuals are amyloid PET-positive between 55 and 59 years of age^76^ and applied to both the VALIANT and Tanzanian cohorts. The rationale of use of such cutoff was due to the unavailability of another standard of truth or biomarker in common between the African cohorts.

### Statistical analysis

All statistical analyses and plots were performed on R statistical software version 4.4.0. For descriptive statistics percentages were used for categorical data, and mean (standard deviation (SD)), for continuous data. Distributions across clinical groups were compared using chi-squared for categorical variables, and Kruskal–Wallis tests for continuous variables. Linear models evaluated the differential protein expression between designated groups (amyloid-positive vs. amyloid-negative, CU vs. CI both in the amyloid-positive and amyloid-negative subsets) for which the target proteins were set as the dependent variable and the group as the predictor variable. The analyses were adjusted for age, sex and the individual average of the proteins. The average of the protein quantification, within each individual, was included as covariate in the statistical models to account for interindividual variability in plasma concentration levels^11,77^.Volcano plots reported, using color schemes, the proteins that were statistically significant when contrasting groups either nominally or after false discovery rate (FDR) correction for multiple comparisons. Stacked bar charts showed the proportion of identified DEPs within each category of protein function. Gene Ontology (GO) was performed as a functional enrichment analysis using the R package clusterProfiler, with default parameters^78^. The results were adjusted for multiple comparisons (FDR) using the Benjamini–Hochberg method. Top representative biological terms with adjusted *p(adj)* < 0.05 were visualized using the R package ggpubr. Using the *GOSlim* package^79^, we clustered the GO BP terms by semantic similarity and represented the similarity matrices as GO networks.

Next, we assessed the effect of comorbidities on the significant DEPs previously identified by including them in the original regression model. Each disease was coded as a binary variable (presence or absence) and individual comorbidities were analyzed separately along with five grouped categories: heart disease, metabolic disease, respiratory conditions infectious diseases and other conditions; cholesterol was coded as a binary variable (high or normal). The regression coefficient (β), its 95% confidence interval, and p-value were extracted. P-values were adjusted for multiple comparisons using FDR (Benjamini–Hochberg).

To explore sex differences, we retained the proteins that had a significant effect on sex in the linear model analysis comparing amyloid-positive and amyloid-negative groups. For the proteins related to each sex, GO was performed as a functional enrichment analysis using the R package clusterProfiler, with default parameters^78^. The results were adjusted for multiple comparisons (FDR) using the Benjamini–Hochberg method. Top representative biological terms with adjusted *p* < 0.05 were visualized using the R package ggpubr. Using the *GOSlim* package^79^, we clustered the GO BP terms by semantic similarity and represented the similarity matrices as GO networks. Locally weighted scatterplot smoothing (Loess) analysis was used to explore non-linear trends of sex differences on proteins levels. T-test was applied in the boxplot analysis to evaluate significant sex differences both within sexes and groups.

For both Tanzanian and VALIANT cohort, linear models evaluated the differential protein expression between amyloid-positive vs. amyloid-negative (with cutoff positivity defined as values exceeding the 70th percentile of p-tau217 (NULISA) among cognitively unimpaired participants, as previously described) for which the target proteins were set as the dependent variable and the group as the predictor variable. The analyses were adjusted for age, sex and the individual average of the proteins. Volcano plots reported, using color schemes, the proteins that were statistically significant when contrasting groups either nominally or after false discovery rate (FDR) correction for multiple comparisons. Venn Diagrams were employed to visualize the differences in proteomic signatures in the two cohorts, employing the *InteractiVenn* tool^80^.

TRIAD and VALIANT cohorts were matched by clinical status, age and sex (1), by p-tau217 concentrations, age and sex (2) and by clinical-biological group (clinical status and p-tau217 positivity combined), age and sex (3) employing the nearest neighbor method (R package *matchit*). Linear models evaluated the differential protein expression between designated amyloid-positive vs. amyloid-negative subsets for which the target proteins were set as the dependent variable and the group as the predictor variable. The analyses were adjusted for age, sex and the individual average of the proteins. Venn Diagrams were employed to visualize the differences in proteomic signatures in the two cohorts, employing the *InteractiVenn* tool^80^.

### Reporting summary

Further information on research design is available in the Nature Portfolio Reporting Summary linked to this article.

## Supplementary information

Supplementary Information

Reporting Summary

Transparent Peer Review file

## Source data

Source Data

## Data Availability

Source data are provided with this file. Pseudonymized participant-level data supporting the findings of this study can be shared upon request by contacting the corresponding author, Rufus Akinyemi. Data access will be provided in accordance with applicable ethical approvals, participant consent, and local data protection regulations. Source data are provided with this paper.

## Code availability

All code was written using available packages in R and has been provided at https://github.com/ilariapola/Plasma-proteomic-profiles-of-Alzheimer-s-Disease-and-Neurodegeneration-in-African-Cohorts.
