# Supplementary material for: Plasma proteomic profiles of Alzheimer’s disease and neurodegeneration in African cohorts
Source: Nat Commun. 2026 Jul 1;17:8215. doi: 10.1038/s41467-026-74971-4 (PMC13462778; doi:10.1038/s41467-026-74971-4)

Supplementary Tables

Supplementary Table 1. Demographics of the Tanzanian participants by clinical group.
Continuous variables were compared across diagnostic groups using the Kruskal-Wallis rank-sum test and data presented as mean (SD). Categorical variables were compared using Pearson's chi-squared test and data presented as n (%). Both tests were performed as two-sided omnibus tests. Exact P values are reported where applicable. N(CU)= 116, N(MCI)= 23, N(Dem)= 57.

Aß= amyloid; CU= cognitively unimpaired; Dem= dementia; MCI=mild cognitively impaired; N= number.

	CU (N=116)	MCI (N=23)	Dem (N=57)	p-value
**Sex**				0.77
Male	42 (36%)	7 (30%)	18 (32%)	
Female	74 (64%)	16 (70%)	39 (68%)	
**Age**	78 (8)	83 (9)	84 (9)	4.29e-06
**Education**				0.78
No education	36 (58%)	13 (62%)	31 (65%)	
Education	26 (42%)	8 (38%)	17 (35%)	
**Aß status**				0.21
Negative	81 (70%)	18 (78%)	34 (60%)	
Positive	35 (30%)	5 (22%)	23 (40%)	

Supplementary Table 2. Demographics of the TRIAD participants by clinical group.

Continuous variables were compared across diagnostic groups using the Kruskal-Wallis rank-sum test and data presented as mean (SD). Categorical variables were compared using Pearson's chi-squared test and data presented as n (%). Both tests were performed as two-sided omnibus tests. Exact P values are reported where applicable. N(CU)= 192, N(MCI)= 91, N(Dem)= 93.

Aß= amyloid; CU= cognitively unimpaired; Dem= dementia; MCI=mild cognitively impaired; N= number.

	CU (N=192)	MCI (N=91)	Dem (N=93)	p-value
**Sex**				0.06
Male	63 (33%)	39 (43%)	43 (46%)	
Female	129 (67%)	52 (57%)	50 (54%)	
**Age**	69 (10)	71 (7)	67 (9)	0.01
**Education**	15.2 (3.4)	15.9 (3.7)	14.2 (4.0)	0.01
**Aß status**				1.30e-13
Negative	155 (81%)	44 (48%)	35 (38%)	
Positive	37 (19%)	47 (52%)	58 (62%)	

Supplementary Table 3. Demographics and biomarker information for the matched data between VALIANT and TRIAD cohorts (Match 1, Match 2 and Match 3).
Continuous data are presented as mean (SD), while categorical data are presented as n (%).

Match 1. Cohorts were matched by age, sex and clinical status (cognitively unimpaired (CU), MCI, Dementia). N(CU)= 192, N(MCI)= 91, N(Dem)= 32 both for TRIAD and VALIANT cohorts.

Aß= amyloid; CU= cognitively unimpaired; Dem= dementia; MCI=mild cognitively impaired; N= number.

TRIAD cohort			
	**CU (N=192)**	**MCI (N=91)**	**Dem (N=32)**
**Sex (Male)**	63 (32.8%)	39 (42.9%)	26 (81.3%)
**Age**	69 (± 9.7)	71 (± 7.3)	75 (± 6.3)
**Years of education**	15 (± 3.4)	16 (± 3.7)	15 (± 5.0)
**Aß positive**	37 (19.3%)	47 (51.6%)	23 (71.9%)
**p-tau217 Simoa**	0.40 (± 0.45)	0.64 (± 0.55)	1.3 (± 0.94)

VALIANT cohort			
	**CU (N=192)**	**MCI (N=91)**	**Dem (N=32)**
**Sex (Male)**	90 (46.9%)	15 (16.5%)	8 (25.0%)
**Age**	67 (± 10)	73 (± 8.8)	77 (± 11)
**Years of education**	6.8 (± 4.8)	2.3 (± 3.3)	1.9 (± 3.7)
**Aß positive**	26 (13.5%)	19 (20.9%)	11 (34.4%)
**p-tau217 Simoa**	0.25 (± 0.14)	0.30 (± 0.24)	0.34 (± 0.27)

Match 2. Cohorts were matched by age, sex and plasma p-tau217 levels (Simoa). N(CU)= 192, N(MCI)= 91, N(Dem)= 93 for TRIAD cohort, N(CU)= 305, N(MCI)= 51, N(Dem)= 20 for VALIANT cohort.

Aß= amyloid; CU= cognitively unimpaired; Dem= dementia; MCI=mild cognitively impaired; N= number.

TRIAD cohort			
	**CU (N=192)**	**MCI (N=91)**	**Dem (N=93)**
**Sex (Male)**	63 (32.8%)	39 (42.9%)	43 (46.2%)
**Age**	69 (± 9.7)	71 (± 7.3)	67 (± 9.2)
**Years of education**	15 (± 3.4)	16 (± 3.7)	14 (± 4.0)
**Aß positive**	37 (19.3%)	47 (51.6%)	58 (62.4%)
**p-tau217 Simoa**	0.40 (± 0.45)	0.64 (± 0.55)	1.1 (± 0.86)

VALIANT cohort			
	**CU (N=305)**	**MCI (N=51)**	**Dem (N=20)**
**Sex (Male)**	125 (41.0%)	14 (27.5%)	7 (35.0%)
**Age**	67 (± 11)	75 (± 9.1)	79 (± 10)
**Years of education**	5.8 (± 4.6)	2.3 (± 3.3)	2.4 (± 4.2)
**Aß positive**	62 (20.3%)	19 (37.3%)	10 (50.0%)
**p-tau217 Simoa**	0.28 (± 0.17)	0.38 (± 0.29)	0.42 (± 0.32)

Match 3. Cohorts were matched by age, sex and clinicopathological groups. N(CU)= 192, N(MCI)= 66, N(Dem)= 32 both for TRIAD and VALIANT cohorts.

Aß= amyloid; CU= cognitively unimpaired; Dem= dementia; MCI=mild cognitively impaired; N= number.

TRIAD cohort			
	**CU (N=192)**	**MCI (N=66)**	**Dem (N=32)**
**Sex (Male)**	129 (67.2%)	27 (40.9%)	9 (28.1%)
**Age**	69 (± 9.7)	71 (± 8.1)	73 (± 6.7)
**Years of education**	15 (± 3.4)	16 (± 3.6)	14 (± 4.2)
**Aß positive**	37 (19.3%)	22 (33.3%)	11 (34.4%)
**p-tau217 Simoa**	0.40 (± 0.45)	0.56 (± 0.56)	0.87 (± 0.89)

VALIANT cohort			
	**CU (N=192)**	**MCI (N=66)**	**Dem (N=32)**
**Sex (Male)**	101 (52.6%)	50 (75.8%)	24 (75.0%)
**Age**	67 (± 9.9)	72 (± 10)	77 (± 11)
**Years of education**	6.7 (± 4.8)	2.7 (± 3.7)	1.9 (± 3.7)
**Aß positive**	37 (19.3%)	22 (33.3%)	11 (34.4%)
**p-tau217 Simoa**	0.26 (± 0.15)	0.34 (± 0.27)	0.34 (± 0.27)

Supplementary Table 4. Comparison of plasma protein expression between amyloid-positive and amyloid-negative (first 50 results by Adj. P value). The table displays the linear regression results testing the association between amyloid-β status and each plasma protein. For each protein, the standardized beta values indicate the regression coefficient associated with amyloid-β positivity, adjusted for age, sex and average protein level. Proteins were classified as FDR-significant, nominally significant (P value) or not significant based on Benjamini–Hochberg-adjusted P values (Adj. P value) and nominal P values (P value). P values were derived from two-sided tests of the regression coefficient.

Adj. P value = Benjamini–Hochberg FDR-adjusted P value; B = regression coefficient (β estimate); Std. error = standard error of the regression coefficient; t = t statistic; logFC = log fold change; log10P = −log10(P value).

**Protein**	***B***	**Std.error**	***t***	***P* value**	**logFC**	**log10*P***	**Adj. *P* value**	**Significance**
p-tau231	0.4629	0.0529	8.7499	0.0000	0.4629	17.0081	1.21^{-15}	FDR
p-tau181	0.4434	0.0525	8.4448	0.0000	0.4434	15.9388	7.08^{-15}	FDR
p-tau217	0.2867	0.0440	6.5131	0.0000	0.2867	9.9198	4.93^{-9}	FDR
IL6R	-0.4954	0.1250	-3.9634	0.0001	-0.4954	4.0991	0.0021	FDR
TIMP3	-0.6535	0.1658	-3.9417	0.0001	-0.6535	4.0608	0.0021	FDR
MAPT	0.2696	0.0693	3.8924	0.0001	0.2696	3.9735	0.0022	FDR
FABP3	0.2134	0.0566	3.7683	0.0002	0.2134	3.7577	0.0031	FDR
KLK6	0.1547	0.0422	3.6642	0.0003	0.1547	3.5816	0.0040	FDR
IGF1R	0.2084	0.0577	3.6103	0.0003	0.2084	3.4920	0.0044	FDR
IL5	0.3743	0.1117	3.3519	0.0008	0.3743	3.0785	0.0103	FDR
HTT	-0.2835	0.0868	-3.2648	0.0011	-0.2835	2.9449	0.0127	FDR
CNTN2	-0.6097	0.1884	-3.2366	0.0013	-0.6097	2.9023	0.0128	FDR
CX3CL1	0.1319	0.0435	3.0356	0.0025	0.1319	2.6079	0.0233	FDR
CD40LG	-0.4262	0.1433	-2.9731	0.0030	-0.4262	2.5195	0.0266	FDR
ANXA5	-0.5508	0.1914	-2.8779	0.0041	-0.5508	2.3878	0.0302	FDR
VCAM1	0.1597	0.0549	2.9056	0.0038	0.1597	2.4257	0.0302	FDR
VGF	-0.5330	0.1856	-2.8719	0.0042	-0.5330	2.3796	0.0302	FDR
ENO2	-0.2849	0.1007	-2.8299	0.0048	-0.2849	2.3228	0.0325	FDR
BDNF	-0.3588	0.1295	-2.7705	0.0057	-0.3588	2.2435	0.0351	FDR
IL33	0.1959	0.0704	2.7818	0.0055	0.1959	2.2585	0.0351	FDR
PGK1	-0.3605	0.1394	-2.5869	0.0098	-0.3605	2.0072	0.0576	nominal
ARSA	-0.2115	0.0830	-2.5494	0.0110	-0.2115	1.9606	0.0612	nominal
FOLR1	0.2053	0.0816	2.5150	0.0121	0.2053	1.9183	0.0645	nominal
IGFBP7	0.0741	0.0301	2.4593	0.0141	0.0741	1.8507	0.0723	nominal
REST	0.1540	0.0647	2.3807	0.0175	0.1540	1.7574	0.0860	nominal
NGF	-0.1443	0.0613	-2.3526	0.0188	-0.1443	1.7247	0.0892	nominal
FCN2	-0.7124	0.3047	-2.3380	0.0196	-0.7124	1.7078	0.0893	nominal
CALB2	0.1677	0.0736	2.2795	0.0229	0.1677	1.6409	0.0950	nominal
CD63	0.0877	0.0385	2.2753	0.0231	0.0877	1.6361	0.0950	nominal

IL13	0.2961	0.1302	2.2744	0.0232	0.2961	1.6351	0.0950	nominal
GOT1	0.1038	0.0472	2.1996	0.0281	0.1038	1.5516	0.1114	nominal
ICAM1	0.0739	0.0343	2.1522	0.0316	0.0739	1.4997	0.1216	nominal
VEGFD	0.1909	0.0902	2.1164	0.0346	0.1909	1.4612	0.1289	nominal
CST3	0.0451	0.0216	2.0871	0.0372	0.0451	1.4300	0.1344	nominal
MSLN	0.1525	0.0745	2.0478	0.0409	0.1525	1.3887	0.1436	nominal
NRGN	-0.2775	0.1408	-1.9711	0.0490	-0.2775	1.3097	0.1654	nominal
S100B	0.1782	0.0913	1.9531	0.0511	0.1782	1.2915	0.1654	none
SLIT2	0.0702	0.0358	1.9614	0.0501	0.0702	1.2999	0.1654	none
NEFL	0.1941	0.1019	1.9046	0.0571	0.1941	1.2431	0.1802	none
GDI1	0.0884	0.0480	1.8412	0.0659	0.0884	1.1811	0.2027	none
CCL2	0.1664	0.0931	1.7871	0.0742	0.1664	1.1293	0.2225	none
IL4	0.1675	0.0943	1.7765	0.0760	0.1675	1.1193	0.2225	none
FLT1	-0.1019	0.0598	-1.7027	0.0890	-0.1019	1.0508	0.2487	none
NPY	-0.2125	0.1247	-1.7043	0.0887	-0.2125	1.0523	0.2487	none
ACHE	0.0672	0.0398	1.6908	0.0912	0.0672	1.0400	0.2493	none
HBA1	0.3418	0.2287	1.4946	0.1354	0.3418	0.8685	0.3619	none
TARDBP	-0.1056	0.0718	-1.4708	0.1417	-0.1056	0.8487	0.3708	none
MME	-0.2871	0.2008	-1.4301	0.1530	-0.2871	0.8152	0.3921	none
CSF2	0.1095	0.0777	1.4098	0.1589	0.1095	0.7988	0.3989	none
PTN	0.1645	0.1193	1.3789	0.1682	0.1645	0.7741	0.4139	none

Supplementary Table 5. Results from Gene Ontology for protein differentially expressed in amyloid-positive and amyloid-negative groups. For each term, the table lists: the group, the p-value corrected for multiple testing (Padj, FDR-corrected), the GO identifier (Term name); the specific genes from the query set that overlap with the GO term (Intersection), and the general Signature for each pathway.

Aβ-= amyloid-negative; Aβ+=amyloid-positive.

Group	Padj	term_id	Term name	Intersection	Signature
Aβ-	0.00035	GO:0005163	nerve growth factor receptor binding	BDNF, NGF	signaling receptor binding
Aβ-	0.00035	GO:0032813	tumor necrosis factor receptor superfamily binding	BDNF, CD40LG, NGF	signaling receptor binding
Aβ-	0.00107	GO:0005126	cytokine receptor binding	BDNF, CD40LG, IL6R, NGF	signaling receptor binding
Aβ-	0.00107	GO:0005165	neurotrophin receptor binding	BDNF, NGF	signaling receptor binding
Aβ-	0.00110	GO:0008191	metalloendopeptidase inhibitor activity	NGF, TIMP3	enzyme regulator activity
Aβ-	0.00191	GO:0005123	death receptor binding	BDNF, NGF	signaling receptor binding
Aβ-	0.00336	GO:0008083	growth factor activity	BDNF, NGF, VGF	signaling receptor binding
Aβ-	0.00540	GO:0005102	signaling receptor binding	BDNF, CD40LG, FCN2, IL6R, NGF, VGF	signaling receptor binding
Aβ+	0.00009	GO:0002269	leukocyte activation involved in inflammatory response	CX3CL1, IL13, IL33, MAPT	immune system process
Aβ+	0.00009	GO:0001774	microglial cell activation	CX3CL1, IL13, IL33, MAPT	immune system process
Aβ+	0.00010	GO:0061900	glial cell activation	CX3CL1, IL13, IL33, MAPT	immune system process
Aβ+	0.00016	GO:0071495	cellular response to endogenous stimulus	CD63, FOLR1, GOT1, IGF1R, IGFBP7, MAPT, REST, VCAM1, VEGFD	response to stimulus
Aβ+	0.00016	GO:0042127	regulation of cell population proliferation	CX3CL1, FABP3, IGF1R, IGFBP7, IL13, IL33, IL5, REST, VCAM1, VEGFD	response to stimulus
Aβ+	0.00018	GO:0150076	neuroinflammatory response	CX3CL1, IL13, IL33, MAPT	developmental process

Aβ+	0.00027	GO:0048869	cellular developmental process	CD63, CX3CL1, FABP3, FOLR1, ICAM1, IGF1R, IL13, IL33, IL5, KLK6, MAPT, REST, VCAM1, VEGFD	developmental process
Aβ+	0.00027	GO:0030154	cell differentiation	CD63, CX3CL1, FABP3, FOLR1, ICAM1, IGF1R, IL13, IL33, IL5, KLK6, MAPT, REST, VCAM1, VEGFD	cell differentiation

Supplementary Table 6. Linear regression analysis showing associations between pathologies and protein levels, adjusted for amyloid-status, age, sex, and average protein level. The table reports regression coefficients (β) with 95% confidence intervals (CIs), exact P values, and Benjamini–Hochberg FDR-adjusted q-values. Significance is indicated by * (nominal p<0.05) and ** (FDR q<0.05). Exact P values and FDR-adjusted q values are provided.

β = beta estimate; CI = confidence interval; FDR = false discovery rate; Sig. = significance.

**Protein**	**Exposure**	**Effect size (β)**	**CI (lower)**	**CI (higher)**	***P value***	**Q value**	**Sig.**	**Effect size (β) Group**	***P value* Group**
VGF	Cholesterol	0.1222	-0.2008	0.4452	0.4578	0.8394		-0.4967	0.0178
VEGFD	Cholesterol	0.0479	-0.1112	0.2069	0.5549	0.8482		0.2312	0.0237
VCAM1	Cholesterol	0.0344	-0.0692	0.1380	0.5146	0.8482		0.1478	0.0235
TIMP3	Cholesterol	-0.1115	-0.3950	0.1720	0.4403	0.8394		-0.6126	0.0008
SLIT2	Cholesterol	0.0199	-0.0431	0.0830	0.5354	0.8482		0.0509	0.2118
REST	Cholesterol	-0.0188	-0.1351	0.0976	0.7517	0.9608		0.1519	0.0403
pTau231	Cholesterol	-0.0692	-0.1620	0.0236	0.1434	0.6720		0.4187	0.0000
pTau217	Cholesterol	-0.0115	-0.0891	0.0662	0.7721	0.9608		0.2780	0.0000
pTau181	Cholesterol	-0.0972	-0.1883	-0.0060	0.0368	0.2756	*	0.3884	0.0000
PGK1	Cholesterol	-0.0007	-0.2443	0.2429	0.9958	0.9958		-0.2916	0.0656
NGF	Cholesterol	-0.0688	-0.1802	0.0427	0.2262	0.7409		-0.1742	0.0139
MAPT	Cholesterol	-0.0481	-0.1753	0.0791	0.4578	0.8394		0.1961	0.0155
KLK6	Cholesterol	-0.0498	-0.1231	0.0236	0.1833	0.6720		0.1328	0.0056
IL6R	Cholesterol	-0.0199	-0.2394	0.1997	0.8591	0.9642		-0.4907	0.0005

IL5	Cholesterol	-0.0139	-0.2044	0.1766	0.8862	0.9642		0.3347	0.0065
IL33	Cholesterol	-0.0176	-0.1469	0.1117	0.7890	0.9608		0.1657	0.0436
IL13	Cholesterol	0.0613	-0.1640	0.2865	0.5935	0.8482		0.2481	0.0862
IGFBP7	Cholesterol	0.0019	-0.0529	0.0567	0.9453	0.9748		0.0696	0.0471
IGF1R	Cholesterol	-0.0451	-0.1481	0.0579	0.3905	0.8394		0.1905	0.0047
HTT	Cholesterol	-0.1830	-0.3353	-0.0308	0.0185	0.2756	*	-0.2376	0.0152
FOLR1	Cholesterol	-0.1568	-0.3077	-0.0059	0.0418	0.2756	*	0.1648	0.0893
FCN2	Cholesterol	-0.1448	-0.6689	0.3794	0.5878	0.8482		-0.6803	0.0466
FABP3	Cholesterol	-0.1084	-0.2090	-0.0078	0.0348	0.2756	*	0.1744	0.0072
ENO2	Cholesterol	-0.1282	-0.3005	0.0440	0.1443	0.6720		-0.2670	0.0166
CX3CL1	Cholesterol	0.0438	-0.0320	0.1197	0.2568	0.7409		0.1458	0.0027
CST3	Cholesterol	0.0267	-0.0114	0.0647	0.1695	0.6720		0.0355	0.1447
CNTN2	Cholesterol	-0.1827	-0.5073	0.1418	0.2694	0.7409		-0.6241	0.0035
CD63	Cholesterol	-0.0040	-0.0707	0.0627	0.9057	0.9642		0.1063	0.0126
CD40LG	Cholesterol	-0.0647	-0.3187	0.1892	0.6169	0.8482		-0.4143	0.0116
CALB2	Cholesterol	0.0149	-0.1100	0.1397	0.8152	0.9608		0.1465	0.0714
BDNF	Cholesterol	-0.2296	-0.4484	-0.0109	0.0397	0.2756	*	-0.4040	0.0046
ARSA	Cholesterol	-0.0645	-0.2049	0.0759	0.3676	0.8394		-0.1823	0.0453
ANXA5	Cholesterol	-0.1525	-0.4858	0.1807	0.3692	0.8394		-0.4493	0.0379
VGF	Other conditions	-0.0127	-0.5502	0.5249	0.9631	0.9869		-0.4989	0.0175
VEGFD	Other conditions	0.0195	-0.2452	0.2842	0.8851	0.9869		0.2319	0.0235
VCAM1	Other conditions	0.0020	-0.1704	0.1744	0.9819	0.9869		0.1482	0.0234
TIMP3	Other conditions	-0.1391	-0.6110	0.3328	0.5629	0.9869		-0.6082	0.0009
SLIT2	Other conditions	0.0067	-0.0981	0.1116	0.8997	0.9869		0.0510	0.2111
REST	Other conditions	0.0108	-0.1828	0.2045	0.9126	0.9869		0.1494	0.0439
pTau231	Other conditions	0.0063	-0.1481	0.1607	0.9365	0.9869		0.4185	0.0000
pTau217	Other conditions	0.0366	-0.0926	0.1657	0.5782	0.9869		0.2776	0.0000
pTau181	Other conditions	-0.0073	-0.1591	0.1445	0.9245	0.9869		0.3877	0.0000
PGK1	Other conditions	-0.1042	-0.5097	0.3012	0.6138	0.9869		-0.2866	0.0705
NGF	Other conditions	0.0447	-0.1408	0.2302	0.6360	0.9869		-0.1740	0.0141

MAPT	Other conditions	-0.0334	-0.2451	0.1782	0.7566	0.9869		0.1951	0.0162
KLK6	Other conditions	-0.0035	-0.1256	0.1186	0.9554	0.9869		0.1328	0.0057
IL6R	Other conditions	0.2616	-0.1033	0.6265	0.1596	0.9869		-0.4937	0.0005
IL5	Other conditions	0.1773	-0.1398	0.4944	0.2726	0.9869		0.3306	0.0073
IL33	Other conditions	-0.0039	-0.2191	0.2112	0.9713	0.9869		0.1657	0.0440
IL13	Other conditions	0.0869	-0.2880	0.4619	0.6490	0.9869		0.2339	0.1020
IGFBP7	Other conditions	0.0734	-0.0177	0.1646	0.1143	0.9869		0.0678	0.0530
IGF1R	Other conditions	-0.1495	-0.3208	0.0219	0.0873	0.9869		0.1925	0.0043
HTT	Other conditions	-0.0419	-0.2953	0.2115	0.7455	0.9869		-0.2377	0.0153
FOLR1	Other conditions	-0.1182	-0.3693	0.1330	0.3559	0.9869		0.1665	0.0863
FCN2	Other conditions	0.1406	-0.7318	1.0130	0.7517	0.9869		-0.6759	0.0484
FABP3	Other conditions	0.1454	-0.0219	0.3128	0.0884	0.9869		0.1703	0.0085
ENO2	Other conditions	0.0024	-0.2843	0.2891	0.9869	0.9869		-0.2648	0.0177
CX3CL1	Other conditions	0.0155	-0.1107	0.1418	0.8094	0.9869		0.1456	0.0028
CST3	Other conditions	0.0223	-0.0410	0.0856	0.4896	0.9869		0.0348	0.1532
CNTN2	Other conditions	0.1260	-0.4141	0.6662	0.6470	0.9869		-0.6262	0.0035
CD63	Other conditions	0.0782	-0.0328	0.1892	0.1670	0.9869		0.1048	0.0140
CD40LG	Other conditions	0.1320	-0.2901	0.5541	0.5394	0.9869		-0.4136	0.0117
CALB2	Other conditions	0.0702	-0.1376	0.2780	0.5074	0.9869		0.1449	0.0749
BDNF	Other conditions	-0.0152	-0.3793	0.3489	0.9347	0.9869		-0.3993	0.0051
ARSA	Other conditions	0.1240	-0.1097	0.3576	0.2979	0.9869		-0.1828	0.0449
ANXA5	Other conditions	-0.0501	-0.6048	0.5045	0.8592	0.9869		-0.4432	0.0407
VGF	Heart diseases	0.0858	-0.2432	0.4149	0.6087	0.7794		-0.4704	0.0275
VEGFD	Heart diseases	-0.2021	-0.3642	-0.0401	0.0146	0.1840	*	0.2222	0.0320
VCAM1	Heart diseases	0.0253	-0.0802	0.1308	0.6377	0.7794		0.1422	0.0332
TIMP3	Heart diseases	0.1086	-0.1802	0.3975	0.4606	0.6908		-0.5860	0.0015
SLIT2	Heart diseases	-0.0331	-0.0974	0.0311	0.3115	0.5140		0.0467	0.2576
REST	Heart diseases	-0.0078	-0.1263	0.1108	0.8977	0.9257		0.1460	0.0533
pTau231	Heart diseases	0.0294	-0.0651	0.1239	0.5420	0.7777		0.4154	0.0000
pTau217	Heart diseases	0.0451	-0.0340	0.1242	0.2637	0.4591		0.2693	0.0000

pTau181	Heart diseases	0.0161	-0.0768	0.1090	0.7342	0.7837		0.3829	0.0000
PGK1	Heart diseases	-0.3282	-0.5764	-0.0801	0.0096	0.1840	*	-0.3152	0.0482
NGF	Heart diseases	-0.0968	-0.2103	0.0168	0.0948	0.2492		-0.1740	0.0156
MAPT	Heart diseases	0.0823	-0.0472	0.2119	0.2125	0.4125		0.1925	0.0194
KLK6	Heart diseases	0.0425	-0.0322	0.1173	0.2644	0.4591		0.1390	0.0042
IL6R	Heart diseases	-0.1917	-0.4145	0.0312	0.0918	0.2492		-0.4986	0.0005
IL5	Heart diseases	0.1837	-0.0104	0.3778	0.0636	0.2467		0.3579	0.0040
IL33	Heart diseases	-0.0054	-0.1371	0.1263	0.9359	0.9359		0.1647	0.0494
IL13	Heart diseases	0.1669	-0.0626	0.3964	0.1538	0.3383		0.2514	0.0839
IGFBP7	Heart diseases	-0.0113	-0.0672	0.0446	0.6912	0.7837		0.0695	0.0527
IGF1R	Heart diseases	0.0180	-0.0870	0.1230	0.7362	0.7837		0.1832	0.0074
HTT	Heart diseases	-0.1308	-0.2859	0.0243	0.0982	0.2492		-0.2346	0.0179
FOLR1	Heart diseases	-0.0395	-0.1932	0.1143	0.6146	0.7794		0.1609	0.1018
FCN2	Heart diseases	-0.3544	-0.8884	0.1796	0.1930	0.3981		-0.6298	0.0668
FABP3	Heart diseases	-0.0840	-0.1866	0.0185	0.1081	0.2547		0.1663	0.0116
ENO2	Heart diseases	-0.1525	-0.3280	0.0230	0.0885	0.2492		-0.2571	0.0226
CX3CL1	Heart diseases	-0.0721	-0.1494	0.0051	0.0673	0.2467		0.1324	0.0074
CST3	Heart diseases	-0.0418	-0.0805	-0.0030	0.0348	0.2074	*	0.0308	0.2124
CNTN2	Heart diseases	-0.1605	-0.4912	0.1701	0.3408	0.5355		-0.5334	0.0125
CD63	Heart diseases	0.0197	-0.0483	0.0877	0.5702	0.7794		0.1111	0.0106
CD40LG	Heart diseases	0.0574	-0.2012	0.3160	0.6633	0.7817		-0.3912	0.0186
CALB2	Heart diseases	-0.1338	-0.2610	-0.0066	0.0393	0.2074	*	0.1240	0.1282
BDNF	Heart diseases	0.2533	0.0304	0.4762	0.0260	0.2074	*	-0.3662	0.0106
ARSA	Heart diseases	-0.1747	-0.3178	-0.0317	0.0167	0.1840	*	-0.1927	0.0369
ANXA5	Heart diseases	-0.3489	-0.6885	-0.0094	0.0440	0.2074	*	-0.4634	0.0336
VGF	Infectious diseases	0.1507	-0.1953	0.4966	0.3928	0.5636		-0.4704	0.0275
VEGFD	Infectious diseases	0.1294	-0.0410	0.2998	0.1363	0.3460		-0.4786	0.0238
VCAM1	Infectious diseases	-0.0427	-0.1537	0.0682	0.4496	0.5935		0.2001	0.0556
TIMP3	Infectious diseases	0.1559	-0.1478	0.4596	0.3139	0.5115		0.1557	0.0208
SLIT2	Infectious diseases	-0.0726	-0.1402	-0.0051	0.0350	0.1292	*	-0.6204	0.0009

REST	Infectious diseases	-0.1350	-0.2596	-0.0103	0.0338	0.1292	*	0.0522	0.2084
pTau231	Infectious diseases	-0.0730	-0.1724	0.0263	0.1494	0.3522		0.1607	0.0347
pTau217	Infectious diseases	-0.0255	-0.1086	0.0577	0.5478	0.6456		0.4357	0.0000
pTau181	Infectious diseases	-0.0470	-0.1447	0.0507	0.3449	0.5173		0.2844	0.0000
PGK1	Infectious diseases	0.4130	0.1521	0.6739	0.0020	0.0324	**	0.4068	0.0000
NGF	Infectious diseases	0.0643	-0.0551	0.1838	0.2907	0.5115		-0.3646	0.0238
MAPT	Infectious diseases	-0.0194	-0.1556	0.1168	0.7801	0.8305		-0.1826	0.0124
KLK6	Infectious diseases	-0.1680	-0.2466	-0.0894	0.0000	0.0010	**	0.2070	0.0127
IL6R	Infectious diseases	0.2525	0.0175	0.4875	0.0352	0.1292	*	0.1415	0.0035
IL5	Infectious diseases	-0.1558	-0.3599	0.0483	0.1343	0.3460		-0.5375	0.0002
IL33	Infectious diseases	-0.0476	-0.1861	0.0909	0.4998	0.6344		0.3693	0.0035
IL13	Infectious diseases	-0.0191	-0.2604	0.2222	0.8766	0.9040		0.1711	0.0436
IGFBP7	Infectious diseases	-0.0373	-0.0961	0.0214	0.2123	0.4378		0.2485	0.0957
IGF1R	Infectious diseases	-0.1329	-0.2433	-0.0226	0.0183	0.1007	*	0.0636	0.0774
HTT	Infectious diseases	0.0817	-0.0813	0.2448	0.3255	0.5115		0.2071	0.0028
FOLR1	Infectious diseases	-0.0514	-0.2130	0.1103	0.5329	0.6456		-0.2575	0.0107
FCN2	Infectious diseases	0.7022	0.1408	1.2636	0.0143	0.0944	*	0.1576	0.1153
FABP3	Infectious diseases	-0.0749	-0.1827	0.0328	0.1726	0.3797		-0.7385	0.0337
ENO2	Infectious diseases	0.2427	0.0581	0.4272	0.0100	0.0827	*	0.1711	0.0097
CX3CL1	Infectious diseases	0.0339	-0.0473	0.1152	0.4128	0.5676		-0.2759	0.0154
CST3	Infectious diseases	0.0017	-0.0390	0.0425	0.9329	0.9329		0.1300	0.0091
CNTN2	Infectious diseases	0.2051	-0.1425	0.5527	0.2471	0.4796		0.0324	0.1943
CD63	Infectious diseases	-0.0183	-0.0898	0.0531	0.6145	0.6993		-0.6228	0.0043
CD40LG	Infectious diseases	0.1447	-0.1274	0.4168	0.2968	0.5115		0.1148	0.0087
CALB2	Infectious diseases	-0.1390	-0.2728	-0.0053	0.0417	0.1374	*	-0.4365	0.0095
BDNF	Infectious diseases	-0.0454	-0.2797	0.1890	0.7040	0.7744		0.1315	0.1121
ARSA	Infectious diseases	0.1154	-0.0350	0.2658	0.1324	0.3460		-0.3981	0.0061
ANXA5	Infectious diseases	0.4953	0.1383	0.8522	0.0066	0.0727	*	-0.1984	0.0325
VGF	Respiratory diseases	-0.4744	-1.1288	0.1800	0.1551	0.6698		-0.4886	0.0201
VEGFD	Respiratory diseases	-0.1307	-0.4530	0.1916	0.4263	0.9675		0.2367	0.0211

VCAM1	Respiratory diseases	-0.0166	-0.2264	0.1933	0.8768	0.9675		0.1486	0.0236
TIMP3	Respiratory diseases	0.5531	-0.0214	1.1276	0.0591	0.5585		-0.6267	0.0006
SLIT2	Respiratory diseases	0.0903	-0.0373	0.2180	0.1652	0.6698		0.0470	0.2500
REST	Respiratory diseases	0.0289	-0.2069	0.2647	0.8100	0.9675		0.1443	0.0522
pTau231	Respiratory diseases	0.0001	-0.1878	0.1881	0.9988	0.9988		0.4167	0.0000
pTau217	Respiratory diseases	-0.1068	-0.2640	0.0504	0.1827	0.6698		0.2814	0.0000
pTau181	Respiratory diseases	-0.0108	-0.1956	0.1740	0.9089	0.9675		0.3877	0.0000
PGK1	Respiratory diseases	-0.1158	-0.6094	0.3778	0.6453	0.9675		-0.2872	0.0712
NGF	Respiratory diseases	0.0058	-0.2200	0.2317	0.9595	0.9895		-0.1771	0.0127
MAPT	Respiratory diseases	-0.1569	-0.4146	0.1008	0.2325	0.7326		0.1976	0.0153
KLK6	Respiratory diseases	0.1267	-0.0219	0.2754	0.0946	0.6246		0.1304	0.0067
IL6R	Respiratory diseases	0.4439	0.0026	0.8852	0.0487	0.5585	*	-0.4996	0.0004
IL5	Respiratory diseases	0.0952	-0.2909	0.4812	0.6286	0.9675		0.3260	0.0080
IL33	Respiratory diseases	-0.0201	-0.2821	0.2418	0.8801	0.9675		0.1679	0.0422
IL13	Respiratory diseases	0.2229	-0.2335	0.6794	0.3379	0.8578		0.2280	0.1123
IGFBP7	Respiratory diseases	0.1189	0.0079	0.2299	0.0358	0.5585	*	0.0672	0.0562
IGF1R	Respiratory diseases	-0.0256	-0.2343	0.1830	0.8095	0.9675		0.1887	0.0053
HTT	Respiratory diseases	-0.0965	-0.4050	0.2119	0.5392	0.9675		-0.2300	0.0191
FOLR1	Respiratory diseases	-0.0481	-0.3539	0.2577	0.7576	0.9675		0.1639	0.0931
FCN2	Respiratory diseases	0.3950	-0.6671	1.4571	0.4655	0.9675		-0.6732	0.0497
FABP3	Respiratory diseases	0.1580	-0.0457	0.3618	0.1282	0.6698		0.1671	0.0102
ENO2	Respiratory diseases	-0.0827	-0.4318	0.2664	0.6419	0.9675		-0.2587	0.0209
CX3CL1	Respiratory diseases	0.0103	-0.1435	0.1640	0.8957	0.9675		0.1440	0.0032
CST3	Respiratory diseases	-0.0245	-0.1016	0.0526	0.5328	0.9675		0.0356	0.1436
CNTN2	Respiratory diseases	-0.1318	-0.7894	0.5259	0.6942	0.9675		-0.6223	0.0038
CD63	Respiratory diseases	0.0128	-0.1223	0.1479	0.8529	0.9675		0.1048	0.0143
CD40LG	Respiratory diseases	0.4790	-0.0349	0.9930	0.0677	0.5585		-0.4231	0.0099
CALB2	Respiratory diseases	0.0481	-0.2049	0.3011	0.7089	0.9675		0.1421	0.0814
BDNF	Respiratory diseases	0.2632	-0.1802	0.7065	0.2442	0.7326		-0.4054	0.0045
ARSA	Respiratory diseases	-0.1519	-0.4364	0.1325	0.2947	0.8104		-0.1729	0.0582

ANXA5	Respiratory diseases	-0.0398	-0.7151	0.6355	0.9079	0.9675		-0.4346	0.0454
VGF	Metabolic diseases	-0.0724	-0.8137	0.6689	0.8479	0.9027		-0.5344	0.0120
VEGFD	Metabolic diseases	0.0207	-0.3444	0.3858	0.9114	0.9260		0.2442	0.0191
VCAM1	Metabolic diseases	0.0589	-0.1788	0.2966	0.6269	0.9027		0.1339	0.0445
TIMP3	Metabolic diseases	-0.0308	-0.6816	0.6200	0.9260	0.9260		-0.5865	0.0015
SLIT2	Metabolic diseases	-0.0190	-0.1636	0.1255	0.7960	0.9027		0.0414	0.3186
REST	Metabolic diseases	0.2158	-0.0513	0.4829	0.1131	0.8179		0.1397	0.0634
pTau231	Metabolic diseases	0.1344	-0.0785	0.3473	0.2157	0.9027		0.3974	0.0000
pTau217	Metabolic diseases	-0.0228	-0.2009	0.1553	0.8019	0.9027		0.2731	0.0000
pTau181	Metabolic diseases	0.1780	-0.0314	0.3873	0.0955	0.8179		0.3662	0.0000
PGK1	Metabolic diseases	-0.0905	-0.6496	0.4687	0.7509	0.9027		-0.2325	0.1475
NGF	Metabolic diseases	-0.0812	-0.3370	0.1746	0.5332	0.9027		-0.1801	0.0125
MAPT	Metabolic diseases	0.1210	-0.1709	0.4129	0.4159	0.9027		0.1697	0.0399
KLK6	Metabolic diseases	0.1765	0.0081	0.3449	0.0400	0.6597	*	0.1299	0.0074
IL6R	Metabolic diseases	-0.2811	-0.7881	0.2260	0.2768	0.9027		-0.4772	0.0009
IL5	Metabolic diseases	0.0824	-0.3550	0.5197	0.7117	0.9027		0.3257	0.0088
IL33	Metabolic diseases	0.0501	-0.2466	0.3469	0.7402	0.9027		0.1391	0.0947
IL13	Metabolic diseases	0.1118	-0.4052	0.6289	0.6712	0.9027		0.2200	0.1314
IGFBP7	Metabolic diseases	0.0594	-0.0663	0.1852	0.3537	0.9027		0.0569	0.1105
IGF1R	Metabolic diseases	0.1432	-0.0931	0.3795	0.2346	0.9027		0.1788	0.0083
HTT	Metabolic diseases	-0.1952	-0.5446	0.1542	0.2732	0.9027		-0.2002	0.0432
FOLR1	Metabolic diseases	0.1098	-0.2366	0.4562	0.5338	0.9027		0.1418	0.1468
FCN2	Metabolic diseases	-0.9439	-2.1470	0.2592	0.1239	0.8179		-0.6676	0.0542
FABP3	Metabolic diseases	0.0742	-0.1566	0.3050	0.5280	0.9027		0.1474	0.0255
ENO2	Metabolic diseases	-0.1989	-0.5944	0.1965	0.3236	0.9027		-0.2552	0.0242
CX3CL1	Metabolic diseases	0.2266	0.0525	0.4007	0.0108	0.3576	*	0.1398	0.0047
CST3	Metabolic diseases	-0.0101	-0.0975	0.0772	0.8202	0.9027		0.0267	0.2814
CNTN2	Metabolic diseases	-0.4841	-1.2291	0.2609	0.2024	0.9027		-0.5765	0.0072
CD63	Metabolic diseases	0.0163	-0.1367	0.1694	0.8339	0.9027		0.0986	0.0234
CD40LG	Metabolic diseases	-0.1709	-0.7530	0.4113	0.5646	0.9027		-0.3817	0.0215

CALB2	Metabolic diseases	-0.0666	-0.3532	0.2200	0.6482	0.9027		0.1198	0.1437
BDNF	Metabolic diseases	0.2229	-0.2793	0.7250	0.3839	0.9027		-0.3835	0.0077
ARSA	Metabolic diseases	-0.0531	-0.3753	0.2692	0.7466	0.9027		-0.1407	0.1271
ANXA5	Metabolic diseases	-0.2817	-1.0466	0.4833	0.4699	0.9027		-0.3952	0.0712

Supplementary Table 7. **Comparison of plasma protein expression between amyloid-positive and amyloid-negative after adjusting for other pathologies (first 50 results by Adj. *P* value).** The table displays the linear regression results testing the association between amyloid-β status and each plasma protein, after adjusting for other pathologies. For each protein, the standardized beta values indicate the regression coefficient associated with amyloid-β positivity, adjusted for age, sex, average protein level and other pathologies. Proteins were classified as FDR-significant, nominally significant (P value) or not significant based on Benjamini–Hochberg-adjusted P values (Adj. P value) and nominal P values (P value). P values were derived from two-sided tests of the regression coefficient.

Adj. P value = Benjamini–Hochberg FDR-adjusted P value; B = regression coefficient (β estimate); Std. error = standard error of the regression coefficient; t = t statistic; logFC = log fold change; log10P = −log10(P value).

**Protein**	**B**	**Std.error**	**t**	**P value**	**logFC**	**log10P**	**Adj. P value**	**Significance**
p-tau231	0.4133	0.0621	6.6548	0.0000	0.4133	10.2347	0.0000	FDR
p-tau181	0.3825	0.0611	6.2649	0.0000	0.3825	9.1805	0.0000	FDR
p-tau217	0.2738	0.0522	5.2466	0.0000	0.2738	6.6838	0.0000	FDR
IL6R	-0.5338	0.1460	-3.6559	0.0003	-0.5338	3.5586	0.0085	FDR
TIMP3	-0.5956	0.1898	-3.1376	0.0018	-0.5956	2.7504	0.0437	FDR
KLK6	0.1419	0.0491	2.8882	0.0040	0.1419	2.3982	0.0820	nominal
IL5	0.3598	0.1276	2.8204	0.0049	0.3598	2.3067	0.0867	nominal
IGF1R	0.1880	0.0693	2.7143	0.0068	0.1880	2.1669	0.1047	nominal
NGF	-0.1915	0.0746	-2.5656	0.0105	-0.1915	1.9783	0.1437	nominal
CD40LG	-0.4189	0.1699	-2.4656	0.0139	-0.4189	1.8562	0.1483	nominal
CD63	0.1111	0.0448	2.4779	0.0135	0.1111	1.8711	0.1483	nominal
CNTN2	-0.5328	0.2173	-2.4517	0.0145	-0.5328	1.8396	0.1483	nominal
BDNF	-0.3494	0.1465	-2.3849	0.0174	-0.3494	1.7606	0.1525	nominal

CX3CL1	0.1221	0.0508	2.4035	0.0165	0.1221	1.7824	0.1525	nominal
ENO2	-0.2586	0.1154	-2.2421	0.0253	-0.2586	1.5973	0.1829	nominal
S100B	0.2368	0.1055	2.2441	0.0251	0.2368	1.5995	0.1829	nominal
VGF	-0.4924	0.2162	-2.2770	0.0231	-0.4924	1.6365	0.1829	nominal
FABP3	0.1480	0.0676	2.1885	0.0290	0.1480	1.5381	0.1980	nominal
ANXA5	-0.4724	0.2231	-2.1170	0.0346	-0.4724	1.4607	0.2122	nominal
HTT	-0.2139	0.1019	-2.0985	0.0362	-0.2139	1.4410	0.2122	nominal
MAPT	0.1820	0.0852	2.1370	0.0329	0.1820	1.4822	0.2122	nominal
CCL13	-0.1663	0.0847	-1.9646	0.0499	-0.1663	1.3022	0.2361	nominal
FCN2	-0.6794	0.3509	-1.9359	0.0533	-0.6794	1.2734	0.2361	none
GOT1	0.1122	0.0567	1.9781	0.0483	0.1122	1.3159	0.2361	nominal
HBA1	0.4981	0.2585	1.9272	0.0544	0.4981	1.2646	0.2361	none
IL4	0.2169	0.1142	1.8987	0.0580	0.2169	1.2364	0.2361	none
NRGN	-0.3065	0.1601	-1.9152	0.0559	-0.3065	1.2527	0.2361	none
REST	0.1471	0.0779	1.8876	0.0595	0.1471	1.2255	0.2361	none
SMOC1	-0.2891	0.1511	-1.9130	0.0562	-0.2891	1.2506	0.2361	none
VCAM1	0.1382	0.0693	1.9928	0.0467	0.1382	1.3308	0.2361	nominal
VEGFD	0.2086	0.1065	1.9590	0.0505	0.2086	1.2965	0.2361	none
PGK1	-0.3020	0.1631	-1.8513	0.0646	-0.3020	1.1901	0.2481	none
ARSA	-0.1675	0.0940	-1.7816	0.0753	-0.1675	1.1234	0.2805	none
CSF2	0.1499	0.0858	1.7465	0.0812	0.1499	1.0906	0.2936	none
IL33	0.1493	0.0866	1.7251	0.0850	0.1493	1.0707	0.2986	none
ICAM1	0.0664	0.0390	1.7034	0.0890	0.0664	1.0508	0.3039	none
AB40	-0.1950	0.1206	-1.6176	0.1062	-0.1950	0.9739	0.3531	none
MSLN	0.1336	0.0867	1.5411	0.1238	0.1336	0.9074	0.4006	none
FLT1	-0.1085	0.0725	-1.4971	0.1348	-0.1085	0.8702	0.4045	none
IGFBP7	0.0554	0.0369	1.5040	0.1330	0.0554	0.8760	0.4045	none
IL13	0.2267	0.1508	1.5030	0.1333	0.2267	0.8751	0.4045	none
IL12p70	0.1460	0.0992	1.4722	0.1414	0.1460	0.8495	0.4045	none
NPY	-0.2205	0.1485	-1.4843	0.1382	-0.2205	0.8595	0.4045	none

CCL3	-0.2760	0.1992	-1.3858	0.1663	-0.2760	0.7792	0.4648	none
ACHE	0.0626	0.0468	1.3363	0.1819	0.0626	0.7402	0.4971	none
AB42	-0.1253	0.0965	-1.2990	0.1944	-0.1253	0.7113	0.5147	none
FOLR1	0.1306	0.1010	1.2924	0.1967	0.1306	0.7063	0.5147	none
IL2	-0.1163	0.0917	-1.2678	0.2053	-0.1163	0.6876	0.5261	none
CALB2	0.0973	0.0836	1.1644	0.2447	0.0973	0.6114	0.5666	none
CCL2	0.1363	0.1154	1.1805	0.2382	0.1363	0.6230	0.5666	none

Supplementary Table 8. Comparison of plasma protein expression between amyloid-positive and amyloid-negative with amyloid positivity defined as values exceeding the 70th percentile of p-tau217 measured with NULISA (first 50 results by Adj. P value). The table displays the linear regression results testing the association between amyloid-β status and each plasma protein. For each protein, the standardized beta values indicate the regression coefficient associated with amyloid-β positivity, adjusted for age, sex and average protein level. Proteins were classified as FDR-significant, nominally significant (P value) or not significant based on Benjamini–Hochberg-adjusted P values (Adj. P value) and nominal P values (P value). P values were derived from two-sided tests of the regression coefficient.

Adj. P value = Benjamini–Hochberg FDR-adjusted P value; B = regression coefficient (β estimate); Std. error = standard error of the regression coefficient; t = t statistic; logFC = log fold change; log10P = −log10(P value).

**Protein**	**B**	**Std.error**	**t**	**P value**	**logFC**	**log10P**	**Adj. P value**	**Significance**
p-tau217	0.7417	0.02733	27.1424	6E-120	0.7417	119.2570	6.81E-118	FDR
p-tau231	0.6493	0.03960	16.3955	3E-53	0.6493	52.5339	1.80E-51	FDR
p-tau181	0.5946	0.03994	14.8873	4E-45	0.5946	44.3976	1.64E-43	FDR
MAPT	0.5479	0.05421	10.1084	7E-23	0.5479	22.1347	2.25E-21	FDR
SNAP25	0.1203	0.01425	8.4459	1E-16	0.1203	15.9399	2.83E-15	FDR
POSTN	0.3764	0.05891	6.3891	3E-10	0.3764	9.5794	5.40E-09	FDR
KLK6	0.2114	0.03399	6.2185	8E-10	0.2114	9.1209	1.33E-08	FDR
FABP3	0.2794	0.04552	6.1377	1E-09	0.2794	8.9072	1.90E-08	FDR
GDI1	0.2326	0.03847	6.0475	2E-09	0.2326	8.6720	2.91E-08	FDR
CD40LG	-0.6733	0.11581	-5.8142	8E-09	-0.6733	8.0768	1.03E-07	FDR

HTT	-0.3995	0.07013	-5.6975	2E-08	-0.3995	7.7875	1.82E-07	FDR
IL33	0.3102	0.05687	5.4545	6E-08	0.3102	7.2009	6.10E-07	FDR
NPY	0.5465	0.10028	5.4500	6E-08	0.5465	7.1904	6.10E-07	FDR
S100A12	-0.5228	0.09803	-5.3326	1E-07	-0.5228	6.9152	1.07E-06	FDR
BDNF	-0.5435	0.10463	-5.1943	3E-07	-0.5435	6.5977	1.96E-06	FDR
IGFBP7	0.1260	0.02427	5.1929	3E-07	0.1260	6.5945	1.96E-06	FDR
PGK1	-0.5759	0.11269	-5.1100	4E-07	-0.5759	6.4078	2.83E-06	FDR
SQSTM1	0.2346	0.04663	5.0321	6E-07	0.2346	6.2349	3.98E-06	FDR
pTDP43409	0.1984	0.04009	4.9490	9E-07	0.1984	6.0530	5.73E-06	FDR
CST3	0.0839	0.01745	4.8081	2E-06	0.0839	5.7504	1.09E-05	FDR
IL6R	-0.4888	0.10232	-4.7766	2E-06	-0.4888	5.6829	1.22E-05	FDR
CCL2	-0.3529	0.07535	-4.6837	3E-06	-0.3529	5.4896	1.76E-05	FDR
NGF	-0.2326	0.04970	-4.6804	3E-06	-0.2326	5.4828	1.76E-05	FDR
AB42	0.3025	0.06632	4.5616	6E-06	0.3025	5.2399	2.95E-05	FDR
CD63	0.1397	0.03098	4.5105	7E-06	0.1397	5.1370	3.59E-05	FDR
GOT1	0.1624	0.03809	4.2645	2E-05	0.1624	4.6560	1.04E-04	FDR
TIMP3	-0.5699	0.13511	-4.2179	3E-05	-0.5699	4.5676	1.23E-04	FDR
MDH1	-0.2254	0.05539	-4.0691	5E-05	-0.2254	4.2905	2.25E-04	FDR
IGF1R	0.1879	0.04687	4.0084	7E-05	0.1879	4.1804	2.80E-04	FDR
MSLN	0.2329	0.06037	3.8581	1E-04	0.2329	3.9131	5.01E-04	FDR
ANXA5	-0.5929	0.15541	-3.8152	1E-04	-0.5929	3.8385	5.75E-04	FDR
MME	-0.6078	0.16209	-3.7494	2E-04	-0.6078	3.7253	7.23E-04	FDR
ARSA	-0.2501	0.06747	-3.7066	2E-04	-0.2501	3.6527	8.29E-04	FDR
TEK	-0.1344	0.03948	-3.4040	7E-04	-0.1344	3.1595	2.51E-03	FDR
IL2	-0.2153	0.06388	-3.3709	8E-04	-0.2153	3.1078	2.74E-03	FDR
CNTN2	-0.4986	0.15326	-3.2531	1E-03	-0.4986	2.9271	4.04E-03	FDR
CALB2	0.1857	0.05981	3.1054	2E-03	0.1857	2.7083	6.51E-03	FDR
CXCL1	-0.3286	0.11240	-2.9235	4E-03	-0.3286	2.4503	1.13E-02	FDR
CHI3L1	0.3172	0.10893	2.9124	4E-03	0.3172	2.4350	1.13E-02	FDR
NPTX2	0.1107	0.03798	2.9141	4E-03	0.1107	2.4374	1.13E-02	FDR

pSNCA129	-0.2145	0.07421	-2.8902	4E-03	-0.2145	2.4046	1.18E-02	FDR
SNCB	0.1068	0.03709	2.8798	4E-03	0.1068	2.3904	1.19E-02	FDR
CCL13	-0.1710	0.05996	-2.8515	4E-03	-0.1710	2.3519	1.27E-02	FDR
SMOC1	0.2918	0.10373	2.8131	5E-03	0.2918	2.3001	1.40E-02	FDR
NEFL	0.2286	0.08305	2.7528	6E-03	0.2286	2.2202	1.65E-02	FDR
REST	0.1427	0.05276	2.7038	7E-03	0.1427	2.1562	1.87E-02	FDR
TNF	-0.1111	0.04162	-2.6706	8E-03	-0.1111	2.1133	2.02E-02	FDR
NEFH	0.6835	0.26004	2.6283	9E-03	0.6835	2.0594	2.23E-02	FDR
IL7	-0.2133	0.08533	-2.4999	1E-02	-0.2133	1.8998	3.16E-02	FDR

Supplementary Table 9. Comparison of plasma protein expression between amyloid-positive and amyloid-negative in the Tanzanian cohort, with amyloid positivity defined as values exceeding the 70th percentile of p-tau217 measured with NULISA (first 50 results by Adj. P value). The table displays the linear regression results testing the association between amyloid-β status and each plasma protein. For each protein, the standardized beta values indicate the regression coefficient associated with amyloid-β positivity, adjusted for age, sex and average protein level. Proteins were classified as FDR-significant, nominally significant (P value) or not significant based on Benjamini–Hochberg-adjusted P values (Adj. P value) and nominal P values (P value). P values were derived from two-sided tests of the regression coefficient.

Adj. P value = Benjamini–Hochberg FDR-adjusted P value; B = regression coefficient (β estimate); Std. error = standard error of the regression coefficient; t = t statistic; logFC = log fold change; log10P = −log10(P value).

Protein	B	Std.error	t	P value	logFC	log10P	Adj. P value	Significance
p-tau217	0.8607	0.0479	17.9647	0.0000	0.8607	42.1761	0.0000	FDR
p-tau231	1.3817	0.1105	12.5032	0.0000	1.3817	25.8706	0.0000	FDR
p-tau181	1.1556	0.0978	11.8163	0.0000	1.1556	23.8127	0.0000	FDR
MAPT	1.1439	0.1232	9.2881	0.0000	1.1439	16.4488	0.0000	FDR
PARK7	-0.5898	0.1173	-5.0303	0.0000	-0.5898	5.9488	0.0000	FDR
IL16	-0.3439	0.0761	-4.5176	0.0000	-0.3439	4.9609	0.0002	FDR
POSTN	1.4651	0.3340	4.3862	0.0000	1.4651	4.7203	0.0003	FDR
PGK1	-0.6663	0.1623	-4.1043	0.0001	-0.6663	4.2215	0.0008	FDR

IGFBP7	0.1975	0.0478	4.1327	0.0001	0.1975	4.2706	0.0008	FDR
GFAP	0.4148	0.1078	3.8465	0.0002	0.4148	3.7872	0.0020	FDR
FABP3	0.3531	0.0933	3.7853	0.0002	0.3531	3.6873	0.0023	FDR
CXCL8	-1.1314	0.3123	-3.6234	0.0004	-1.1314	3.4287	0.0036	FDR
CX3CL1	0.3019	0.0831	3.6312	0.0004	0.3019	3.4409	0.0036	FDR
NEFL	0.4170	0.1215	3.4308	0.0007	0.4170	3.1322	0.0065	FDR
NPTX2	0.2435	0.0745	3.2690	0.0013	0.2435	2.8927	0.0106	FDR
IL6	-1.0002	0.3170	-3.1555	0.0019	-1.0002	2.7301	0.0137	FDR
TARDBP	-0.3529	0.1120	-3.1518	0.0019	-0.3529	2.7249	0.0137	FDR
RUVBL2	-0.2686	0.0876	-3.0675	0.0025	-0.2686	2.6069	0.0170	FDR
SOD1	-0.3020	0.1014	-2.9782	0.0033	-0.3020	2.4848	0.0214	FDR
FOLR1	0.1988	0.0677	2.9369	0.0037	0.1988	2.4291	0.0231	FDR
CCL3	-0.8941	0.3109	-2.8759	0.0045	-0.8941	2.3481	0.0265	FDR
CRP	-0.7196	0.2531	-2.8427	0.0050	-0.7196	2.3046	0.0280	FDR
IGF1R	0.0953	0.0343	2.7814	0.0060	0.0953	2.2251	0.0308	FDR
SMOC1	0.5319	0.1903	2.7950	0.0057	0.5319	2.2426	0.0308	FDR
NRGN	0.4722	0.1749	2.6996	0.0076	0.4722	2.1211	0.0375	FDR
GDF15	0.4448	0.1669	2.6653	0.0084	0.4448	2.0783	0.0398	FDR
YWHAZ	-0.2460	0.0929	-2.6466	0.0088	-0.2460	2.0551	0.0401	FDR
ACHE	0.1664	0.0634	2.6249	0.0094	0.1664	2.0283	0.0401	FDR
CXCL10	0.3795	0.1447	2.6225	0.0094	0.3795	2.0254	0.0401	FDR
SLIT2	0.2407	0.0921	2.6127	0.0097	0.2407	2.0133	0.0401	FDR
S100A12	-0.3716	0.1431	-2.5961	0.0102	-0.3716	1.9930	0.0407	FDR
IL1B	-1.1638	0.4664	-2.4952	0.0134	-1.1638	1.8718	0.0517	nominal
TAFA5	0.6217	0.2501	2.4863	0.0138	0.6217	1.8612	0.0517	nominal
TNF	-0.5135	0.2093	-2.4533	0.0151	-0.5135	1.8224	0.0533	nominal
AB42	0.0631	0.0257	2.4550	0.0150	0.0631	1.8244	0.0533	nominal
SNCB	0.1844	0.0760	2.4268	0.0162	0.1844	1.7916	0.0557	nominal
CCL11	0.4022	0.1688	2.3821	0.0182	0.4022	1.7400	0.0596	nominal
CST3	0.1431	0.0601	2.3808	0.0183	0.1431	1.7385	0.0596	nominal

CCL22	-0.2039	0.0882	-2.3121	0.0218	-0.2039	1.6608	0.0694	nominal
HTT	-0.2250	0.1008	-2.2319	0.0268	-0.2250	1.5722	0.0830	nominal
IL5	-0.5207	0.2433	-2.1397	0.0336	-0.5207	1.4731	0.1018	nominal
AGRN	0.1040	0.0505	2.0614	0.0406	0.1040	1.3913	0.1199	nominal
MSLN	0.2399	0.1195	2.0084	0.0460	0.2399	1.3371	0.1327	nominal
CNTN2	0.3015	0.1511	1.9961	0.0473	0.3015	1.3247	0.1334	nominal
MME	-0.5567	0.2847	-1.9553	0.0520	-0.5567	1.2839	0.1401	none
IL15	0.1140	0.0584	1.9518	0.0524	0.1140	1.2805	0.1401	none
NEFH	0.7215	0.3707	1.9462	0.0531	0.7215	1.2749	0.1401	none
KLK6	0.1570	0.0825	1.9027	0.0586	0.1570	1.2322	0.1513	none
IL4	-0.3022	0.1651	-1.8300	0.0688	-0.3022	1.1624	0.1741	none

Supplementary Table 10. Comparison of plasma protein expression between CU and CI in amyloid-positive participants (first 50 results by Adj. P value). The table displays the linear regression results testing the association between cognitive status (in amyloid-positive participants) and each plasma protein. For each protein, the standardized beta values indicate the regression coefficient associated with cognitive impairment, adjusted for age, sex and average protein level. Proteins were classified as FDR-significant, nominally significant (P value) or not significant based on Benjamini–Hochberg-adjusted P values (Adj. P value) and nominal P values (P value). P values were derived from two-sided tests of the regression coefficient.

Adj. P value = Benjamini–Hochberg FDR-adjusted P value; B = regression coefficient (β estimate); Std. error = standard error of the regression coefficient; t = t statistic; logFC = log fold change; log10P = −log10(P value).

**Protein**	**B**	**Std.error**	**t**	**P value**	**logFC**	**log10P**	**Adj. P value**	**Significance**
FCN2	-1.7848	0.8424	-2.1186	0.0358	-1.7848	1.4459	0.5062	nominal
GDI1	0.2759	0.1318	2.0926	0.0381	0.2759	1.4189	0.5062	nominal
KLK6	0.2219	0.1072	2.0687	0.0403	0.2219	1.3943	0.5062	nominal
MME	-1.1005	0.4875	-2.2576	0.0255	-1.1005	1.5942	0.5062	nominal
NPTX1	0.3334	0.1328	2.5112	0.0131	0.3334	1.8821	0.5062	nominal
p-tau217	0.2527	0.1226	2.0604	0.0412	0.2527	1.3856	0.5062	nominal
TAFA5	0.5963	0.2621	2.2753	0.0243	0.5963	1.6136	0.5062	nominal

TIMP3	-0.8629	0.4102	-2.1034	0.0371	-0.8629	1.4301	0.5062	nominal
TNF	-0.2352	0.1075	-2.1892	0.0302	-0.2352	1.5205	0.5062	nominal
UCHL1	0.1080	0.0518	2.0830	0.0390	0.1080	1.4090	0.5062	nominal
CCL11	0.3343	0.1678	1.9919	0.0482	0.3343	1.3166	0.5395	nominal
IGFBP7	0.1291	0.0683	1.8907	0.0607	0.1291	1.2171	0.5831	none
NPTX2	0.2020	0.1073	1.8835	0.0616	0.2020	1.2102	0.5831	none
APOE	-0.2108	0.1387	-1.5199	0.1307	-0.2108	0.8837	0.6568	none
AB42	-0.3448	0.2000	-1.7244	0.0868	-0.3448	1.0617	0.6568	none
BDNF	-0.5340	0.3322	-1.6076	0.1101	-0.5340	0.9583	0.6568	none
FLT1	-0.2646	0.1768	-1.4967	0.1366	-0.2646	0.8645	0.6568	none
GDNF	0.7452	0.4230	1.7616	0.0802	0.7452	1.0956	0.6568	none
GFAP	0.3383	0.2219	1.5247	0.1295	0.3383	0.8878	0.6568	none
IGF1R	0.2668	0.1597	1.6702	0.0970	0.2668	1.0131	0.6568	none
IL18	0.2186	0.1377	1.5868	0.1147	0.2186	0.9404	0.6568	none
NPTXR	0.1727	0.1071	1.6121	0.1091	0.1727	0.9621	0.6568	none
POSTN	0.3424	0.2319	1.4764	0.1420	0.3424	0.8477	0.6568	none
p-tau231	0.3164	0.2076	1.5237	0.1297	0.3164	0.8869	0.6568	none
RUVBL2	0.2264	0.1542	1.4683	0.1442	0.2264	0.8411	0.6568	none
SNAP25	0.0765	0.0482	1.5880	0.1144	0.0765	0.9414	0.6568	none
SOD1	0.3632	0.2211	1.6428	0.1026	0.3632	0.9890	0.6568	none
MAPT	0.2819	0.1985	1.4196	0.1578	0.2819	0.8018	0.6580	none
MDH1	0.2606	0.1824	1.4290	0.1551	0.2606	0.8093	0.6580	none
SAA1	-0.7263	0.5149	-1.4106	0.1605	-0.7263	0.7945	0.6580	none
IL33	0.2185	0.1576	1.3870	0.1676	0.2185	0.7758	0.6649	none
CCL13	-0.2556	0.1940	-1.3176	0.1897	-0.2556	0.7219	0.6805	none
FOLR1	0.2336	0.1785	1.3086	0.1927	0.2336	0.7150	0.6805	none
OligoSNCA	0.5228	0.3865	1.3526	0.1783	0.5228	0.7489	0.6805	none
pSNCA129	0.2947	0.2257	1.3059	0.1936	0.2947	0.7130	0.6805	none
S100A12	-0.4074	0.3275	-1.2439	0.2155	-0.4074	0.6665	0.7364	none
ANXA5	-0.6419	0.5400	-1.1887	0.2365	-0.6419	0.6262	0.7401	none

CRP	-0.4588	0.3929	-1.1677	0.2448	-0.4588	0.6112	0.7401	none
IFNG	-0.3754	0.3228	-1.1631	0.2467	-0.3754	0.6078	0.7401	none
IL6R	-0.4485	0.3663	-1.2244	0.2228	-0.4485	0.6521	0.7401	none
p-tau181	0.2362	0.1980	1.1929	0.2349	0.2362	0.6292	0.7401	none
IL15	-0.1327	0.1163	-1.1408	0.2558	-0.1327	0.5921	0.7492	none
CD40LG	-0.4217	0.4005	-1.0530	0.2941	-0.4217	0.5315	0.7863	none
CST3	0.0520	0.0493	1.0554	0.2930	0.0520	0.5331	0.7863	none
IL6	-0.2743	0.2640	-1.0391	0.3005	-0.2743	0.5222	0.7863	none
NEFH	0.8487	0.7938	1.0692	0.2867	0.8487	0.5425	0.7863	none
SLIT2	0.0980	0.0909	1.0775	0.2831	0.0980	0.5481	0.7863	none
CCL17	-0.3330	0.3703	-0.8992	0.3700	-0.3330	0.4318	0.7926	none
CCL2	0.1785	0.1855	0.9624	0.3375	0.1785	0.4718	0.7926	none
CHIT1	0.5356	0.5847	0.9161	0.3612	0.5356	0.4423	0.7926	none

Supplementary Table 11. Results from Gene Ontology for protein differentially expressed in CU and CI in amyloid-positive groups. For each term, the table lists: the group, the p-value corrected for multiple testing (Padj, FDR-corrected), the GO identifier (Term name); the specific genes from the query set that overlap with the GO term (Intersection), and the general Signature for each pathway.

CU= cognitively unimpaired; CI= cognitively impaired; Aβ-= amyloid-negative; Aβ+=amyloid-positive.

**Group**	**Padj**	**term_id**	**Term name**	**Intersection**	**Signature**
CU Aβ+	0.0067	GO:0070012	oligopeptidase activity	MME	catalytic activity
CU Aβ+	0.0067	GO:0004222	metalloendopeptidase activity	MME, TIMP3	catalytic activity
CU Aβ+	0.0067	GO:0038177	death receptor agonist activity	TNF	signaling receptor binding
CU Aβ+	0.0067	GO:2001065	mannan binding	FCN2	carbohydrate binding

CU Aβ+	0.0067	GO:0002020	protease binding	TIMP3, TNF	protein metabolic process
CU Aβ+	0.0067	GO:0008237	metallopeptidase activity	MME, TIMP3	catalytic activity
CU Aβ+	0.0081	GO:0097242	amyloid-beta clearance	MME, TNF	protein metabolic process
CU Aβ+	0.0081	GO:0006508	proteolysis	FCN2, MME, TIMP3, TNF	protein metabolic process
CI Aβ+	0.0012	GO:0007409	axonogenesis	GDI1, NPTX1, MAPT, UCHL1	developmental process
CI Aβ+	0.0012	GO:0048666	neuron development	GDI1, KLK6, NPTX1, MAPT, UCHL1	developmental process
CI Aβ+	0.0012	GO:0000902	cell morphogenesis	CCL11, GDI1, NPTX1, MAPT, UCHL1	developmental process
CI Aβ+	0.0012	GO:0031175	neuron projection development	GDI1, KLK6, NPTX1, MAPT, UCHL1	cellular component organization
CI Aβ+	0.0012	GO:0022008	neurogenesis	CCL11, GDI1, KLK6, NPTX1, MAPT, UCHL1	cell differentiation
CI Aβ+	0.0012	GO:0048731	system development	CCL11, GDI1, KLK6, NPTX1, MAPT, TAFA5, UCHL1	developmental process
CI Aβ+	0.0013	GO:0061564	axon development	GDI1, NPTX1, MAPT, UCHL1	cellular component organization
CI Aβ+	0.0013	GO:0019896	axonal transport of mitochondrion	MAPT, UCHL1	establishment of localization

Supplementary Table 12. Linear regression analysis showing associations between pathologies and protein levels, adjusted for cognitive status, age, sex and average protein level. The table reports regression coefficients (β) with 95% confidence intervals

(CIs), exact P values, and Benjamini–Hochberg FDR-adjusted q-values. Significance is indicated by * (nominal p<0.05) and ** (FDR q<0.05). Exact P values and FDR-adjusted q values are provided.

β = beta estimate; CI = confidence interval; FDR = false discovery rate; Sig. = significance.

**Protein**	**Exposure**	**Effect size (β)**	**CI (lower)**	**CI (higher)**	***P value***	**Q value**	**Sig.**	**Effect size (β) Group**	***P value* Group**
UCHL1	Cholesterol	-0.0901	-0.1841	0.0039	0.0601	0.6615		0.1518	0.0094
TNF	Cholesterol	0.0395	-0.1428	0.2218	0.6681	0.9509		-0.2335	0.0486
TIMP3	Cholesterol	-0.1110	-0.7977	0.5757	0.7493	0.9509		-0.9266	0.0408
TAFA5	Cholesterol	0.1462	-0.3093	0.6017	0.5259	0.9509		0.5688	0.0560
p-tau217	Cholesterol	-0.0723	-0.2806	0.1360	0.4930	0.9509		0.3806	0.0047
NPTX1	Cholesterol	-0.1798	-0.4193	0.0597	0.1396	0.7678		0.3336	0.0271
MME	Cholesterol	0.1743	-0.6719	1.0205	0.6838	0.9509		-1.1470	0.0369
KLK6	Cholesterol	-0.1063	-0.2950	0.0823	0.2664	0.9509		0.2054	0.0903
GDI1	Cholesterol	0.0324	-0.1951	0.2600	0.7780	0.9509		0.2834	0.0560
FCN2	Cholesterol	-0.0201	-1.4318	1.3916	0.9776	0.9776		-2.0199	0.0326
CCL11	Cholesterol	-0.0142	-0.3023	0.2740	0.9226	0.9776		0.3873	0.0344
UCHL1	Other conditions	0.0259	-0.1294	0.1812	0.7414	0.9441		0.1523	0.0101
TNF	Other conditions	0.3213	0.0201	0.6226	0.0368	0.4048	*	-0.2039	0.0832
TIMP3	Other conditions	0.1573	-0.9775	1.2920	0.7841	0.9441		-0.8789	0.0544
TAFA5	Other conditions	0.0579	-0.6948	0.8106	0.8790	0.9441		0.5880	0.0509
p-tau217	Other conditions	0.0187	-0.3239	0.3613	0.9139	0.9441		0.3862	0.0046
NPTX1	Other conditions	0.1959	-0.1998	0.5917	0.3286	0.9037		0.3544	0.0199
MME	Other conditions	0.3516	-1.0466	1.7499	0.6191	0.9441		-1.1439	0.0398
KLK6	Other conditions	-0.1985	-0.5102	0.1133	0.2097	0.7689		0.1886	0.1224
GDI1	Other conditions	-0.0285	-0.4044	0.3475	0.8810	0.9441		0.2776	0.0642
FCN2	Other conditions	1.7147	-0.6181	4.0475	0.1480	0.7689		-1.8348	0.0524
CCL11	Other conditions	0.0169	-0.4592	0.4930	0.9441	0.9441		0.3933	0.0338
UCHL1	Heart diseases	-0.0421	-0.1366	0.0524	0.3790	0.8469		0.1648	0.0066

TNF	Heart diseases	-0.0274	-0.2107	0.1559	0.7675	0.8469		-0.2392	0.0502
TIMP3	Heart diseases	0.5872	-0.1031	1.2774	0.0947	0.8469		-0.8934	0.0518
TAFA5	Heart diseases	0.1571	-0.3008	0.6150	0.4979	0.8469		0.6436	0.0364
p-tau217	Heart diseases	-0.0618	-0.2722	0.1486	0.5617	0.8469		0.4046	0.0028
NPTX1	Heart diseases	-0.0797	-0.3204	0.1610	0.5131	0.8469		0.3683	0.0183
MME	Heart diseases	0.0921	-0.7585	0.9427	0.8304	0.8469		-1.2234	0.0319
KLK6	Heart diseases	-0.0185	-0.2082	0.1711	0.8469	0.8469		0.2614	0.0370
GDI1	Heart diseases	-0.1553	-0.3840	0.0734	0.1811	0.8469		0.3232	0.0328
FCN2	Heart diseases	0.2453	-1.1738	1.6645	0.7325	0.8469		-2.2423	0.0191
CCL11	Heart diseases	0.0341	-0.2555	0.3238	0.8157	0.8469		0.4018	0.0358
UCHL1	Infectious diseases	-0.0441	-0.1408	0.0526	0.3678	0.6048		0.1567	0.0085
TNF	Infectious diseases	0.0629	-0.1246	0.2504	0.5074	0.6977		-0.2109	0.0753
TIMP3	Infectious diseases	0.3109	-0.3954	1.0171	0.3849	0.6048		-0.8277	0.0688
TAFA5	Infectious diseases	-0.0904	-0.5589	0.3781	0.7029	0.7459		0.5905	0.0504
p-tau217	Infectious diseases	-0.0353	-0.2503	0.1798	0.7459	0.7459		0.3896	0.0041
NPTX1	Infectious diseases	0.0473	-0.1990	0.2936	0.7040	0.7459		0.3514	0.0226
MME	Infectious diseases	0.7873	-0.0830	1.6575	0.0757	0.3516		-1.1273	0.0420
KLK6	Infectious diseases	-0.1524	-0.3464	0.0417	0.1225	0.3516		0.2264	0.0654
GDI1	Infectious diseases	-0.1418	-0.3758	0.0922	0.2324	0.5113		0.2706	0.0735
FCN2	Infectious diseases	1.1239	-0.3280	2.5759	0.1278	0.3516		-1.7699	0.0609
CCL11	Infectious diseases	-0.3386	-0.6349	-0.0422	0.0255	0.2810	*	0.3835	0.0349
UCHL1	Respiratory diseases	-0.0198	-0.2717	0.2322	0.8766	0.9643		0.1561	0.0086
TNF	Respiratory diseases	-0.3331	-0.8217	0.1556	0.1795	0.3949		-0.2187	0.0682
TIMP3	Respiratory diseases	2.1161	0.2755	3.9568	0.0246	0.1270	*	-1.1128	0.0132
TAFA5	Respiratory diseases	-0.3363	-1.5572	0.8847	0.5862	0.7165		0.5788	0.0558
p-tau217	Respiratory diseases	0.0104	-0.5457	0.5665	0.9705	0.9705		0.3748	0.0061
NPTX1	Respiratory diseases	0.2529	-0.3890	0.8949	0.4365	0.6859		0.3195	0.0368
MME	Respiratory diseases	-2.4483	-4.7164	-0.1802	0.0346	0.1270	*	-0.9216	0.0884
KLK6	Respiratory diseases	0.5875	0.0818	1.0933	0.0232	0.1270	*	0.1696	0.1619
GDI1	Respiratory diseases	-0.3601	-0.9699	0.2498	0.2445	0.4482		0.3095	0.0393

FCN2	Respiratory diseases	2.8724	-0.9117	6.6564	0.1353	0.3722		-2.2658	0.0173
CCL11	Respiratory diseases	-0.2631	-1.0354	0.5093	0.5010	0.6889		0.3959	0.0333
UCHL1	Metabolic diseases	-0.0567	-0.2738	0.1604	0.6056	0.7878		0.1723	0.0052
TNF	Metabolic diseases	0.2148	-0.2063	0.6358	0.3142	0.7878		-0.2527	0.0405
TIMP3	Metabolic diseases	0.1306	-1.4554	1.7167	0.8706	0.8706		-1.0629	0.0233
TAFA5	Metabolic diseases	0.3440	-0.7081	1.3960	0.5183	0.7878		0.5979	0.0462
p-tau217	Metabolic diseases	0.1429	-0.3359	0.6217	0.5554	0.7878		0.3768	0.0078
NPTX1	Metabolic diseases	-0.2742	-0.8273	0.2790	0.3281	0.7878		0.3539	0.0253
MME	Metabolic diseases	-0.9221	-2.8764	1.0323	0.3517	0.7878		-1.0362	0.0674
KLK6	Metabolic diseases	-0.1954	-0.6312	0.2404	0.3760	0.7878		0.2577	0.0412
GDI1	Metabolic diseases	0.1176	-0.4079	0.6431	0.6582	0.7878		0.3185	0.0355
FCN2	Metabolic diseases	-0.5995	-3.8601	2.6611	0.7162	0.7878		-2.4008	0.0135
CCL11	Metabolic diseases	0.4767	-0.1888	1.1422	0.1585	0.7878		0.3816	0.0463

Supplementary Table 13. Comparison of plasma protein expression between CU and CI (in amyloid-positive participants) after adjusting for other pathologies (first 50 results by Adj. *P* value). The table displays the linear regression results testing the association between cognitive status and each plasma protein, after adjusting for other pathologies. For each protein, the standardized beta values indicate the regression coefficient associated with cognitive impairment, adjusted for age, sex, average protein level and other pathologies. Proteins were classified as FDR-significant, nominally significant (P value) or not significant based on Benjamini–Hochberg-adjusted P values (Adj. P value) and nominal P values (P value). P values were derived from two-sided tests of the regression coefficient.

Adj. P value = Benjamini–Hochberg FDR-adjusted P value; B = regression coefficient (β estimate); Std. error = standard error of the regression coefficient; t = t statistic; logFC = log fold change; log10P = −log10(P value).

**Protein**	**B**	**Std.error**	**t**	**P value**	**logFC**	**log10P**	**Adj. P value**	**Significance**
p-tau217	0.388	0.140	2.763	0.007	0.388	2.170	0.416	nominal
UCHL1	0.181	0.064	2.853	0.005	0.181	2.285	0.416	nominal
FCN2	-2.179	0.955	-2.281	0.025	-2.179	1.610	0.497	nominal
GDI1	0.342	0.154	2.224	0.028	0.342	1.549	0.497	nominal

IL2	0.461	0.206	2.236	0.027	0.461	1.561	0.497	nominal
NPTX1	0.401	0.162	2.476	0.015	0.401	1.828	0.497	nominal
TIMP3	-1.097	0.465	-2.362	0.020	-1.097	1.700	0.497	nominal
TAFA5	0.652	0.308	2.115	0.037	0.652	1.434	0.565	nominal
CCL11	0.383	0.195	1.962	0.052	0.383	1.281	0.585	none
GDNF	1.055	0.523	2.016	0.046	1.055	1.334	0.585	nominal
SOD1	0.473	0.241	1.967	0.052	0.473	1.286	0.585	none
p-tau231	0.446	0.236	1.893	0.061	0.446	1.214	0.626	none
CCL26	-0.438	0.251	-1.749	0.083	-0.438	1.080	0.639	none
KLK6	0.225	0.128	1.759	0.081	0.225	1.089	0.639	none
SFTPD	0.354	0.198	1.791	0.076	0.354	1.119	0.639	none
SNAP25	0.100	0.056	1.787	0.077	0.100	1.115	0.639	none
ANXA5	-0.947	0.633	-1.495	0.138	-0.947	0.860	0.681	none
APOE	-0.234	0.153	-1.534	0.128	-0.234	0.893	0.681	none
AB42	-0.355	0.248	-1.432	0.155	-0.355	0.810	0.681	none
BDNF	-0.564	0.394	-1.432	0.155	-0.564	0.810	0.681	none
FLT1	-0.348	0.222	-1.566	0.120	-0.348	0.919	0.681	none
GFAP	0.390	0.267	1.461	0.147	0.390	0.833	0.681	none
IGFBP7	0.127	0.082	1.548	0.125	0.127	0.905	0.681	none
MME	-0.880	0.572	-1.537	0.127	-0.880	0.896	0.681	none
p-tau181	0.338	0.222	1.524	0.131	0.338	0.884	0.681	none
RUVBL2	0.249	0.172	1.446	0.151	0.249	0.821	0.681	none
SAA1	-0.846	0.574	-1.474	0.143	-0.846	0.844	0.681	none
TNF	-0.193	0.123	-1.563	0.121	-0.193	0.918	0.681	none
CCL22	0.201	0.148	1.358	0.177	0.201	0.751	0.716	none
MAPT	0.302	0.224	1.348	0.180	0.302	0.744	0.716	none
SQSTM1	0.235	0.173	1.357	0.178	0.235	0.750	0.716	none
IL33	0.244	0.187	1.305	0.195	0.244	0.710	0.749	none
IL18	0.199	0.160	1.246	0.215	0.199	0.667	0.786	none
MDH1	0.253	0.207	1.224	0.224	0.253	0.651	0.786	none

TARDBP	-0.276	0.225	-1.225	0.223	-0.276	0.651	0.786	none
CALB2	0.222	0.204	1.087	0.279	0.222	0.554	0.787	none
CCL2	0.250	0.222	1.127	0.262	0.250	0.581	0.787	none
CRP	-0.496	0.438	-1.132	0.260	-0.496	0.585	0.787	none
FOLR1	0.232	0.204	1.138	0.258	0.232	0.589	0.787	none
IGF1R	0.216	0.191	1.129	0.261	0.216	0.583	0.787	none
IL12p70	0.235	0.219	1.077	0.284	0.235	0.547	0.787	none
IL16	-0.154	0.139	-1.108	0.270	-0.154	0.568	0.787	none
KDR	-0.213	0.195	-1.096	0.276	-0.213	0.560	0.787	none
NPTX2	0.138	0.129	1.068	0.288	0.138	0.541	0.787	none
POSTN	0.300	0.278	1.078	0.283	0.300	0.548	0.787	none
IL6R	-0.452	0.436	-1.037	0.302	-0.452	0.520	0.807	none
CHIT1	0.657	0.660	0.996	0.321	0.657	0.493	0.809	none
CST3	0.057	0.057	0.995	0.322	0.057	0.492	0.809	none
OligoSNCA	0.449	0.445	1.009	0.315	0.449	0.502	0.809	none
pSNCA129	0.231	0.238	0.973	0.333	0.231	0.478	0.818	none

Supplementary Table 14. Comparison of plasma protein expression between CU and CI in amyloid-negative participants (first 50 results by Adj. P value). The table displays the linear regression results testing the association between cognitive status (in amyloid-negative participants) and each plasma protein. For each protein, the standardized beta values indicate the regression coefficient associated with cognitive impairment, adjusted for age, sex and average protein level. Proteins were classified as FDR-significant, nominally significant (P value) or not significant based on Benjamini–Hochberg-adjusted P values (Adj. P value) and nominal P values (P value). P values were derived from two-sided tests of the regression coefficient.

Adj. P value = Benjamini–Hochberg FDR-adjusted P value; B = regression coefficient (β estimate); Std. error = standard error of the regression coefficient; t = t statistic; logFC = log fold change; log10P = −log10(P value).

**Protein**	**B**	**Std.error**	**t**	**P value**	**logFC**	**log10P**	**Adj. P value**	**Significance**
GFAP	0.3786	0.1051	3.6030	0.0003	0.3786	3.4757	0.0352	FDR
NEFL	0.4320	0.1249	3.4584	0.0006	0.4320	3.2420	0.0352	FDR

IGFBP7	0.1169	0.0375	3.1152	0.0019	0.1169	2.7201	0.0625	nominal
NPTX2	0.1796	0.0580	3.0957	0.0020	0.1796	2.6918	0.0625	nominal
CSF2	-0.2862	0.0969	-2.9538	0.0032	-0.2862	2.4904	0.0765	nominal
SNAP25	0.0637	0.0219	2.9088	0.0037	0.0637	2.4283	0.0765	nominal
NGF	-0.2047	0.0753	-2.7182	0.0067	-0.2047	2.1733	0.0828	nominal
SLIT2	0.1201	0.0439	2.7323	0.0064	0.1201	2.1917	0.0828	nominal
SNCB	0.1548	0.0569	2.7216	0.0066	0.1548	2.1778	0.0828	nominal
TREM1	0.2558	0.0941	2.7170	0.0067	0.2558	2.1718	0.0828	nominal
CALB2	0.2364	0.0906	2.6090	0.0093	0.2364	2.0337	0.1035	nominal
CCL26	-0.3293	0.1313	-2.5074	0.0124	-0.3293	1.9078	0.1170	nominal
S100B	-0.2859	0.1136	-2.5152	0.0121	-0.2859	1.9174	0.1170	nominal
GDI1	0.1396	0.0574	2.4335	0.0152	0.1396	1.8189	0.1264	nominal
UCHL1	-0.1275	0.0525	-2.4278	0.0154	-0.1275	1.8121	0.1264	nominal
IFNG	-0.4054	0.1717	-2.3613	0.0185	-0.4054	1.7339	0.1412	nominal
SFTPD	0.1987	0.0849	2.3404	0.0195	0.1987	1.7097	0.1412	nominal
MSLN	0.2041	0.0905	2.2544	0.0244	0.2041	1.6118	0.1671	nominal
VEGFD	0.2394	0.1076	2.2261	0.0263	0.2394	1.5802	0.1702	nominal
APOE	-0.1480	0.0684	-2.1636	0.0308	-0.1480	1.5115	0.1808	nominal
VCAM1	-0.1532	0.0708	-2.1627	0.0309	-0.1532	1.5105	0.1808	nominal
HTT	-0.2203	0.1046	-2.1053	0.0356	-0.2203	1.4487	0.1989	nominal
BASP1	-0.5397	0.2707	-1.9940	0.0465	-0.5397	1.3326	0.2345	nominal
CHIT1	0.5741	0.2849	2.0149	0.0443	0.5741	1.3540	0.2345	nominal
PDGFRB	0.1966	0.0991	1.9836	0.0477	0.1966	1.3219	0.2345	nominal
PSEN1	-0.1344	0.0705	-1.9071	0.0569	-0.1344	1.2451	0.2497	none
p-tau181	-0.1068	0.0564	-1.8919	0.0589	-0.1068	1.2301	0.2497	none
p-tau217	0.0997	0.0526	1.8961	0.0583	0.0997	1.2342	0.2497	none
SAA1	-0.4888	0.2522	-1.9384	0.0529	-0.4888	1.2763	0.2497	none
NPTX1	0.1404	0.0772	1.8173	0.0696	0.1404	1.1577	0.2852	none
VGF	-0.4020	0.2367	-1.6981	0.0899	-0.4020	1.0463	0.3498	none
YWHAZ	-0.2192	0.1295	-1.6922	0.0910	-0.2192	1.0409	0.3498	none

AB40	0.2081	0.1270	1.6391	0.1016	0.2081	0.9931	0.3787	none
CCL3	0.3332	0.2118	1.5736	0.1160	0.3332	0.9356	0.4196	none
AB38	0.2166	0.1398	1.5496	0.1216	0.2166	0.9150	0.4266	none
PARK7	0.2348	0.1528	1.5364	0.1249	0.2348	0.9036	0.4266	none
FGF2	-0.1963	0.1303	-1.5060	0.1325	-0.1963	0.8778	0.4288	none
PGF	-0.0782	0.0515	-1.5174	0.1296	-0.0782	0.8875	0.4288	none
IGF1R	0.1010	0.0686	1.4725	0.1413	0.1010	0.8499	0.4345	none
TREM2	0.1159	0.0785	1.4775	0.1399	0.1159	0.8541	0.4345	none
AGRN	0.0599	0.0418	1.4351	0.1516	0.0599	0.8192	0.4549	none
NPTXR	0.0982	0.0693	1.4167	0.1570	0.0982	0.8042	0.4597	none
MME	-0.3442	0.2479	-1.3884	0.1654	-0.3442	0.7815	0.4731	none
CX3CL1	0.0656	0.0521	1.2597	0.2082	0.0656	0.6816	0.5819	none
POSTN	0.1064	0.0857	1.2417	0.2147	0.1064	0.6681	0.5869	none
MDH1	-0.1019	0.0836	-1.2193	0.2231	-0.1019	0.6515	0.5965	none
BACE1	-0.0773	0.0682	-1.1338	0.2572	-0.0773	0.5897	0.5967	none
CNTN2	0.2454	0.2186	1.1226	0.2619	0.2454	0.5818	0.5967	none
IL10	-0.1859	0.1612	-1.1538	0.2489	-0.1859	0.6039	0.5967	none
IL13	0.1787	0.1585	1.1275	0.2599	0.1787	0.5852	0.5967	none

Supplementary Table 15. Results from Gene Ontology for protein differentially expressed in CU and CI in amyloid-negative groups. For each term, the table lists: the group, the p-value corrected for multiple testing (Padj, FDR-corrected), the GO identifier (Term name); the specific genes from the query set that overlap with the GO term (Intersection), and the general Signature for each pathway.

CU= cognitively unimpaired; CI= cognitively impaired; Aβ-= amyloid-negative; Aβ+=amyloid-positive.

Group	Padj	term_id	Term name	Intersection	Signature
CU Aβ-	5E-06	GO:0005102	signaling receptor binding	IFNG, CCL26, CSF2, S100B, NGF, VCAM1, APOE, UCHL1	signaling receptor binding

CU Aβ-	1E-04	GO:0048018	receptor ligand activity	IFNG, CCL26, CSF2, NGF, APOE	signaling receptor binding
CU Aβ-	1E-04	GO:0030546	signaling receptor activator activity	IFNG, CCL26, CSF2, NGF, APOE	signaling receptor binding
CU Aβ-	2E-04	GO:0030545	signaling receptor regulator activity	IFNG, CCL26, CSF2, NGF, APOE	signaling receptor binding
CU Aβ-	2E-04	GO:0005126	cytokine receptor binding	IFNG, CCL26, CSF2, NGF	signaling receptor binding
CU Aβ-	4E-04	GO:0008283	cell population proliferation	IFNG, CCL26, CSF2, S100B, NGF, VCAM1, APOE, UCHL1	cell population proliferation
CU Aβ-	1E-03	GO:0032747	positive regulation of interleukin-23 production	IFNG, CSF2	cell population proliferation
CU Aβ-	1E-03	GO:0097191	extrinsic apoptotic signaling pathway	IFNG, CSF2, HTT, NGF	signaling receptor binding
CI Aβ-	1E-03	GO:0098916	anterograde trans-synaptic signaling	SNAP25, SNCB, NPTX2, CALB2, GFAP, NEFL	signaling
CI Aβ-	1E-03	GO:0099536	synaptic signaling	SNAP25, SNCB, NPTX2, CALB2, GFAP, NEFL	signaling
CI Aβ-	1E-03	GO:0099537	trans-synaptic signaling	SNAP25, SNCB, NPTX2, CALB2, GFAP, NEFL	signaling
CI Aβ-	1E-03	GO:0051414	response to cortisol	IGFBP7, SLIT2	response to stimulus
CI Aβ-	1E-03	GO:0060326	cell chemotaxis	SLIT2, PDGFRB, SFTPD, VEGFD, TREM1	chemotaxis
CI Aβ-	1E-03	GO:0006935	chemotaxis	SLIT2, PDGFRB, SFTPD, VEGFD, TREM1	chemotaxis
CI Aβ-	1E-03	GO:0071670	smooth muscle cell chemotaxis	SLIT2, PDGFRB	chemotaxis
CI Aβ-	1E-03	GO:0010975	regulation of neuron projection development	SNAP25, SLIT2, GDI1, GFAP, NEFL	response to stimulus

Supplementary Table 16. Linear regression analysis showing associations between pathologies and protein levels, adjusted for cognitive status, age, sex and average protein level. The table reports regression coefficients (β) with 95% confidence intervals

(CIs), exact P values, and Benjamini–Hochberg FDR-adjusted q-values. Significance is indicated by * (nominal p<0.05) and ** (FDR q<0.05). Exact P values and FDR-adjusted q values are provided.

β = beta estimate; CI = confidence interval; FDR = false discovery rate; Sig. = significance.

**Protein**	**Exposure**	**Effect size (β)**	**CI (lower)**	**CI (higher)**	***P value***	**Q value**	**Sig.**	**Effect size (β) Group**	***P value* Group**
VEGFD	Cholesterol	0.0071	-0.1625	0.1766	0.9348	0.9348		0.0977	0.4247
VCAM1	Cholesterol	0.0271	-0.0927	0.1469	0.6567	0.7138		-0.1786	0.0352
UCHL1	Cholesterol	-0.1022	-0.1934	-0.0111	0.0280	0.1734	*	-0.1390	0.0313
TREM1	Cholesterol	-0.1299	-0.2838	0.0241	0.0981	0.3065		0.2550	0.0220
SNCB	Cholesterol	-0.1396	-0.2299	-0.0492	0.0025	0.0629	*	0.1360	0.0378
SNAP25	Cholesterol	-0.0474	-0.0809	-0.0140	0.0055	0.0694	*	0.0580	0.0169
SLIT2	Cholesterol	0.0200	-0.0490	0.0890	0.5691	0.6828		0.1414	0.0048
SFTPD	Cholesterol	0.0439	-0.0921	0.1799	0.5261	0.6828		0.1959	0.0460
S100B	Cholesterol	-0.2020	-0.3768	-0.0272	0.0236	0.1734	*	-0.2990	0.0189
PDGFRB	Cholesterol	0.1136	-0.0428	0.2700	0.1543	0.4285		0.2112	0.0614
NPTX2	Cholesterol	-0.0247	-0.1152	0.0657	0.5911	0.6828		0.2044	0.0019
NGF	Cholesterol	-0.1122	-0.2337	0.0093	0.0702	0.2926		-0.2355	0.0071
NEFL	Cholesterol	-0.0930	-0.2939	0.1079	0.3634	0.5942		0.4300	0.0032
MSLN	Cholesterol	-0.0713	-0.2118	0.0692	0.3193	0.5942		0.2166	0.0332
IGFBP7	Cholesterol	0.0163	-0.0447	0.0772	0.6009	0.6828		0.1381	0.0016
IFNG	Cholesterol	0.2299	-0.0350	0.4949	0.0888	0.3065		-0.3979	0.0370
HTT	Cholesterol	-0.1761	-0.3394	-0.0127	0.0347	0.1734	*	-0.2092	0.0774
GFAP	Cholesterol	0.0849	-0.0772	0.2471	0.3041	0.5942		0.4558	0.0001
GDI1	Cholesterol	-0.0408	-0.1322	0.0505	0.3803	0.5942		0.1653	0.0134
CSF2	Cholesterol	0.0702	-0.0732	0.2136	0.3365	0.5942		-0.2471	0.0251
CHIT1	Cholesterol	-0.1025	-0.4861	0.2811	0.5999	0.6828		0.5381	0.0499
CCL26	Cholesterol	0.0636	-0.1394	0.2666	0.5385	0.6828		-0.4104	0.0056
CALB2	Cholesterol	-0.0181	-0.1550	0.1188	0.7953	0.8285		0.2976	0.0029

BASP1	Cholesterol	0.2625	-0.1598	0.6848	0.2227	0.5148		-0.5382	0.0786
APOE	Cholesterol	-0.0649	-0.1702	0.0404	0.2265	0.5148		-0.1354	0.0774
VEGFD	Other conditions	0.0022	-0.2831	0.2874	0.9880	0.9880		0.1013	0.4106
VCAM1	Other conditions	-0.0133	-0.2148	0.1883	0.8973	0.9880		-0.1784	0.0365
UCHL1	Other conditions	-0.0023	-0.1557	0.1510	0.9761	0.9880		-0.1414	0.0295
TREM1	Other conditions	-0.1207	-0.3797	0.1383	0.3604	0.9314		0.2442	0.0291
SNCB	Other conditions	-0.0831	-0.2351	0.0689	0.2834	0.9314		0.1256	0.0560
SNAP25	Other conditions	-0.0298	-0.0861	0.0265	0.2987	0.9314		0.0536	0.0279
SLIT2	Other conditions	-0.0208	-0.1369	0.0953	0.7248	0.9880		0.1392	0.0058
SFTPD	Other conditions	0.0176	-0.2112	0.2463	0.8801	0.9880		0.1984	0.0446
S100B	Other conditions	0.1103	-0.1839	0.4044	0.4618	0.9314		-0.2897	0.0237
PDGFRB	Other conditions	0.0205	-0.2427	0.2836	0.8787	0.9880		0.2156	0.0575
NPTX2	Other conditions	0.0960	-0.0561	0.2481	0.2156	0.9314		0.2107	0.0014
NGF	Other conditions	-0.0609	-0.2653	0.1435	0.5588	0.9314		-0.2401	0.0063
NEFL	Other conditions	-0.0144	-0.3523	0.3236	0.9335	0.9880		0.4260	0.0037
MSLN	Other conditions	-0.0734	-0.3098	0.1631	0.5424	0.9314		0.2145	0.0361
IGFBP7	Other conditions	0.0837	-0.0189	0.1863	0.1097	0.9137		0.1462	0.0009
IFNG	Other conditions	0.1679	-0.2779	0.6136	0.4598	0.9314		-0.3860	0.0443
HTT	Other conditions	-0.0173	-0.2921	0.2575	0.9018	0.9880		-0.2093	0.0793
GFAP	Other conditions	0.1135	-0.1593	0.3863	0.4142	0.9314		0.4630	0.0001
GDI1	Other conditions	-0.0115	-0.1652	0.1422	0.8832	0.9880		0.1630	0.0154
CSF2	Other conditions	0.1804	-0.0609	0.4216	0.1426	0.9137		-0.2290	0.0385
CHIT1	Other conditions	0.3454	-0.2988	0.9895	0.2927	0.9314		0.5665	0.0402
CCL26	Other conditions	0.2530	-0.0885	0.5946	0.1462	0.9137		-0.3893	0.0088
CALB2	Other conditions	0.0237	-0.2067	0.2541	0.8398	0.9880		0.2944	0.0034
BASP1	Other conditions	0.2304	-0.4802	0.9409	0.5245	0.9314		-0.5032	0.1018
APOE	Other conditions	0.1580	-0.0191	0.3352	0.0803	0.9137		-0.1248	0.1051
VEGFD	Heart diseases	-0.0885	-0.2624	0.0854	0.3181	0.5680		0.0879	0.4747
VCAM1	Heart diseases	0.0419	-0.0810	0.1647	0.5036	0.6641		-0.1782	0.0377
UCHL1	Heart diseases	-0.0444	-0.1378	0.0491	0.3518	0.5864		-0.1443	0.0269

TREM1	Heart diseases	-0.0417	-0.1996	0.1162	0.6042	0.6641		0.2561	0.0214
SNCB	Heart diseases	0.0223	-0.0704	0.1149	0.6370	0.6641		0.1413	0.0307
SNAP25	Heart diseases	0.0199	-0.0144	0.0542	0.2553	0.5318		0.0604	0.0131
SLIT2	Heart diseases	-0.0250	-0.0957	0.0458	0.4890	0.6641		0.1346	0.0075
SFTPD	Heart diseases	-0.0425	-0.1820	0.0969	0.5494	0.6641		0.1930	0.0516
S100B	Heart diseases	-0.1361	-0.3155	0.0432	0.1365	0.4265		-0.3119	0.0147
PDGFRB	Heart diseases	-0.0480	-0.2085	0.1124	0.5568	0.6641		0.2071	0.0682
NPTX2	Heart diseases	-0.0223	-0.1150	0.0705	0.6376	0.6641		0.2055	0.0018
NGF	Heart diseases	-0.1439	-0.2685	-0.0193	0.0237	0.1672	*	-0.2555	0.0036
NEFL	Heart diseases	0.0590	-0.1471	0.2650	0.5744	0.6641		0.4393	0.0026
MSLN	Heart diseases	0.1133	-0.0308	0.2575	0.1231	0.4265		0.2315	0.0229
IGFBP7	Heart diseases	0.0136	-0.0489	0.0762	0.6688	0.6688		0.1424	0.0013
IFNG	Heart diseases	0.3899	0.1182	0.6617	0.0050	0.1250	*	-0.3622	0.0578
HTT	Heart diseases	-0.1949	-0.3625	-0.0274	0.0226	0.1672	*	-0.2328	0.0490
GFAP	Heart diseases	-0.1805	-0.3468	-0.0142	0.0334	0.1672	*	0.4419	0.0002
GDI1	Heart diseases	0.0481	-0.0456	0.1418	0.3139	0.5680		0.1719	0.0097
CSF2	Heart diseases	0.0966	-0.0505	0.2437	0.1977	0.5240		-0.2406	0.0297
CHIT1	Heart diseases	0.3429	-0.0482	0.7339	0.0856	0.3567		0.5781	0.0366
CCL26	Heart diseases	-0.0654	-0.2736	0.1428	0.5375	0.6641		-0.4231	0.0047
CALB2	Heart diseases	-0.0858	-0.2263	0.0546	0.2306	0.5240		0.2888	0.0037
BASP1	Heart diseases	0.2668	-0.1663	0.7000	0.2268	0.5240		-0.5337	0.0819
APOE	Heart diseases	-0.1199	-0.2279	-0.0120	0.0295	0.1672	*	-0.1517	0.0493
VEGFD	Infectious diseases	0.1407	-0.0428	0.3242	0.1326	0.3314		0.1002	0.4347
VCAM1	Infectious diseases	-0.0168	-0.1465	0.1128	0.7986	0.8681		-0.1908	0.0331
UCHL1	Infectious diseases	-0.0025	-0.1011	0.0962	0.9608	0.9608		-0.1537	0.0244
TREM1	Infectious diseases	-0.1327	-0.2993	0.0339	0.1182	0.3314		0.2768	0.0183
SNCB	Infectious diseases	-0.0517	-0.1495	0.0460	0.2990	0.4671		0.1391	0.0431
SNAP25	Infectious diseases	0.0073	-0.0289	0.0436	0.6904	0.7845		0.0587	0.0213
SLIT2	Infectious diseases	-0.0686	-0.1432	0.0061	0.0719	0.2759		0.1551	0.0029
SFTPD	Infectious diseases	-0.1326	-0.2797	0.0145	0.0773	0.2759		0.2400	0.0187

S100B	Infectious diseases	-0.2147	-0.4039	-0.0255	0.0262	0.2759	*	-0.2344	0.0771
PDGFRB	Infectious diseases	0.0657	-0.1036	0.2349	0.4463	0.5883		0.2210	0.0612
NPTX2	Infectious diseases	-0.1124	-0.2102	-0.0145	0.0244	0.2759	*	0.2275	0.0009
NGF	Infectious diseases	0.0385	-0.0930	0.1701	0.5654	0.6731		-0.2692	0.0035
NEFL	Infectious diseases	-0.1536	-0.3710	0.0638	0.1658	0.3767		0.4808	0.0018
MSLN	Infectious diseases	0.0617	-0.0904	0.2138	0.4260	0.5883		0.2297	0.0305
IGFBP7	Infectious diseases	-0.0256	-0.0915	0.0404	0.4471	0.5883		0.1612	0.0005
IFNG	Infectious diseases	-0.0143	-0.3011	0.2724	0.9218	0.9602		-0.4684	0.0195
HTT	Infectious diseases	0.0956	-0.0812	0.2723	0.2888	0.4671		-0.2456	0.0491
GFAP	Infectious diseases	0.0634	-0.1121	0.2389	0.4781	0.5976		0.4447	0.0003
GDI1	Infectious diseases	-0.0897	-0.1885	0.0092	0.0753	0.2759		0.1867	0.0077
CSF2	Infectious diseases	0.1517	-0.0035	0.3069	0.0554	0.2759		-0.1680	0.1222
CHIT1	Infectious diseases	-0.2304	-0.6459	0.1851	0.2765	0.4671		0.5892	0.0415
CCL26	Infectious diseases	-0.1491	-0.3688	0.0706	0.1832	0.3816		-0.4121	0.0071
CALB2	Infectious diseases	-0.1139	-0.2621	0.0343	0.1316	0.3314		0.3472	0.0009
BASP1	Infectious diseases	0.2945	-0.1625	0.7515	0.2061	0.3964		-0.5362	0.0961
APOE	Infectious diseases	-0.1037	-0.2176	0.0102	0.0744	0.2759		-0.1329	0.0955
VEGFD	Respiratory diseases	-0.2154	-0.5476	0.1168	0.2033	0.7508		0.0951	0.4389
VCAM1	Respiratory diseases	-0.0044	-0.2391	0.2303	0.9705	0.9961		-0.1814	0.0337
UCHL1	Respiratory diseases	-0.0213	-0.1999	0.1573	0.8148	0.9795		-0.1393	0.0322
TREM1	Respiratory diseases	-0.0330	-0.3346	0.2685	0.8297	0.9795		0.2511	0.0249
SNCB	Respiratory diseases	-0.0425	-0.2195	0.1345	0.6374	0.9795		0.1362	0.0382
SNAP25	Respiratory diseases	-0.0133	-0.0788	0.0523	0.6911	0.9795		0.0588	0.0161
SLIT2	Respiratory diseases	0.1343	-0.0009	0.2695	0.0515	0.6041		0.1390	0.0056
SFTPD	Respiratory diseases	0.1345	-0.1319	0.4009	0.3218	0.9795		0.2013	0.0415
S100B	Respiratory diseases	-0.0531	-0.3957	0.2894	0.7608	0.9795		-0.3108	0.0152
PDGFRB	Respiratory diseases	0.0678	-0.2387	0.3742	0.6642	0.9795		0.2074	0.0672
NPTX2	Respiratory diseases	-0.0112	-0.1883	0.1659	0.9011	0.9795		0.2047	0.0019
NGF	Respiratory diseases	-0.0198	-0.2578	0.2182	0.8701	0.9795		-0.2452	0.0052
NEFL	Respiratory diseases	-0.0671	-0.4607	0.3265	0.7379	0.9795		0.4175	0.0045

MSLN	Respiratory diseases	-0.2333	-0.5086	0.0421	0.0967	0.6041		0.2156	0.0345
IGFBP7	Respiratory diseases	0.1284	0.0089	0.2479	0.0352	0.6041	*	0.1430	0.0011
IFNG	Respiratory diseases	0.1484	-0.3708	0.6675	0.5748	0.9795		-0.3907	0.0421
HTT	Respiratory diseases	-0.2456	-0.5656	0.0744	0.1323	0.6614		-0.2071	0.0809
GFAP	Respiratory diseases	0.2739	-0.0437	0.5916	0.0909	0.6041		0.4570	0.0001
GDI1	Respiratory diseases	-0.0795	-0.2585	0.0995	0.3836	0.9795		0.1640	0.0145
CSF2	Respiratory diseases	-0.0647	-0.3457	0.2163	0.6512	0.9795		-0.2445	0.0277
CHIT1	Respiratory diseases	0.1470	-0.5961	0.8902	0.6977	0.9795		0.5475	0.0474
CCL26	Respiratory diseases	0.0469	-0.3508	0.4447	0.8168	0.9795		-0.4199	0.0048
CALB2	Respiratory diseases	0.0653	-0.2030	0.3336	0.6329	0.9795		0.2965	0.0031
BASP1	Respiratory diseases	0.0021	-0.8254	0.8295	0.9961	0.9961		-0.5284	0.0865
APOE	Respiratory diseases	-0.1317	-0.3380	0.0745	0.2102	0.7508		-0.1406	0.0686
VEGFD	Metabolic diseases	0.1632	-0.2341	0.5604	0.4201	0.9169		0.1347	0.2822
VCAM1	Metabolic diseases	0.0219	-0.2587	0.3025	0.8781	0.9227		-0.1780	0.0411
UCHL1	Metabolic diseases	-0.0554	-0.2690	0.1581	0.6104	0.9169		-0.1500	0.0238
TREM1	Metabolic diseases	0.2010	-0.1597	0.5616	0.2741	0.9169		0.2569	0.0235
SNCB	Metabolic diseases	0.0155	-0.1961	0.2271	0.8858	0.9227		0.1469	0.0277
SNAP25	Metabolic diseases	-0.0246	-0.1030	0.0538	0.5377	0.9169		0.0590	0.0166
SLIT2	Metabolic diseases	0.0334	-0.1282	0.1951	0.6848	0.9169		0.1549	0.0026
SFTPD	Metabolic diseases	0.1766	-0.1419	0.4952	0.2766	0.9169		0.2075	0.0384
S100B	Metabolic diseases	-0.0503	-0.4599	0.3593	0.8095	0.9227		-0.3277	0.0114
PDGFRB	Metabolic diseases	0.1486	-0.2179	0.5150	0.4262	0.9169		0.2285	0.0471
NPTX2	Metabolic diseases	0.0620	-0.1498	0.2738	0.5654	0.9169		0.2131	0.0015
NGF	Metabolic diseases	-0.0929	-0.3775	0.1917	0.5217	0.9169		-0.2601	0.0035
NEFL	Metabolic diseases	0.0054	-0.4652	0.4761	0.9820	0.9820		0.4400	0.0030
MSLN	Metabolic diseases	-0.0571	-0.3863	0.2721	0.7335	0.9169		0.2197	0.0355
IGFBP7	Metabolic diseases	0.0554	-0.0875	0.1983	0.4466	0.9169		0.1508	0.0008
IFNG	Metabolic diseases	0.1214	-0.4993	0.7422	0.7009	0.9169		-0.3830	0.0506
HTT	Metabolic diseases	-0.2655	-0.6482	0.1172	0.1735	0.9169		-0.2264	0.0607
GFAP	Metabolic diseases	-0.0366	-0.4165	0.3433	0.8500	0.9227		0.4283	0.0004

GDI1	Metabolic diseases	0.0666	-0.1475	0.2806	0.5415	0.9169		0.1790	0.0086
CSF2	Metabolic diseases	0.0673	-0.2687	0.4033	0.6940	0.9169		-0.2296	0.0421
CHIT1	Metabolic diseases	-0.4992	-1.4217	0.4233	0.2883	0.9169		0.5161	0.0670
CCL26	Metabolic diseases	0.1063	-0.3693	0.5819	0.6608	0.9169		-0.4361	0.0040
CALB2	Metabolic diseases	-0.1003	-0.4212	0.2205	0.5392	0.9169		0.3203	0.0016
BASP1	Metabolic diseases	-0.7789	-1.7683	0.2106	0.1226	0.9169		-0.4917	0.1130
APOE	Metabolic diseases	-0.2234	-0.4701	0.0233	0.0758	0.9169		-0.1421	0.0701

Supplementary Table 17. Comparison of plasma protein expression between CU and CI (in amyloid-negative participants) after adjusting for other pathologies (first 50 results by Adj. *P* value). The table displays the linear regression results testing the association between cognitive status and each plasma protein, after adjusting for other pathologies. For each protein, the standardized beta values indicate the regression coefficient associated with cognitive impairment, adjusted for age, sex, average protein level and other pathologies. Proteins were classified as FDR-significant, nominally significant (P value) or not significant based on Benjamini–Hochberg-adjusted P values (Adj. P value) and nominal P values (P value). P values were derived from two-sided tests of the regression coefficient.

Adj. P value = Benjamini–Hochberg FDR-adjusted P value; B = regression coefficient (β estimate); Std. error = standard error of the regression coefficient; t = t statistic; logFC = log fold change; log10P = −log10(P value).

**Protein**	**B**	**Std.error**	**t**	**P value**	**logFC**	**log10P**	**Adj. P value**	**Significance**
IGFBP7	0.1775	0.0471	3.7709	0.0002	0.1775	3.7453	0.0221	FDR
CALB2	0.3511	0.1057	3.3220	0.0010	0.3511	3.0216	0.0290	FDR
GFAP	0.4125	0.1251	3.2966	0.0010	0.4125	2.9830	0.0290	FDR
NGF	-0.3058	0.0938	-3.2603	0.0012	-0.3058	2.9281	0.0290	FDR
NPTX2	0.2432	0.0698	3.4850	0.0005	0.2432	3.2755	0.0290	FDR
NEFL	0.4823	0.1550	3.1108	0.0020	0.4823	2.7077	0.0402	FDR
GDI1	0.2003	0.0705	2.8416	0.0047	0.2003	2.3324	0.0715	nominal
SLIT2	0.1528	0.0533	2.8695	0.0043	0.1528	2.3701	0.0715	nominal
CCL26	-0.4303	0.1567	-2.7464	0.0062	-0.4303	2.2064	0.0824	nominal
YWHAZ	-0.4448	0.1634	-2.7216	0.0067	-0.4448	2.1742	0.0824	nominal

POSTN	0.2678	0.1069	2.5038	0.0126	0.2678	1.9008	0.1405	nominal
SNAP25	0.0624	0.0258	2.4154	0.0160	0.0624	1.7949	0.1621	nominal
UCHL1	-0.1682	0.0703	-2.3910	0.0171	-0.1682	1.7662	0.1621	nominal
FGF2	-0.3511	0.1557	-2.2544	0.0246	-0.3511	1.6099	0.1743	nominal
HTT	-0.2856	0.1261	-2.2652	0.0239	-0.2856	1.6220	0.1743	nominal
MSLN	0.2429	0.1085	2.2397	0.0255	0.2429	1.5935	0.1743	nominal
p-tau217	0.1460	0.0644	2.2674	0.0237	0.1460	1.6245	0.1743	nominal
TREM1	0.2724	0.1188	2.2931	0.0222	0.2724	1.6535	0.1743	nominal
SAA1	-0.6613	0.2988	-2.2128	0.0273	-0.6613	1.5636	0.1759	nominal
TREM2	0.2084	0.0950	2.1945	0.0286	0.2084	1.5435	0.1759	nominal
CHIT1	0.6395	0.2967	2.1553	0.0316	0.6395	1.5007	0.1809	nominal
S100B	-0.2868	0.1349	-2.1258	0.0340	-0.2868	1.4691	0.1809	nominal
SFTPD	0.2231	0.1049	2.1263	0.0339	0.2231	1.4697	0.1809	nominal
SNCB	0.1471	0.0697	2.1101	0.0353	0.1471	1.4523	0.1809	nominal
AGRN	0.1021	0.0516	1.9788	0.0483	0.1021	1.3158	0.2377	nominal
VCAM1	-0.1808	0.0924	-1.9562	0.0509	-0.1808	1.2930	0.2410	none
IFNG	-0.3898	0.2045	-1.9063	0.0571	-0.3898	1.2432	0.2602	none
APOE	-0.1499	0.0813	-1.8445	0.0656	-0.1499	1.1828	0.2794	none
CCL3	0.4785	0.2604	1.8378	0.0666	0.4785	1.1765	0.2794	none
PDGFRB	0.2206	0.1207	1.8275	0.0681	0.2206	1.1665	0.2794	none
p-tau181	-0.1203	0.0685	-1.7555	0.0797	-0.1203	1.0984	0.3163	none
ICAM1	0.0857	0.0495	1.7317	0.0839	0.0857	1.0764	0.3224	none
ANXA5	-0.4368	0.2747	-1.5903	0.1123	-0.4368	0.9495	0.4098	none
AB38	0.2733	0.1723	1.5861	0.1133	0.2733	0.9459	0.4098	none
AB40	0.2368	0.1562	1.5163	0.1300	0.2368	0.8860	0.4237	none
CST3	0.0495	0.0330	1.4993	0.1343	0.0495	0.8718	0.4237	none
IGF1R	0.1319	0.0851	1.5503	0.1216	0.1319	0.9149	0.4237	none
KLK6	0.0927	0.0614	1.5095	0.1317	0.0927	0.8803	0.4237	none
PSEN1	-0.1259	0.0847	-1.4865	0.1377	-0.1259	0.8611	0.4237	none
TARDBP	-0.1579	0.1062	-1.4861	0.1378	-0.1579	0.8607	0.4237	none

NPTXR	0.1249	0.0878	1.4228	0.1553	0.1249	0.8087	0.4549	none
S100A12	-0.2535	0.1779	-1.4248	0.1548	-0.2535	0.8103	0.4549	none
BASP1	-0.4566	0.3259	-1.4008	0.1618	-0.4566	0.7909	0.4629	none
CD63	0.0715	0.0536	1.3333	0.1830	0.0715	0.7376	0.5050	none
FABP3	0.1133	0.0853	1.3279	0.1847	0.1133	0.7334	0.5050	none
IL2	-0.1466	0.1188	-1.2346	0.2175	-0.1466	0.6626	0.5245	none
NPTX1	0.1224	0.0982	1.2465	0.2131	0.1224	0.6715	0.5245	none
PGK1	-0.2587	0.2020	-1.2810	0.2007	-0.2587	0.6974	0.5245	none
PTN	0.2145	0.1701	1.2607	0.2080	0.2145	0.6820	0.5245	none
SOD1	0.1637	0.1291	1.2684	0.2052	0.1637	0.6879	0.5245	none

Supplementary Table 18. **Demographics of co-pathologies by cognitive status.**
Categorical variables were compared using Pearson's chi-squared test and data presented as n (%). Both tests were performed as two-sided omnibus tests. Exact P values are reported where applicable. N(CU)= 811, N(CI)= 138.

CI= cognitively impaired; CU= cognitively unimpaired; N= number.

	CU (N=811)	CI (N=138)	p-value
**Cholesterol**			0.3
High	363 (44.8%)	60 (43.5%)	
**Heart diseases**			0.022
Yes	340 (41.9%)	73 (52.9%)	
**Metabolic diseases**			0.7

	CU<br>(N=811)	CI<br>(N=138)	p-value
Yes	38 (4.7%)	8 (5.8%)	
**Other conditions**			0.003
Yes	66 (8.1%)	23 (16.7%)	
**Respiratory diseases**			0.9
Yes	49 (6.0%)	8 (5.8%)	
**Infectious diseases**			0.4
Yes	542 (66.8%)	83 (60.1%)	

Supplementary Figures

Supplementary Figure 1. Volcano plot displaying the standardized beta values of the protein associations in plasma (x-axis) against statistical significance −log10(P value) (y-axis) between amyloid-positive (N=152) and amyloid-negative (N=797) individuals, after adjusting for other pathologies. Models were adjusted for age, sex and average of the proteins. P values were derived from two-sided tests of the regression coefficient. Multiple comparisons were adjusted using the Benjamini–Hochberg (BH) false discovery rate (FDR) method. Significant proteins are coloured according to direction of association and significance level, as indicated in the key. Source data are provided as a Source Data file.

FDR= false discovery rate

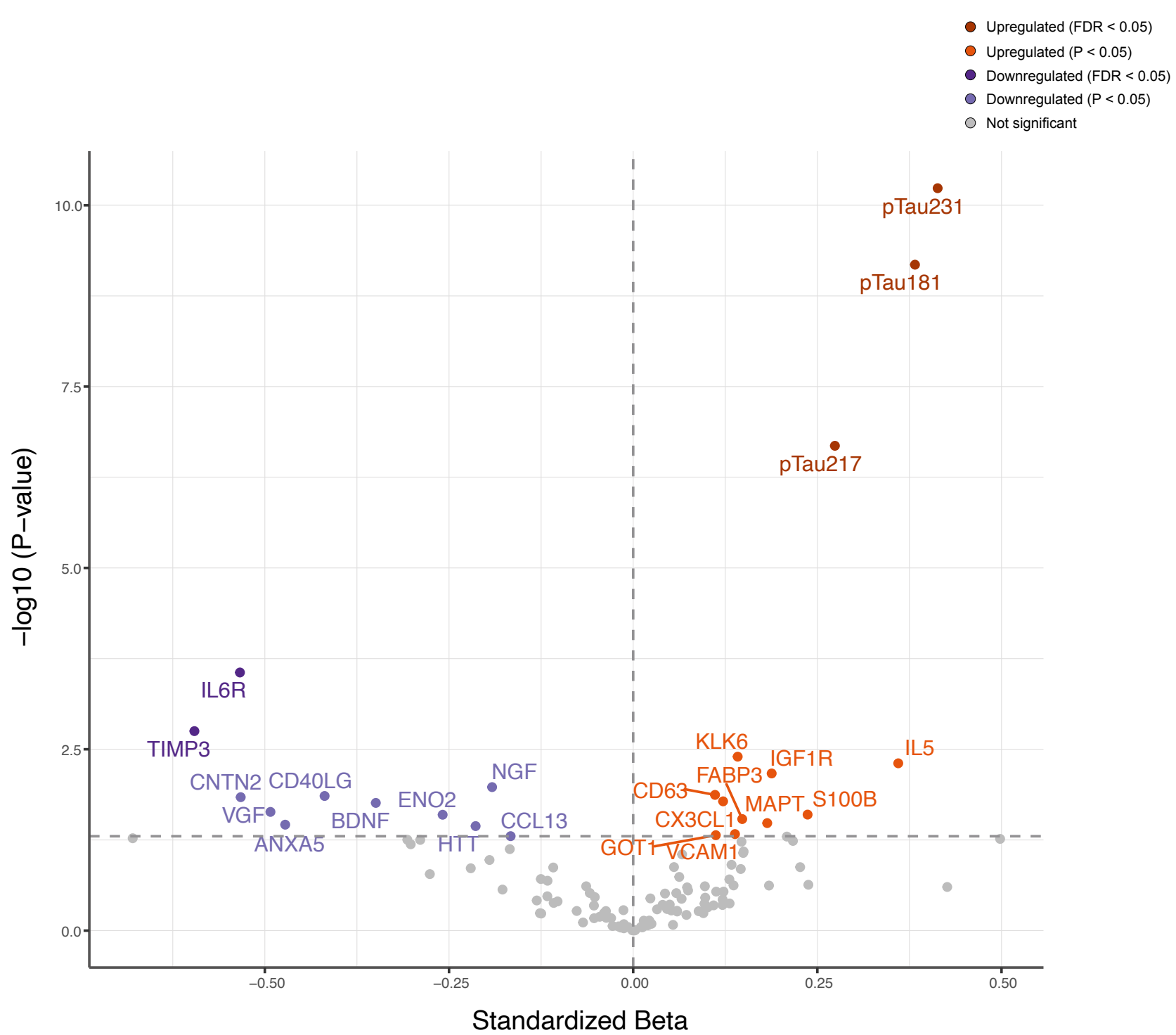

Supplementary Figure 2. Volcano plot displaying the standardized beta values of the protein associations in plasma (x-axis) against statistical significance −log10(P value) (y-axis) between amyloid-positive and amyloid-negative individuals (amyloid positivity defined as values exceeding the 70th percentile of p-tau217 measured with NULISA). Models were adjusted for age, sex and average of the proteins. P values were derived from two-sided tests of the regression coefficient. Multiple comparisons were adjusted using the Benjamini–Hochberg (BH) false discovery rate (FDR) method. Significant proteins are coloured according to direction of association and significance level, as indicated in the key. A) Comparison in VALIANT cohort. B) Comparison in Tanzanian cohort. C) Venn diagram summarizing the comparison of significant proteins derived from the comparison between amyloid-positive and amyloid-negative individuals in VALIANT and Tanzanian cohorts. Intersections indicate proteins shared between cohorts, while non-overlapping regions indicate cohort-specific proteins. Colours correspond to the cohorts as indicated in the figure key. Source data are provided as a Source Data file.

FDR= false discovery rate

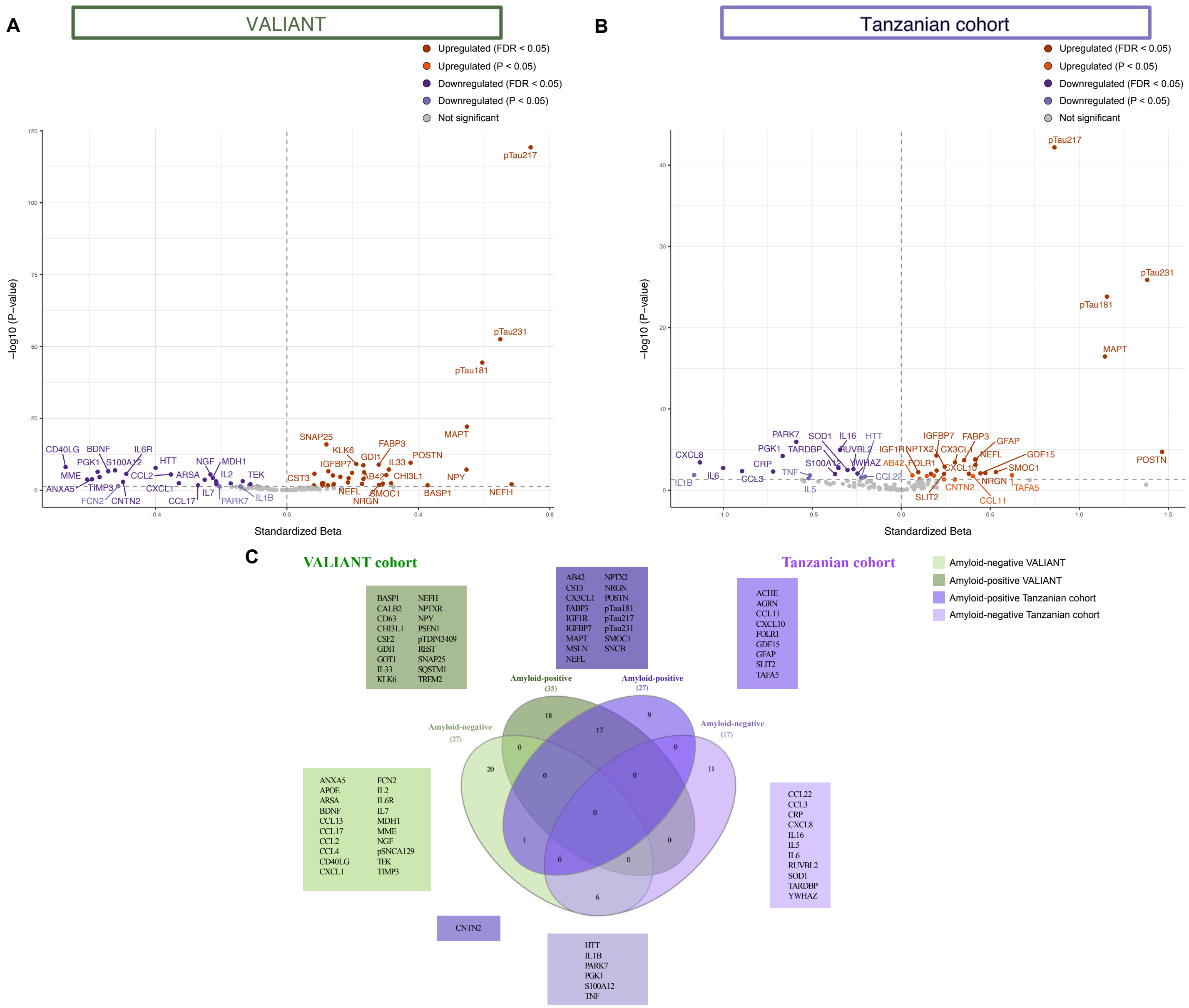
A
VALIANT
B
Tanzanian cohort
Upregulated (FDR < 0.05)
Upregulated (P < 0.05)
Downregulated (FDR < 0.05)
Downregulated (P < 0.05)
Not significant
−log10 (P−value)
Standardized Beta
C
VALIANT cohort
Tanzanian cohort
Amyloid-negative VALIANT
Amyloid-positive VALIANT
Amyloid-positive Tanzanian cohort
Amyloid-negative Tanzanian cohort
BASP1 NEFH CALB2 NPTXR CD63 NPY CHI3L1 PSEN1 CSF2 pTDP43409 GDI1 REST GOT1 SNAP25 IL33 SQSTM1 KLK6 TREM2
AB42 NPTX2 CST3 NRGN CX3CL1 POSTN FABP3 pTau181 IGF1R pTau217 IGFBP7 pTau231 MAPT SMOC1 MSLN SNCB NEFL
ACHE AGRN CCL11 CXCL10 FOLR1 GDF15 GFAP SLIT2 TAFA5
ANXA5 FCN2 APOE IL2 ARSA IL6R BDNF IL7 CCL13 MDH1 CCL17 MME CCL2 NGF CCL4 pSNCA129 CD40LG TEK CXCL1 TIMP3
CCL22 CCL3 CRP CXCL8 IL16 IL5 IL6 RUVBL2 SOD1 TARDBP YWHAZ
CNTN2
HTT IL1B PARK7 PGK1 S100A12 TNF
Amyloid-positive (35)
Amyloid-positive (27)
Amyloid-negative (27)
Amyloid-negative (17)

Supplementary Figure 3. **Volcano plot displaying the standardized beta values of the protein associations in plasma (x-axis) against statistical significance −log10(P value) (y-axis) between CU (N=119) and CI (N=33) in amyloid-positive individuals, after adjusting for other pathologies.** Models were adjusted for age, sex and average of the proteins. P values were derived from two-sided tests of the regression coefficient. Multiple comparisons were adjusted using the Benjamini–Hochberg (BH) false discovery rate (FDR) method. Significant proteins are coloured according to direction of association and significance level, as indicated in the key. Source data are provided as a Source Data file.

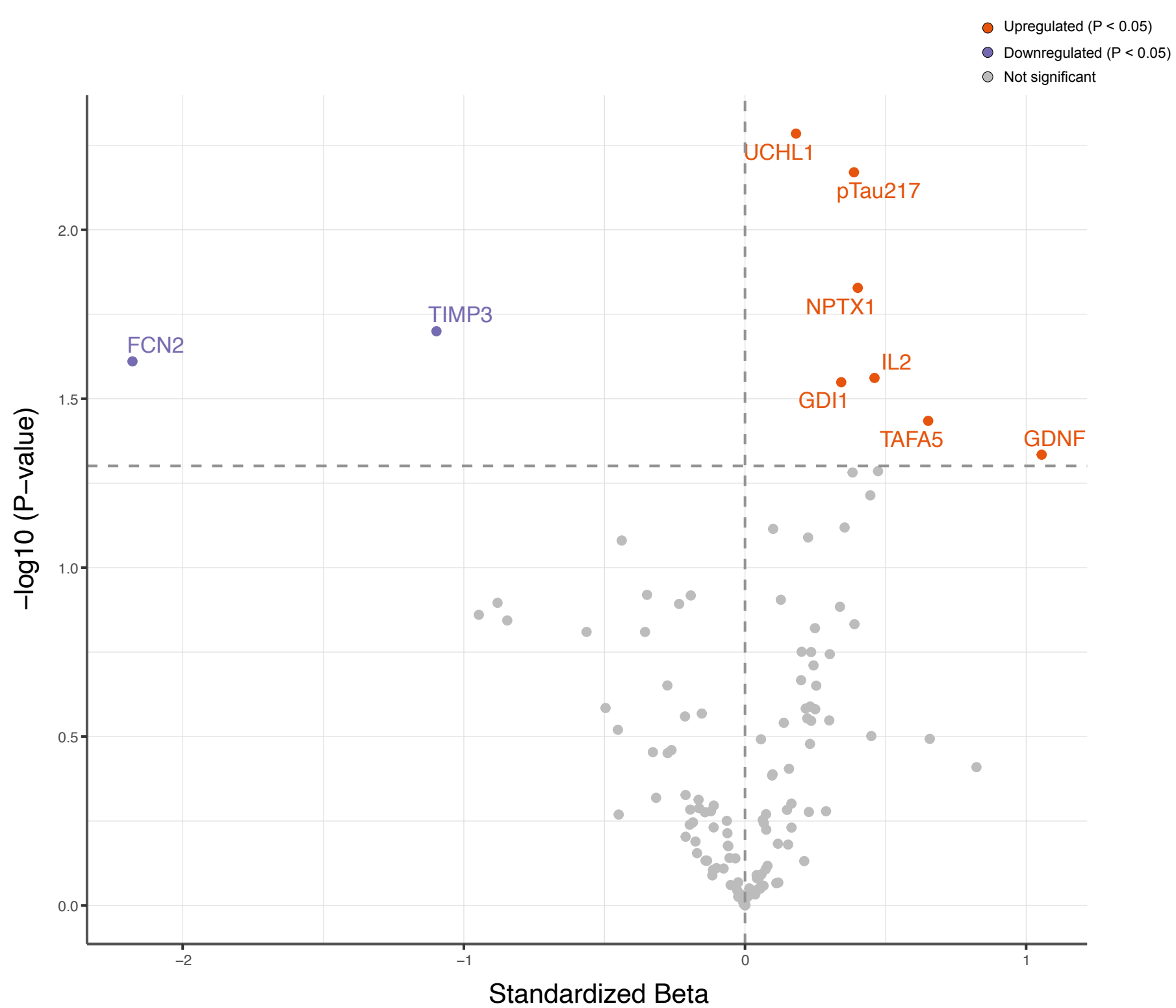

Supplementary Figure 4. Volcano plot displaying the standardized beta values of the protein associations in plasma (x-axis) against statistical significance −log10(P value) (y-axis) between CU (N=692) and CI (N=105) in amyloid-negative individuals, after adjusting for other pathologies. Models were adjusted for age, sex and average of the proteins. P values were derived from two-sided tests of the regression coefficient. Multiple comparisons were adjusted using the Benjamini–Hochberg (BH) false discovery rate (FDR) method. Significant proteins are coloured according to direction of association and significance level, as indicated in the key. Source data are provided as a Source Data file.

FDR= false discovery rate

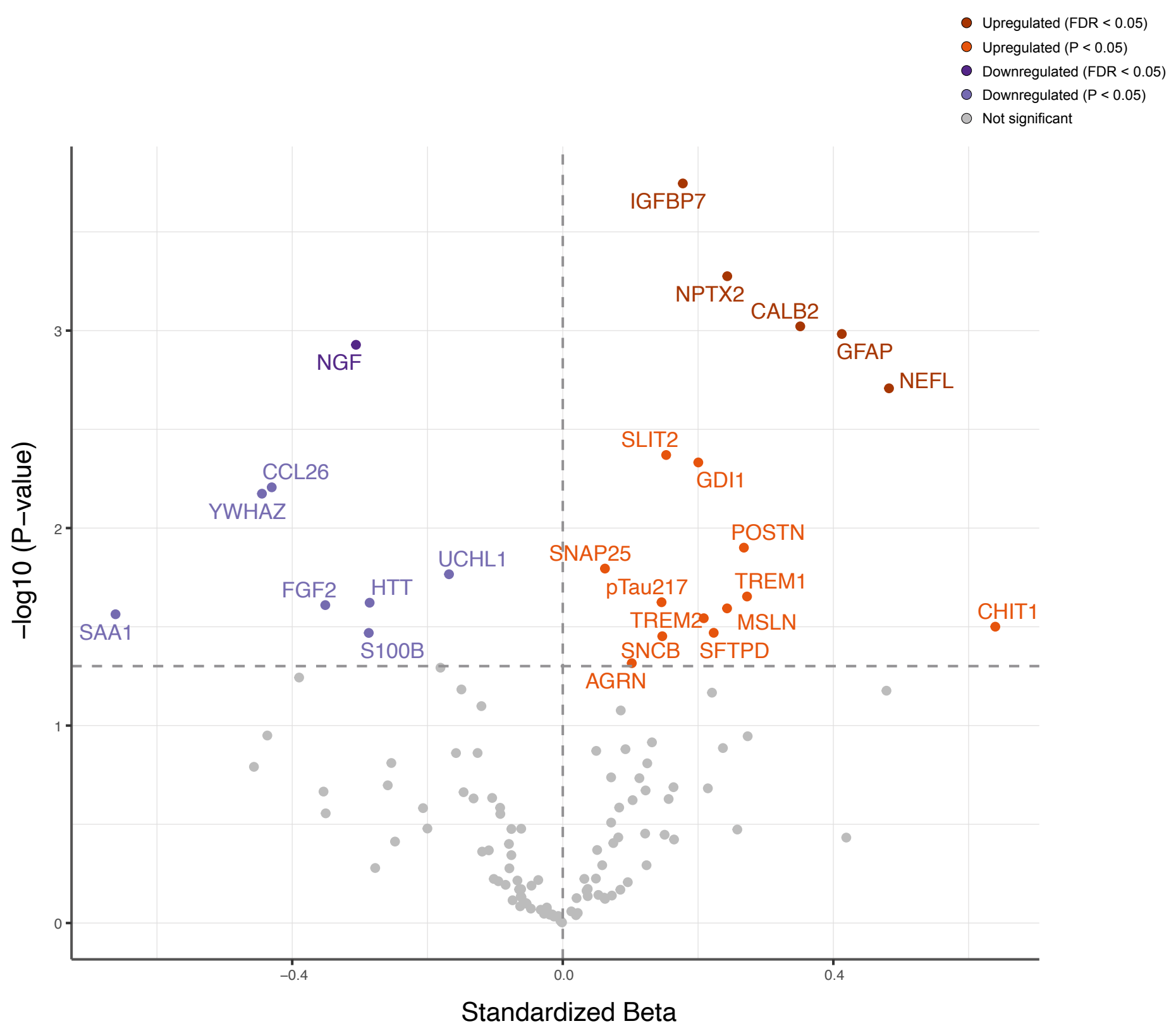

Supplementary Figure 5. Violin plots showing sex-stratified distributions of plasma protein levels by sex. Half violins show the distribution of each protein value for each sex group, with correspondent half boxplots showing the median, interquartile range and whiskers. Sex differences for each protein were assessed using two-sided t-tests. Significance is indicated by * for nominal significance ($P < 0.05$) and ** for FDR-adjusted significance ($q < 0.05$, Benjamini–Hochberg). Source data are provided as a Source Data file.

NPQ = NULISA Protein Quantification.

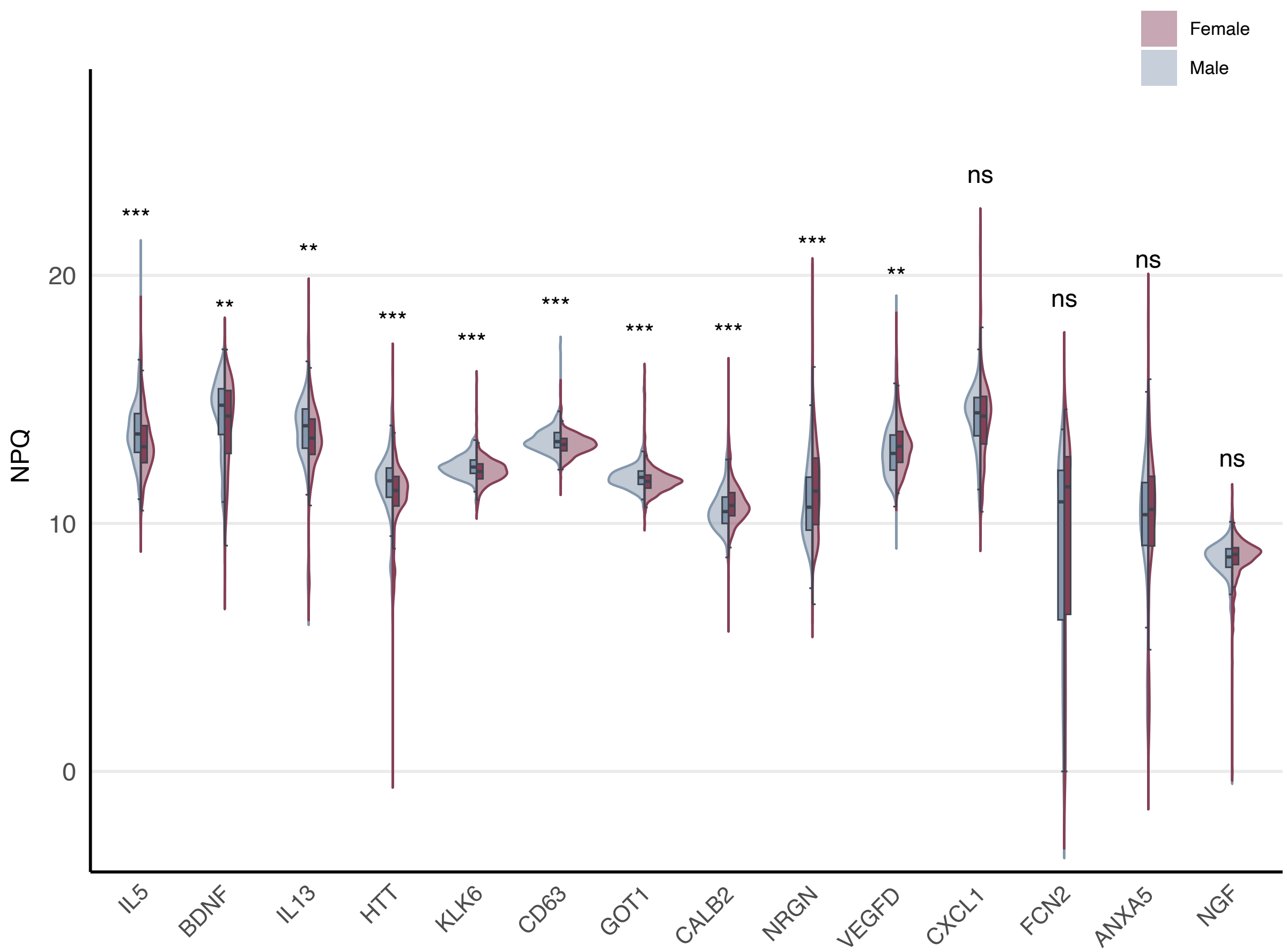

Supplementary Figure 6. Boxplots showing sex-stratified distributions of biomarker levels across amyloid negative (A) and amyloid positive (B) groups. Boxplots showing the median, interquartile range and whiskers. Sex differences for each protein were assessed using two-sided t-tests. Significance is indicated by * for nominal significance ($P < 0.05$) and ** for FDR-adjusted significance ($q < 0.05$, Benjamini–Hochberg). Source data are provided as a Source Data file.

CU-= cognitively unimpaired amyloid-negative; CU+=cognitively unimpaired amyloid-positive; Dem-= dementia amyloid-negative; Dem+= dementia amyloid-positive; MCI-=mild cognitive impairment amyloid-negative; MCI+=mild cognitive impairment amyloid-positive

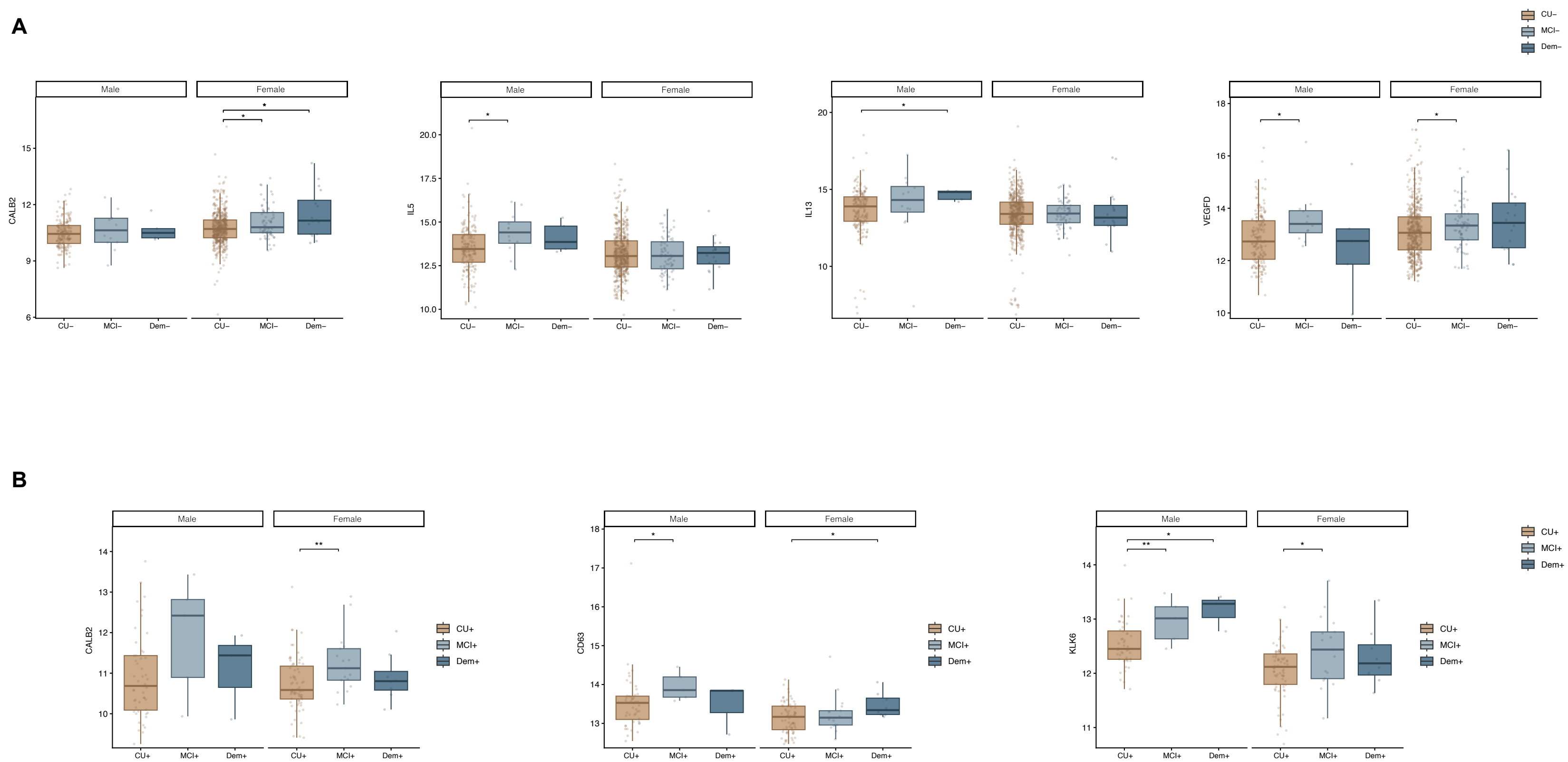

Supplementary Figure 7. Boxplots showing sex-stratified distributions of biomarker z-scored levels across amyloid-positive groups. Data are shown as standardized z-scores (scaled to the cognitively unimpaired Aβ− group) with boxplots showing the median, interquartile range and whiskers. Sex differences for each protein within each group were assessed using two-sided t-tests. Significance is indicated by * for nominal significance ($P < 0.05$) and ** for FDR-adjusted significance ($q < 0.05$, Benjamini–Hochberg). Source data are provided as a Source Data file.

CU+= cognitively unimpaired amyloid-positive; Dem+= dementia amyloid-positive; MCI+=mild cognitive impairment amyloid-positive;

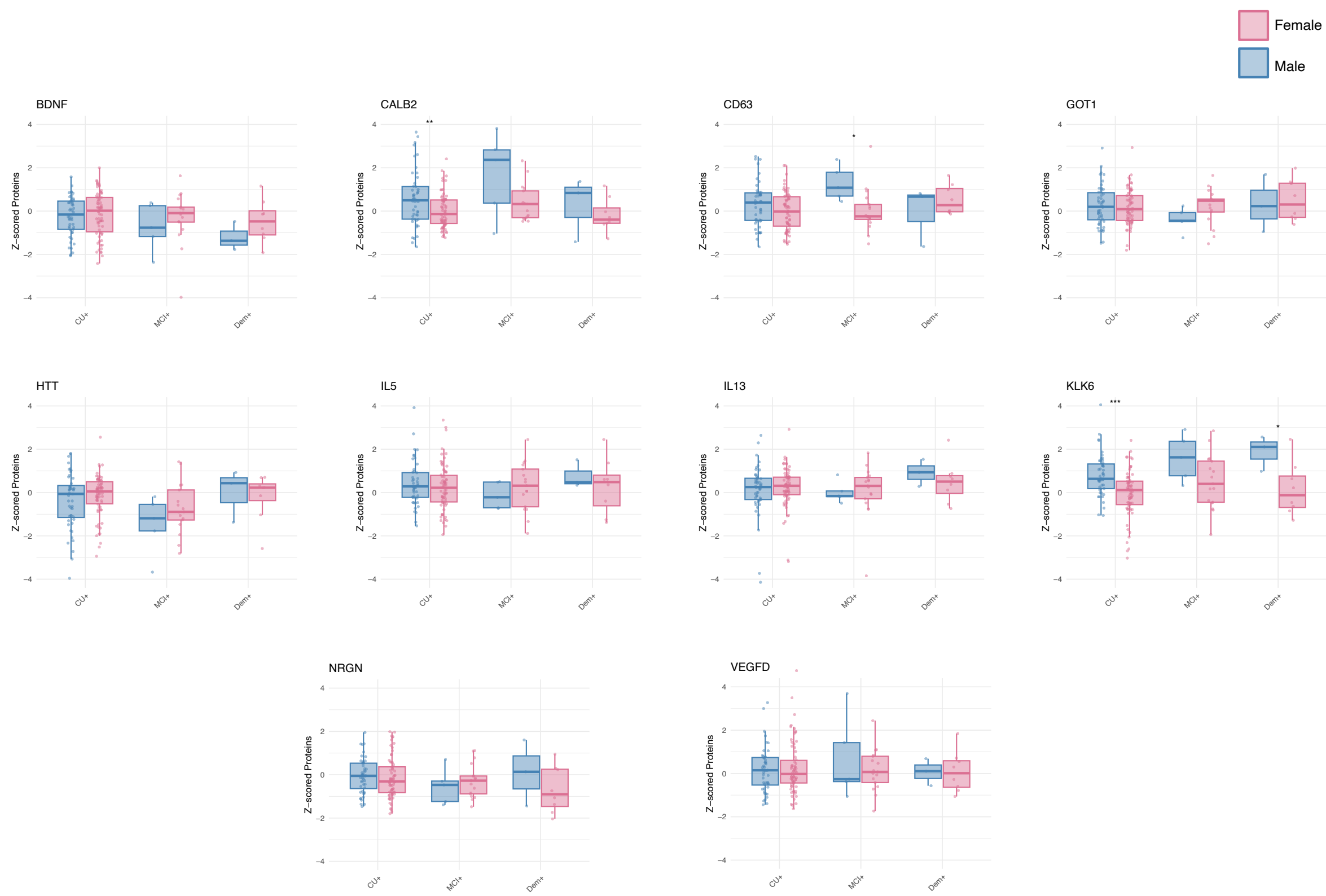

Supplementary Figure 8. Boxplots showing sex-stratified distributions of biomarker z-scored levels across amyloid-negative groups. Data are shown as standardized z-scores (scaled to the cognitively unimpaired Aβ− group) with boxplots showing the median, interquartile range and whiskers. Sex differences for each protein within each group were assessed using two-sided t-tests. Significance is indicated by * for nominal significance ($P < 0.05$) and ** for FDR-adjusted significance ($q < 0.05$, Benjamini–Hochberg). Source data are provided as a Source Data file.

CU-= cognitively unimpaired amyloid-negative; Dem-= dementia amyloid-negative; MCI-=mild cognitive impairment amyloid-negative;

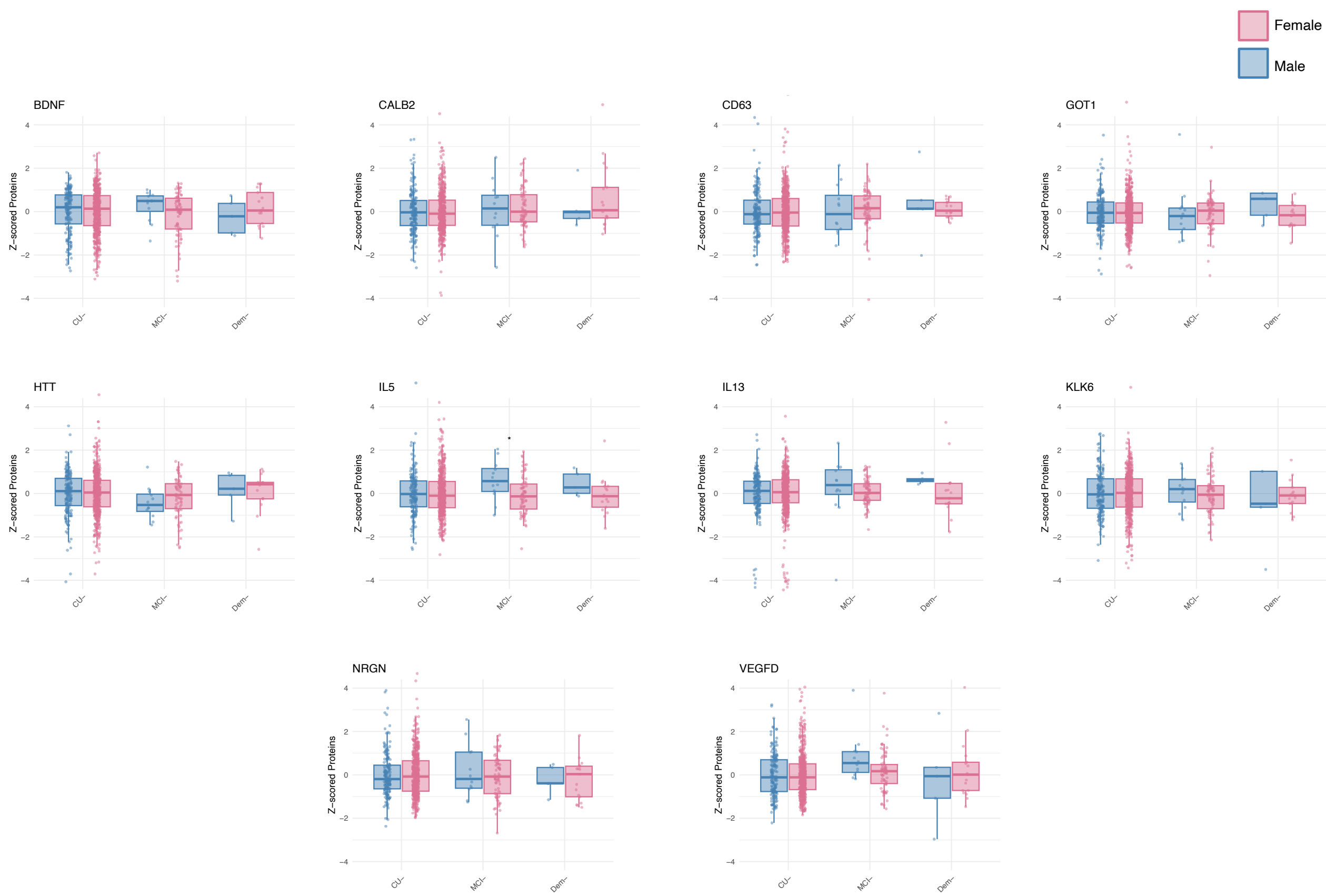

Supplement: Supplementary file 1 [file 41467_2026_74971_MOESM1_ESM.pdf]
