# Supplementary material for: Plasma proteomic profiles of Alzheimer’s disease and neurodegeneration in African cohorts
Source: Nat Commun. 2026 Jul 1;17:8215. doi: 10.1038/s41467-026-74971-4 (PMC13462778; doi:10.1038/s41467-026-74971-4)
Supplement: Supplementary file 2 [file 41467_2026_74971_MOESM2_ESM.pdf]

nature portfolio

Corresponding author(s): Andrea L. Benedet, Rufus O. Akinyemi

Last updated by author(s): 2026/06/09

Reporting Summary

Nature Portfolio wishes to improve the reproducibility of the work that we publish. This form provides structure for consistency and transparency in reporting. For further information on Nature Portfolio policies, see our Editorial Policies and the Editorial Policy Checklist.

Statistics

For all statistical analyses, confirm that the following items are present in the figure legend, table legend, main text, or Methods section.

n/a	Confirmed	
☐	☒	The exact sample size (*n*) for each experimental group/condition, given as a discrete number and unit of measurement
☐	☒	A statement on whether measurements were taken from distinct samples or whether the same sample was measured repeatedly
☐	☒	The statistical test(s) used AND whether they are one- or two-sided<br>*Only common tests should be described solely by name; describe more complex techniques in the Methods section.*
☐	☒	A description of all covariates tested
☐	☒	A description of any assumptions or corrections, such as tests of normality and adjustment for multiple comparisons
☐	☒	A full description of the statistical parameters including central tendency (e.g. means) or other basic estimates (e.g. regression coefficient) AND variation (e.g. standard deviation) or associated estimates of uncertainty (e.g. confidence intervals)
☐	☒	For null hypothesis testing, the test statistic (e.g. *F*, *t*, *r*) with confidence intervals, effect sizes, degrees of freedom and *P* value noted<br>*Give P values as exact values whenever suitable.*
☒	☐	For Bayesian analysis, information on the choice of priors and Markov chain Monte Carlo settings
☒	☐	For hierarchical and complex designs, identification of the appropriate level for tests and full reporting of outcomes
☐	☒	Estimates of effect sizes (e.g. Cohen's *d*, Pearson's *r*), indicating how they were calculated

Our web collection on statistics for biologists contains articles on many of the points above.

Software and code

Policy information about availability of computer code

Data collection	No software was used for data collection.
Data analysis	Data analysis was performed using R (version 4.4.0). The main packages used were "dplyr","tidyr","purrr","broom","ggplot2","ggrepel","table1". Please see: https://github.com/ilariapola/Plasma-proteomic-profiles-of-Alzheimer-s-Disease-and-Neurodegeneration-in-African-Cohorts

For manuscripts utilizing custom algorithms or software that are central to the research but not yet described in published literature, software must be made available to editors and reviewers. We strongly encourage code deposition in a community repository (e.g. GitHub). See the Nature Portfolio guidelines for submitting code & software for further information.

Data

Policy information about availability of data

All manuscripts must include a data availability statement. This statement should provide the following information, where applicable:

- Accession codes, unique identifiers, or web links for publicly available datasets
- A description of any restrictions on data availability
- For clinical datasets or third party data, please ensure that the statement adheres to our policy

Source data are provided with this file. Pseudonymized participant-level data supporting the findings of this study can be shared upon request by contacting the corresponding author, Rufus Akinyemi. Data access will be provided in accordance with applicable ethical approvals, participant consent, and local data protection regulations.

Research involving human participants, their data, or biological material

Policy information about studies with human participants or human data. See also policy information about sex, gender (identity/presentation), and sexual orientation and race, ethnicity and racism.

Reporting on sex and gender	We used the term "sex" throughout the article. Sex was self-reported. Gender information was not collected. Sex was included in first paragraph of the results section where the cohorts are described. Stratified analysis for biological sex are also provided for the VALIANT cohort, where sex differences in protein levels were briefly explored.
Reporting on race, ethnicity, or other socially relevant groupings	This study includes participants from cohorts in Nigeria and Tanzania (Africa), alongside a comparison cohort (TRIAD) from Canada. These groupings were used to capture differences and overlap in proteomic profiles in different geographic, environmental, and socioeconomical contexts between populations from low- and middle-income settings and a high-income setting. Although we have included individuals with European, West African and East African ethnicities in the study, no assumptions or conclusions were atributted to differences based on race or ethnicity, and such variables were not used as proxies for biological traits.
Population characteristics	Overall, 1031 participants were enrolled in the VALIANT study, of which 949 had available blood-based biomarkers and sociodemographic data. The average age of this sample was 65.1 (SD ± 10.4) years, 26.6% were male, the average years of formal education was 5.7 (SD ±4.8) and 14.5% had cognitive impairment, as mild cognitive impairment (MCI) or dementia.<br>To replicate the main analysis, were included 196 participants from the Tanzanian study, of which 196 had available blood-based biomarker data together with age, sex and sociodemographic data; 131 participants had available information on education level (66.5%). The average age of this cohort was 80.3 (SD ± 10.4) years, 34.1% were male, and 61.1% and 40.8% had cognitive impairment as mild cognitive impairment (MCI) or dementia, respectively<br>For a comparative analysis, we utilized matched data from the TRIAD cohort from Canada. “Match 1” included 315 participants, matched by age, sex and clinical status (cognitively unimpaired (CU), MCI, Dementia); “Match 2” included 376 participants matched by age, sex and plasma p-tau217 levels (Simoa); and “Match 3” included 290 participants matched by age, sex and clinicopathological groups, this being a combination of clinical status and amyloid pathology groups–here proxied by Simoa plasma p-tau217 status. For “Match 1” mean age was 70.0 (SD ±8.9) years, 40.6% were male, individuals had on average 15.3 (SD ±3.7) years of education and 39.1% had cognitive impairment. For “Match 2” mean age was 68.9 (SD ±9.1) years, 38.5% were male, individuals had on average 15.1 (SD ±3.7) years of education and 48.9% had cognitive impairment. For “Match 3” mean age was 69.8 (SD ±9.2) years, 43.1% were male, individuals had on average 15.3 (SD ±3.6) years of education and 33.8% had cognitive impairment. Al demographics are included in Table 1 and Supplementary Tables 1,2,3.
Recruitment	Participants from VALIANT cohort were recruited using a multistage sampling method from Yemetu, Ibadan North Local Government Area (LGA), Southwest Nigeria, over 12 months between November 2022 and October 2023. Using the African Rigorous Innovative Stroke Epidemiological Surveillance (ARISES) database as a sampling frame, two wards (Wards 3 and 4) were purposively selected. ARISES is an ongoing observational cohort study in selected wards in Ibadan North and Ibarapa Central LGAs in Oyo State, Nigeria. Participants were included if they were 50 years of age or older,.<br>The Hai dementia cohort from Tanzania consists of community-dwelling adults aged 65 years and over, recruited from six villages within Hai and selected to be representative of upland and lowland areas and different economic status. The study is a community-based door to door dementia prevalence study using the 10/66 protocol.<br>For TRIAD, participants were recruited from the community or outpatients at the McGill University Research Centre for Studies in Aging through advertisements, printed materials, word of mouth, and referrals. Exclusion criteria encompassed inability to speak English or French, inadequate auditory and visual capacities for neuropsychological testing, contraindications for PET or MRI, recent head trauma or major surgery, inadequately treated conditions, current enrollment
Ethics oversight	The VALIANT study was approved by the relevant institutional and national ethics committees in Nigeria (University of Ibadan/University College Hospital (UI/UCH) Health Research Ethics Committee (HREC)). The replication cohorts in Tanzania and the TRIAD cohort were approved by their respective local ethics review boards (National Institute of Medical Research, Dar-es-Salaam, Tanzania; MNI PET working committee and the Douglas Mental Health University Institute Research Ethics Board (MP-18-2017-157)) in accordance with national regulations.

Note that full information on the approval of the study protocol must also be provided in the manuscript.

Field-specific reporting

Please select the one below that is the best fit for your research. If you are not sure, read the appropriate sections before making your selection.

[x] Life sciences [ ] Behavioural & social sciences [ ] Ecological, evolutionary & environmental sciences

For a reference copy of the document with all sections, see nature.com/documents/nr-reporting-summary-flat.pdf

Life sciences study design

All studies must disclose on these points even when the disclosure is negative.

Sample size	No a priori sample size calculation was performed for this study. Sample sizes were determined by the number of participants with available plasma proteomics data, demographic information, and relevant clinical measures in each cohort. We included all eligible participants meeting these criteria, resulting in biomarker data for 949 participants from the VALIANT cohort, 196 participants from the Tanzanian cohort, and 376 participants from the TRIAD cohort. The use of large, well-characterized cohorts and replication of the main findings in an

	independent cohort support the robustness of the analyses.
Data exclusions	No data was excluded from the analysis.<br>All data with available biomarker and sociodemographic data were included.
Replication	To evaluate reproducibility, key findings identified in the discovery cohort were assessed in an independent cohort from Tanzania. Replication analyses were performed independently using the available overlapping biomarkers and the same statistical approach. Consistent findings across the two cohorts support the robustness and generalizability of the results.
Randomization	The data for the present study were obtained from the TRIAD (https://triad.tnl-mcgill.com), VALIANT (https://www.gbhi.org/projects/vascular-health-frailty-and-cognition-ageing-nigerians-study-valiant) and Hai dementia (Tanzania) cohorts, which<br>are observational studies of aging and dementia. No allocation in experimental groups is performed or required for studies such as the one proposed here. Therefore, randomization is not applicable to this study.
Blinding	For all experiments, the investigators were blinded to group allocation during data collection and blinded to any sample information during biomarker analyses

Reporting for specific materials, systems and methods

We require information from authors about some types of materials, experimental systems and methods used in many studies. Here, indicate whether each material, system or method listed is relevant to your study. If you are not sure if a list item applies to your research, read the appropriate section before selecting a response.

Materials & experimental systems

n/a	Involved in the study	
☒	☐	Antibodies
☒	☐	Eukaryotic cell lines
☒	☐	Palaeontology and archaeology
☒	☐	Animals and other organisms
☒	☐	Clinical data
☒	☐	Dual use research of concern
☒	☐	Plants

Methods

n/a	Involved in the study	
☒	☐	ChIP-seq
☒	☐	Flow cytometry
☒	☐	MRI-based neuroimaging

Plants

Seed stocks	*Report on the source of all seed stocks or other plant material used. If applicable, state the seed stock centre and catalogue number. If plant specimens were collected from the field, describe the collection location, date and sampling procedures.*
Novel plant genotypes	*Describe the methods by which all novel plant genotypes were produced. This includes those generated by transgenic approaches, gene editing, chemical/radiation-based mutagenesis and hybridization. For transgenic lines, describe the transformation method, the number of independent lines analyzed and the generation upon which experiments were performed. For gene-edited lines, describe the editor used, the endogenous sequence targeted for editing, the targeting guide RNA sequence (if applicable) and how the editor was applied.*
Authentication	*Describe any authentication procedures for each seed stock used or novel genotype generated. Describe any experiments used to assess the effect of a mutation and, where applicable, how potential secondary effects (e.g. second site T-DNA insertions, mosiacism, off-target gene editing) were examined.*
