# Supplementary material for: Plasma proteomic profiles of Alzheimer’s disease and neurodegeneration in African cohorts
Source: Nat Commun. 2026 Jul 1;17:8215. doi: 10.1038/s41467-026-74971-4 (PMC13462778; doi:10.1038/s41467-026-74971-4)
Supplement: Supplementary file 3 [file 41467_2026_74971_MOESM3_ESM.pdf]

nature portfolio

Peer Review File

Plasma proteomic profiles of Alzheimer's Disease and Neurodegeneration in African Cohorts

Corresponding Author: Dr Andréa Benedet

This file contains all reviewer reports in order by version, followed by all author rebuttals in order by version.

Version 0:

Reviewer comments:

Reviewer #1

(Remarks to the Author)
This study by Pola et al. investigates the relationship between plasma proteins as measured on the NULISA CNS assay and amyloid status as determined by plasma pTau217 levels in a Nigerian African community-based cohort focused on vascular disease and aging, VALIANT. The results are compared to a Canadian cohort, TRIAD, focused on aging and AD. The authors observe overall consistency in the canonical plasma biomarkers for AD in the VALIANT cohort compared to expected changes and the TRIAD cohort. Using different linear models, they identify proteins associated with amyloid and with cognitive impairment in the presence or absence of amyloid, and which of these proteins are affected by covariates associated with different medical co-morbidities and sex. They also examine levels by disease stage.

Overall the study is well conducted. The main contribution of the study rests primarily in the cohort examined, which is based in Africa where very few if any studies of this size have been conducted related to plasma AD biomarkers. It is therefore novel in this sense and the data if made available to the community could be valuable for better understanding differences across socioeconomic and ethnic populations. The study is largely descriptive, identifying proteins affected by various factors in this population. The comparison with the TRIAD cohort at the end does provide some data on marker overlap with another non-African population, but further exploration of potential differences with non-African study groups would provide more impact.

Major points
1. The figures are not legible, which makes it difficult to communicate and review the results of the study. This is a major issue with the paper that needs to be addressed.
2. The VALIANT cohort is focused on vascular disease, and presumably a proportion of cohort members have renal insufficiency that is known to affect pTau217 levels potentially leading to false positives in the amyloid-positive group. Are the authors able to address this potential confound?
3. Recent studies have examined plasma proteomic associations with amyloid and cognitive function in the presence and absence of amyloid using different proteomic platforms (for instance, see Afshar et al. Nat Aging 2025). The authors could explore further whether their findings are consistent with those observed from other platforms and cohorts.
4. Are there batch effects between the VALIANT data and the TRIAD data, and if so, how were they addressed?

Other points
1. Most studies have observed a larger difference in plasma pTau217 in PET-confirmed AD individuals compared to pTau181 or 231. However, this is not the case in this study. Can the authors explain this discrepancy?
2. A table with the demographics of the TRIAD comparator cohort would be helpful.
3. In different points in the manuscript the authors remark "The analyses were adjusted for age, sex and the individual average of the proteins." It is not clear what the last part means.
4. The authors do mention some caveats with the cohort, but it should be more strongly emphasized that most of the cohort is female with no amyloid, including in the cognitively impaired group.
5. UCHL1 has an opposite association with cognitive function depending on amyloid status. Can the authors explain?
6. Please describe what the "match" is for each panel in the legend for Figure 6 so the reader does not have to refer to Methods.
7. In the Abstract Background, I would suggest mentioning that most studies have been conducted in high-income countries, and not just in NHW populations.

8. In the Abstract Methods and introduction, I would suggest more explicitly describing what is different about the TRIAD cohort compared to the VALIANT cohort.
9. In the Abstract Conclusion and Discussion, remove “this pioneering study” and “this is a pioneering work.” This is subjective. If the authors would like to emphasize novelty, alternative language can be used such as “first study” if this is indeed the case.

Reviewer #2

(Remarks to the Author)
I read the work titled: Plasma proteomic profiles of Alzheimer’s Disease and Neurodegeneration in an Indigenous African Cohort: Data from VALIANT by Pola et al, with great interest.
The work is reporting proteomic profiling of Nigerian older adults of different grades of CI using NULISA platform and comparing them to a non African cohort.
It is interesting and needed to cover the gap in the global research landscape for dementia, however, I have some comments that may help improve the work:

The authors acknowledged the defect in proteomic research of dementia in Africa (compared to genetics) which is a true statement, but may be needs update given some recent works published from different African countries that could be useful addition, they may want to modify the statement accordingly.

I can see that they compared their findings with non African cohort to screen for similarities and differences, which is great, but I would be interested to see if they can find and compare to other African cohorts? This may be very helpful to have a dissection of profiles even within Africa given its ethnic diversity?

The cognitive profiles of subjects span across normal, MCI and AD, i wonder if they excluded other subtypes of dementia (Vascular, FTD, Mixed ..) to focus on AD? It would have been interesting to check and compare the proteomic profiles of different dementia phenotypes.

The study collected bio-samples, was there any chance to do genetic sequencing? If so, it would be very interesting to correlated proteomic profiles to genetic make up of the studied subjects.

On the other hand, can the authors provide more comprehensive environmental exposure data? This can be obtained through simple surveys, chemical analysis of bio samples or even correlating to address locations. This would be very interesting to study the correlation between the exposomse and proteomic profile

Reviewer #3

(Remarks to the Author)

Understanding the proteomic profiles in Alzheimer’s and aging across geogrphic, ancestral and socioeconomic distributions is of great importance to the scientific community. In this work, the authors provide the plasma proteomics profile from a community -based elderly cohort in Nigeria that includes cognitively normal and mildly impaired individuals. They provide amyloid-plaque, cognitive category and sex-based effects on the up or down regulated proteins and the related GO categories. Overall, the findings are novel based primarily on the population, but the lack of confirmatory AD biomarkers, imaging based measures of cerebrovascular burden does limit the meaning of the findings.

Below are some of the limitations that I have identified:

The authors rightly point out the high genetic diversity of African populations makes studies like these important, but it is important to note that this study is restricted to, presumably, a relatively limited population that likely has less genetic diversity and thus the results are limited to this cohort , as opposed to a broader African population.

Somewhat related, the method for determining the pT217 level for determine amyloid-plaque +/- is that this cut-off was not based on a population with a large % of african ancestry and was also based on a group with a substantially different % of CI than in the VALIANT cohort. Have the authors repeated their analyses based on different pT217 levels?

For the comparisons with the TRIAD cohort:

It would be helpful to provide a comparison of the % postiive for amyloid (based pT217) for each clinical category from the TRIAD cohort as well, to get a better sense of the population characteristics. Likewise, information on the comorbidities that were used in the VALIANT population. If there are substantial differences in medical comorbidities that were demonstrated to be associated with proteomic levels in the VALIANT population, it would be important to try to match for this in the TRIAD population so as not to attribute differences between the populations to differences in characteristics.

Another limitation of the comparison between the two cohorts is the substantial difference in the proportion of participants in

the TRIAD cohort that had cognitive impairment. This is likely to impact the proteomic profile of this group, despite controlling for age and pT217 levels

Version 1:

Reviewer comments:

Reviewer #1

(Remarks to the Author)
This is a revised study by Pola et al on the NULISA plasma proteomic findings in the VALIANT cohort. The authors have addressed some of the previous critiques, including now providing more legible figures and tables, although the supplementary tables are not available for review. On re-review of the manuscript, there remain a number of points that should be addressed:

1. Tables for the matches are now provided. Match 1 does not seem to be well matched by sex. Match 2 does not seem to be well matched by sex or pTau217 levels. Match 3 does not seem to be well matched by sex or pTau217 levels. If the goal is to compare to a non-African cohort while controlling for these variables, then ideally one would want to better match the variables. There do not seem to be any statistics presented to describe the match. It is recognized that it may not be possible to exactly match participants on these variables between the two cohorts, but if so, then this should be discussed as a caveat and some statistics provided to demonstrate where the matches have variables that differ between the cohorts.
2. It is recognized that the same platform is helpful to isolate cohort rather than assay effects when comparing to other populations. However, robustness of the assay itself is also a matter for replication. To this end, it would increase robustness of the findings if the authors could compare their observations to those of Pichet Binette et al Nat Neurosci 2024, for those proteins measured in common between NULISA and the Olink 3k platform employed in this study. A Venn diagram showing the overlap of proteins positively and negatively associated with amyloid, and which proteins are common or uniquely positively and negatively associated with amyloid in the VALIANT cohort, would be helpful. This is a cohort from a high-income Caucasian population, and the underlying platform technologies are similar but not identical.
3. In figures 2-4 panels B and C, it should be specified that the analysis is restricted to proteins only positively associated with the outcome (amyloid positivity/negativity, or cognitive impairment/unimpaired).
4. There should be discussion in the manuscript related to the unexpected finding observed with pTau217 levels compared to other pTau species.
5. In the legend for figures 2A, 3A, and 4A, if there are no darker points indicating FDR significance in the plots, then the legend should not describe darker points.
6. The legend in 5B-E is confusing to read. Would recommend reworking with punctuation for clarity.
7. In the legend for Figure 6, there needs to be a brief description of the goal of each match.

Reviewer #2

(Remarks to the Author)
I would like to thank the authors for their revisions and addressing my concerns. I look froward to future works by them that can address the un-addressable points in this work!

Reviewer #3

(Remarks to the Author)
I appreciate the detailed and comprehensive response by the authors, particularly the expanded information on the two cohorts used and the inclusion of an additional, albeit small, cohort from Tanzania.
I have no further comments.

Version 2:

Reviewer comments:

Reviewer #1

(Remarks to the Author)
The authors have addressed my comments. I have no further comments.

Open Access This Peer Review File is licensed under a Creative Commons Attribution 4.0 International License, which permits use, sharing, adaptation, distribution and reproduction in any medium or format, as long as you give appropriate credit to the original author(s) and the source, provide a link to the Creative Commons license, and indicate if changes were made.
In cases where reviewers are anonymous, credit should be given to 'Anonymous Referee' and the source.
The images or other third party material in this Peer Review File are included in the article's Creative Commons license, unless indicated otherwise in a credit line to the material. If material is not included in the article's Creative Commons license and your intended use is not permitted by statutory regulation or exceeds the permitted use, you will need to obtain permission directly from the copyright holder.
To view a copy of this license, visit https://creativecommons.org/licenses/by/4.0/

REVIEWER COMMENTS

We would like to start by expressing our gratitude to the reviewers and editors who devoted their time to provide meaningful contributions to our work. We have given our best trying to address all the suggestions below.

Reviewer #1 (Remarks to the Author):

This study by Pola et al. investigates the relationship between plasma proteins as measured on the NULISA CNS assay and amyloid status as determined by plasma pTau217 levels in a Nigerian African community-based cohort focused on vascular disease and aging, VALIANT. The results are compared to a Canadian cohort, TRIAD, focused on aging and AD. The authors observe overall consistency in the canonical plasma biomarkers for AD in the VALIANT cohort compared to expected changes and the TRIAD cohort. Using different linear models, they identify proteins associated with amyloid and with cognitive impairment in the presence or absence of amyloid, and which of these proteins are affected by covariates associated with different medical co-morbidities and sex. They also examine levels by disease stage.

Overall the study is well conducted. The main contribution of the study rests primarily in the cohort examined, which is based in Africa where very few if any studies of this size have been conducted related to plasma AD biomarkers. It is therefore novel in this sense and the data if made available to the community could be valuable for better understanding differences across socioeconomic and ethnic populations. The study is largely descriptive, identifying proteins affected by various factors in this population. The comparison with the TRIAD cohort at the end does provide some data on marker overlap with another non-African population, but further exploration of potential differences with non-African study groups would provide more impact.

Major points

1. The figures are not legible, which makes it difficult to communicate and review the results of the study. This is a major issue with the paper that needs to be addressed.

We thank the reviewer for pointing this out. We have updated the image quality for improved resolution.

2. The VALIANT cohort is focused on vascular disease, and presumably a proportion of cohort members have renal insufficiency that is known to affect pTau217 levels potentially leading to false positives in the amyloid-positive group. Are the authors able to address this potential confound?

We thank the reviewer's thoughtful comment and acknowledge their concern regarding renal insufficiency. We have assessed the available data and verified that almost all participants did not present with chronic kidney disease (CDK), while a very small number of participants (n= 7) did not have available information. Specifically, from the 7 participants with no available information, 6 were plasma pTau217 negative and only 1 positive. Given that only one individual was plasma pTau217 positive with unknown information on kidney function, we believe the potential impact of this individual on our results is negligible.

3. Recent studies have examined plasma proteomic associations with amyloid and cognitive function in the presence and absence of amyloid using different proteomic platforms (for instance, see Afshar et al. Nat Aging 2025). The authors could explore further whether their findings are consistent with those observed from other platforms and cohorts.

We thank the reviewer for bringing this to our attention. While recent studies such as Afshar et al. (Nat Aging, 2025) have identified plasma proteomic signatures associated with amyloid status using large-scale platforms (~7,000 proteins), direct comparison with our findings is inherently challenging by differences in proteomic coverage and platform design. Specifically, our study employed the NULISA CNS panel, which targets a much smaller (120 proteins), predefined set of CNS-relevant proteins. As such, overlap in individual proteins is restricted, although both approaches aim to capture biologically relevant pathways. However, our findings are consistent with those reported in other studies using targeted NULISA panels. Indeed, strong associations between amyloid positivity and core AD biomarkers, particularly phosphorylated tau species (p-tau217, p-tau231, p-tau181), as well as MAPT, GFAP, NEFL, and further inflammatory and synaptic markers could be highlighted. In the discussion section, we have included some relevant studies that have employed the NULISA platform (PMID: 40318118, PMID: 40678230, PMID: 39845736) and highlighted how future studies, leveraging larger panels might provide insights on additional proteins of interest, as in other proteomic studies (PMID: 40931114, PMID: 38977137). We have modified the discussion section as follows:

"*Our findings from the comparison of amyloid-positive and amyloid-negative individuals, reinforced the utility of p-tau biomarkers as the leading biomarkers in plasma (p-tau217, p-tau231, and p-tau181) in the context of amyloid pathology and AD and were consistent with other investigations conducted with the NULISA method*"

"*It is important to highlight that proteomic platforms including additional proteins might expand the identification of potentially novel proteins beyond established AD-related pathways. Therefore, future studies leveraging larger and more comprehensive panels may further expand the spectrum of amyloid-associated proteins, particularly in a context such the one in Africa*."

4. Are there batch effects between the VALIANT data and the TRIAD data, and if so, how were they addressed?

This is an important question. Indeed, the reagents used in both cohorts are slightly different versions of the assay. In the manuscript we have kept the data on their original format but as sensitivity analysis we have done a batch normalization as described below. No difference in results was observed after doing batch normalization. Furthermore, it is important to mention that the datasets have not been merged into a single set, so a batch normalization is not necessary for the analyses that we performed. Just for transparency, a batch normalization on the NULISA data is done through intensity normalization across cohorts/plates. This approach aligns the median NPQ value for each target across plates by adjusting plate-specific medians to a global median, thereby enabling combined analyses. Importantly, samples were randomized across plates to ensure comparable biological distributions, which is a key assumption of intensity normalization.

Other points

1. Most studies have observed a larger difference in plasma pTau217 in PET-confirmed AD individuals compared to pTau181 or 231. However, this is not the case in this study. Can the authors explain this discrepancy?

This is indeed an interesting finding that also caught our attention. While prior studies have often reported larger differences for plasma p-tau217 compared to p-tau181 or p-tau231 in PET-confirmed AD, our findings did not replicate this pattern. Despite that all p-tau markers were highly significant, in this dataset, p-tau181 and p-tau231 exhibited larger effect sizes (logFC) compared to p-tau217 in "amyloid-positive" versus "negative" individuals. One important aspect to mention is that we lack amyloid PET data, so a direct comparison with previous reports cannot be established. Another important aspect here is that the amyloid pathology status was proxied also by plasma p-tau217. Although this was quantified using another method, we cannot exclude some redundancy aspect in this association. Unfortunately, at this stage, we cannot offer a substantiated explanation for this observation. Notably, a similar pattern was also observed in the Tanzanian cohort during replication in this independent dataset, suggesting that this may represent a reproducible finding. Further studies are required to better understand its underlying mechanisms and implications.

2. A table with the demographics of the TRIAD comparator cohort would be helpful.

Yes, we agree with this reviewer that we should improve transparency here. As per request, we have included the Tables in "Supplementary Table 2" for "Match 1", "Match 2" and "Match 3", both for VALIANT and TRIAD cohort.

"*The demographic and biomarker information for the matched data ("Match 1", "Match 2" and "Match 3") are presented in* ***Supplementary Table 2.***"

Match 1

TRIAD cohort			
	**CU (N=192)**	**MCI (N=91)**	**Dem (N=32)**
**Sex (Male)**	63 (32.8%)	39 (42.9%)	26 (81.3%)
**Age**	69 (± 9.7)	71 (± 7.3)	75 (± 6.3)
**Years of education**	15 (± 3.4)	16 (± 3.7)	15 (± 5.0)
**Aß positive**	37 (19.3%)	47 (51.6%)	23 (71.9%)
**p-tau217 Simoa**	0.40 (± 0.45)	0.64 (± 0.55)	1.3 (± 0.94)

VALIANT cohort			
	**CU (N=192)**	**MCI (N=91)**	**Dem (N=32)**
**Sex (Male)**	90 (46.9%)	15 (16.5%)	8 (25.0%)
**Age**	67 (± 10)	73 (± 8.8)	77 (± 11)

**Years of education**	6.8 (± 4.8)	2.3 (± 3.3)	1.9 (± 3.7)
**Aß positive**	26 (13.5%)	19 (20.9%)	11 (34.4%)
**p-tau217 Simoa**	0.25 (± 0.14)	0.30 (± 0.24)	0.34 (± 0.27)

Match 2

TRIAD cohort			
	**CU (N=192)**	**MCI (N=91)**	**Dem (N=93)**
**Sex (Male)**	63 (32.8%)	39 (42.9%)	43 (46.2%)
**Age**	69 (± 9.7)	71 (± 7.3)	67 (± 9.2)
**Years of education**	15 (± 3.4)	16 (± 3.7)	14 (± 4.0)
**Aß positive**	37 (19.3%)	47 (51.6%)	58 (62.4%)
**p-tau217 Simoa**	0.40 (± 0.45)	0.64 (± 0.55)	1.1 (± 0.86)

VALIANT cohort			
	**CU (N=305)**	**MCI (N=51)**	**Dem (N=20)**
**Sex (Male)**	125 (41.0%)	14 (27.5%)	7 (35.0%)
**Age**	67 (± 11)	75 (± 9.1)	79 (± 10)
**Years of education**	5.8 (± 4.6)	2.3 (± 3.3)	2.4 (± 4.2)
**Aß positive**	62 (20.3%)	19 (37.3%)	10 (50.0%)
**p-tau217 Simoa**	0.28 (± 0.17)	0.38 (± 0.29)	0.42 (± 0.32)

Match 3

TRIAD cohort			
	**CU (N=192)**	**MCI (N=66)**	**Dem (N=32)**
**Sex (Male)**	129 (67.2%)	27 (40.9%)	9 (28.1%)
**Age**	69 (± 9.7)	71 (± 8.1)	73 (± 6.7)
**Years of education**	15 (± 3.4)	16 (± 3.6)	14 (± 4.2)
**Aß positive**	37 (19.3%)	22 (33.3%)	11 (34.4%)
**p-tau217 Simoa**	0.40 (± 0.45)	0.56 (± 0.56)	0.87 (± 0.89)

VALIANT cohort			
	**CU (N=192)**	**MCI (N=66)**	**Dem (N=32)**
**Sex (Male)**	101 (52.6%)	50 (75.8%)	24 (75.0%)
**Age**	67 (± 9.9)	72 (± 10)	77 (± 11)
**Years of education**	6.7 (± 4.8)	2.7 (± 3.7)	1.9 (± 3.7)
**Aß positive**	37 (19.3%)	22 (33.3%)	11 (34.4%)

**p-tau217 Simoa**	0.26 (± 0.15)	0.34 (± 0.27)	0.34 (± 0.27)

3. In different points in the manuscript the authors remark "The analyses were adjusted for age, sex and the individual average of the proteins." It is not clear what the last part means.

We sincerely thank the reviewer for bringing this to our attention and we will gladly provide more details. Trying to account for potential individual variability in plasma concentration levels, which could happen for different reasons, we averaged all protein quantifications within each individual and included in all analyses as a covariate. This approach has also been using in other proteomic studies (Binette et al. 2024, Imam et al. 2025).

We have included this in the Methods section (and cited other proteomic studies of AD employing such approach) to improve clarity to the reader:

"*The average of the protein quantification, within each individual, was included as covariate in the statistical models to account for interindividual variability in plasma concentration levels.*"

4. The authors do mention some caveats with the cohort, but it should be more strongly emphasized that most of the cohort is female with no amyloid, including in the cognitively impaired group.

We thank the reviewer for these comments, and we have made modifications as follows:

"*In this context, the low proportion of amyloid-positive individuals likely reflects the design of the study, based on a population-based cohort of community dwelling older adults aged > 50 years. As the prevalence of amyloid pathology is strongly age-dependent, the lower prevalence of p-tau217 positivity in the VALIANT cohort could be explained by the younger age of inclusion in the study.*"

"The higher proportion of CU participants, as compared to other AD research cohorts can also be potentially explained by the age criteria for inclusion in the study. Furthermore, differences in survival following dementia diagnosis across settings—potentially impacted by higher mortality in low- and middle-income countries—may contribute to variation in the proportion of diagnostic groups observed between research- and population-based studies between the cohorts here studied. As for what concerns the prevalence of women, this reflects both the design of the study and sociocultural characteristics of the Nigerian population. As participants were met in their homes, women were often more available to participate in the study than men, partly because many female participants were petty traders or housewives who did not work outside the home. "

5. UCHL1 has an opposite association with cognitive function depending on amyloid status. Can the authors explain?

We thank the reviewer for highlighting this important point.

In our dataset, UCHL1 levels did not significantly differ between cognitively unimpaired (CU) amyloid-negative and CU amyloid-positive individuals (t-test, n.s.), suggesting that, at the preclinical stage, UCHL1 remains within a physiological range regardless of amyloid status.

In contrast, we observed divergent patterns in CI individuals, with reduced UCHL1 levels in CI amyloid-negative participants and elevated levels in CI amyloid-positive participants relative to their respective CU counterparts. One possible interpretation is that distinct underlying pathological processes may contribute to these patterns. In CI amyloid-negative individuals, where the underlying etiology is likely heterogeneous and likely not driven by AD, reduced UCHL1 may reflect synaptic loss or reduced neuronal integrity. Conversely, in the presence of amyloid pathology (CI amyloid-positive), increased UCHL1 may reflect enhanced neuronal stress, compensatory upregulation, or increased release due to neurodegenerative processes.

Thus, the apparent opposite association may not reflect contradictory effects of UCHL1 per se, but rather distinct pathophysiological mechanisms in amyloid-negative versus amyloid-positive cognitive impairment.

We have included this in the Discussion section, since like the reviewer we find this point very interesting for discussion:

"*Interestingly, UCHL1 showed an opposite association with cognitive function depending on amyloid status. UCHL1 is a neuron-specific protein that in physiological conditions has been implicated in synaptic plasticity and proteostasis. In our results, UCHL1 levels did not differ between CU amyloid-negative and amyloid-positive individuals, suggesting physiological levels at the preclinical stage regardless of amyloid status. In contrast, divergent patterns were observed in cognitively impaired individuals, with decreased UCHL1 in CI amyloid-negative and increased levels in CI amyloid-positive relative to their respective CU groups, suggesting that different pathological processes may underlie these changes. Potentially, reduced UCHL1 might indicate synaptic loss in amyloid-negative impairment, while increased levels in amyloid-positive individuals might reflect neuronal stress or increased release associated with neurodegeneration.*"

6. Please describe what the "match" is for each panel in the legend for Figure 6 so the reader does not have to refer to Methods.

We thank the reviewer for this useful comment. We have included the "Match" description for each panel, to improve and facilitate the understanding of the findings.

7. In the Abstract Background, I would suggest mentioning that most studies have been conducted in high-income countries, and not just in NHW populations.

We thank the reviewer for the suggestion. We have included this in the manuscript as follows:

"*Yet, most of the studies have been focused on Non- Hispanic White (NHW) populations from high-income countries*"

8. In the Abstract Methods and introduction, I would suggest more explicitly describing what is different about the TRIAD cohort compared to the VALIANT cohort.

We thank the reviewer and we agree that Abstract, Methods and Introduction could benefit from additional information about the difference between the cohorts. Particularly, we described this cohort in more details in the Methods section, for better understanding to the reader.

In the Abstract:

"Additionally, participants from the Canadian TRIAD cohort, representing of a distinct population context were included for proteomic comparison."

In the Introduction:

"To disentangle population-specific risks and underlying pathological mechanisms, findings were then compared with those of participants from the Translational Biomarkers in Aging and Dementia (TRIAD) cohort, a deeply phenotyped Canadian longitudinal study with available NULISA data on plasma. This cohort served as a comparator, representing populations from high-income settings in which most biomarker research has been conducted so far, and providing a contrast to the Nigerian cohort."

In the Methods:

"For comparative analysis of the proteomic signatures between the two distinct population groups, participants from the Translational Biomarkers in Aging and Dementia cohort (TRIAD), a longitudinal imaging and biofluid cohort study of aging and AD in Canada, were included. TRIAD cohort consists of individuals who were recruited from a tertiary care memory clinic and cognitively unimpaired individuals who are adult volunteers from the community. These individuals are largely representative of the White population from a high-income country and detailed description of TRIAD's inclusion and exclusion criteria can be found elsewhere. TRIAD was approved by the MNI PET working committee and the Douglas Mental Health University Institute Research Ethics Board (MP-18-2017-157). Written informed consent was obtained for all participants"

"For TRIAD cohort sociodemographic data were collected from all participants, including sex (male and female), age and years of education."

"For TRIAD participants diagnosis consisted in cognitively unimpaired (CU), mild cognitive impairment (MCI), AD dementia, documented as previously described. Specifically, cognitively unimpaired individuals had showed no objective cognitive impairment, a CDR score of 0, and

were asked to report any subjective cognitive decline in a questionnaire provided during screening. Individuals with MCI showed relatively preserved activities of daily living, cognitive impairment and a CDR score of 0.5. Patients with Mild-to-moderate Alzheimer's clinical syndrome dementia had a CDR score between 0.5 and 2 and met the National Institute on Aging—Alzheimer's Association (NIA-AA) criteria for probable AD determined by a dementia specialist."

"For TRIAD, inclusion criteria for all participants are the ability to speak English or French, good general health (no diseases expected to interfere with study participation over time), absence of claustrophobia, and adequate visual and auditory capacities to follow neuropsychologic evaluation. Additionally, participants were excluded if they had inadequately treated conditions, active substance abuse, recent head trauma, or major surgery, or if they had MRI/PET safety contraindication."

9. In the Abstract Conclusion and Discussion, remove "this pioneering study" and "this is a pioneering work." This is subjective. If the authors would like to emphasize novelty, alternative language can be used such as "first study" if this is indeed the case.

We thank the reviewer for pointing this out. We have replaced as follows:

"This study, investigating plasma biomarker profiles in an indigenous African cohort, identified similar patterns of elevated core AD biomarkers in p-tau217-positive individuals as previously described in other populations."

"This study leverages a unique cohort and constitutes the largest sample size of any Alzheimer's disease biomarker study conducted in Africa to date, allowing for the investigation of proteomic profiles in this setting. The nature of the study provides insights on the AD biomarker scenario in underrepresented groups, like the ones in Africa."

Reviewer #2 (Remarks to the Author):

I read the work titled: Plasma proteomic profiles of Alzheimer's Disease and Neurodegeneration in an Indigenous African Cohort: Data from VALIANT by Pola et al, with great interest.
The work is reporting proteomic profiling of Nigerian older adults of different grades of CI using NULISA platform and comparing them to a non-African cohort.
It is interesting and needed to cover the gap in the global research landscape for dementia, however, I have some comments that may help improve the work:

The authors acknowledged the defect in proteomic research of dementia in Africa (compared to genetics) which is a true statement, but may be needs update given some recent works published from different African countries that could be useful addition, they may want to modify the statement accordingly.

We thank the reviewer for this comment, and we have updated the manuscript with additional interesting studies, one of which have been published after the initial submission of this work. As previously stated, several recent studies from African cohorts have evaluated core plasma biomarkers of Alzheimer's disease (e.g., Aβ42/40, p-tau species, GFAP, NfL). In addition to that, recent work has begun to address this gap (PMID: 41653107, 37468915)
We have revised the statement to reflect the distinction between core-biomarkers studies and proteomic studies more precisely, for better clarity to the reader.

"*To date, biofluid biomarker studies in African populations have been restricted to core AD markers (GFAP, NfL and p-tau species) and a narrow set of inflammatory markers, typically evaluated in small cohorts, limiting their statistical robustness and generalizability. While emerging studies in African-ancestry cohorts are beginning to capture ancestry-specific biology, addressing a critical gap at both the proteomic and metabolomic levels, biomarker research in these populations remain limited"*

I can see that they compared their findings with non-African cohort to screen for similarities and differences, which is great, but I would be interested to see if they can find and compare to other African cohorts? This may be very helpful to have a dissection of profiles even within Africa given its ethnic diversity?

We thank the reviewer for this insightful comment and fully agree that comparisons with other African cohorts would substantially strengthen the interpretation of our findings, particularly given the considerable genetic and ethnic diversity across the continent.

At present, proteomic data in African-ancestry populations remain scarce. Ongoing initiatives, such as the Recruitment and Retention for Alzheimer's Disease Diversity Genetic Cohorts in the ADSP (READD-ADSP), aim to expand biomarker research in African-ancestry and Hispanic/Latinx populations and are expected to generate proteomic data in the near future.

In an effort to address this important point within the revision timeframe, we sought to expand our analyses by incorporating data from an additional African cohort. Specifically, we accessed and analyzed data from a study conducted in the rural Hai district of northern Tanzania, comprising 196 participants with available NULISA, clinical, and sociodemographic data. To our knowledge, this represents the only other African cohort currently available with comparable proteomic measurements.

These analyses have now been incorporated and discussed in detail in the revised manuscript. Briefly, in both the Tanzanian cohort and the VALIANT cohort, linear models were applied to assess differential protein expression between amyloid-positive and amyloid-negative participants. Of note, the Tanzanian cohort, similarly to the VALIANT cohort, did not have amyloid pathology indexed by PET or CSF nor plasma p-tau217 quantified by another platform. For this reason, the cutoff of > 0.42 used for VALIANT was not applicable for this cohort. Therefore, trying to replicate the models used for VALIANT, a cutoff for amyloid positivity was then defined as values exceeding the 70th percentile of p-tau217 (NULISA) in the cognitively unimpaired group. The same cutoff strategy was then applied to the VALIANT cohort to enable

comparison. The analyses were adjusted for age, sex and the individual average of the proteins as previously. Importantly, in both cohorts several proteins such as p-tau217, p-tau231, p-tau181, MAPT, NEFL, NRGN, NPTX2, CX3CL1 and FABP3 were upregulated in the amyloid-positive group, while proteins such as HTT, PARK7 and S100A12 were upregulated in the amyloid-negative group. Such proteins largely overlapped with the proteins identified in the original comparison of amyloid-positive and amyloid-negative in the VALIANT cohort.

To fully address the question of the reviewer and to expand and complement our research question, we have included such results in the manuscript, which we believe provide a valuable addition to the work. We have updated as follows throughout the manuscript in Abstract, Introduction, Results, Discussion and Methods sections.

Abstract:

"Participants from a study conducted in Tanzania (n=196) and with available NULISA data were included to replicate the analyses for replication."

"Findings were largely consistent amongst the African cohorts."

Introduction:

"The primary analyses were further replicated in an independent indigenous African cohort from northern Tanzania."

Results:

Participants and biomarker characteristics.

*"To replicate the main analysis, were included 196 participants from the Tanzanian study, of which 196 had available blood-based biomarker data together with age, sex and sociodemographic data; 131 participants had available information on education level (66.5%). The average age of this cohort was 80.3 (SD ± 10.4) years, 34.1% were male, and 61.1% and 40.8% had cognitive impairment as mild cognitive impairment (MCI) or dementia, respectively (**Supplementary Table 1**)."*

*"Importantly, when comparing amyloid-positive with amyloid-negative individuals in both VALIANT and the Tanzanian cohorts, proteins such as p-tau217, p-tau231, p-tau181, MAPT, NEFL, NRGN, NPTX2, CX3CL1 and FABP3 were consistently found upregulated in amyloid-positive compared to amyloid-negative participants, while proteins such as HTT, PARK7 and S100A12 were consistently downregulated. Detailed reports related to the Tanzanian analyses were included in (**Supplementary Figure 2A-B and Supplementary Table** 7). Furthermore, the use of the two different cutoffs for amyloid positivity within the VALIANT cohort (Simoa p-tau217 cutoff or as defined by NULISA p-tau217 values exceeding the 70th percentile in cognitively unimpaired individuals) yielded similar results; proteins including p-tau231, p-tau181, p-tau217, MAPT, FABP3, KLK6 and FABP3 were found elevated in amyloid-positive individuals (P_{adj} < 0.05), while IL6R, TIMP3, HTT, CD40LG , ANXA5, BDNF had lower levels in comparison with*

amyloid-negative individuals (Padj < 0.05) ***(Supplementary Figure 2C-D, Supplementary Table 8****).”*

Discussion:

“Such findings were consistently observed in all cohorts, but more similarities in protein expression were found among the African cohorts as compared with the North American cohort.”

“Such findings were observed in both African cohorts, with p-tau217, p-tau181 and p-tau231 appearing as the most significant biomarkers.”

Methods:

Study design and participants

“This study included participants from Nigeria (VALIANT cohort), Tanzania (Hai Dementia study) and Canada (TRIAD cohort).”

“To replicate the main analysis, participants from the rural Hai district of northern Tanzania were included. The cohort consists of community-dwelling adults aged 65 years and over, recruited from six villages within Hai and selected to be representative of upland and lowland areas and different economic status. The study was a community-based door to door dementia prevalence study using the 10/66 protocol. Dementia diagnosis was made by consensus following a detailed interview and based on the DSM-IV criteria. Mild cognitive impairment (MCI) was defined by Petersen criteria. More detailed information about the recruitment process for this cohort has been previously reported. Ethical approval for this study was granted by the National Institute for Medical Research, Tanzania. Informed consent for each participant was obtained after verbal explanation of the information sheet from a Tanzanian medical officer or nurse. Individuals unable to give written consent due to illiteracy or severe visual impairment indicated consent using a thumbprint. Where participants were deemed to lack capacity to consent due to cognitive impairment, assent was taken from a close relative provided the participant actively demonstrated willingness to participate. Blood samples were obtained at a home visit, transported on ice within two hours of being taken, spun into aliquots and stored in a -80 freezer at Kilimanjaro Christian Research Institute before transport for proteomic analysis. 196 participants had available blood-based biomarkers and sociodemographic data.”

Sociodemographic data

“For the Tanzanian cohort, sociodemographic data were collected from all participants, including sex (male and female) and age. Information about education were available for 131 participants (66.5%) and categorized as no education at all, 4 years or less of education, more than 4 years of education. Education was coded as 1 for no education and as 0 for some primary education or higher level.”

Cognitive assessment

"For the Tanzanian participants, cognition was assessed at baseline using the IDEA cognitive screen, as described previously"

Blood-based Biomarker Analysis

"Biomarker data were obtained for 949 study participants from VALIANT, 196 study participants from Tanzanian cohort and 376 study participants for TRIAD cohort."

"For the Tanzanian cohort, 10 milliliters of whole blood samples were also collected in EDTA tubes and transported in coolers (ice-boxes) to the laboratory at the Kilimanjaro Clinical Research Institute (KCRI) within ~3 hours after collection. They were similarly separated into three fractions (plasma, buffy coat and RBC) after centrifugation at 4◦C, 4000 × g for 10 min and stored at -80oC at KCRI. One ml duplicates of the original plasma samples were eventually received at the Campus for Ageing and Vitality, Newcastle upon Tyne for further analyses. Two hundred and fifty µl aliquots of these plasma samples were then sent to Gothenburg from Newcastle for the NULISA analysis. "

"Plasma samples were shipped frozen on dry ice to and analysed at the Department of Psychiatry and Neurochemistry, University of Gothenburg, Gothenburg, Sweden. All the biomarker analyses were done blinded to diagnosis and sample information."

"The three cohorts were also analysed on the ARGO-HT platform, with the NULISA^{TM} CNS-disease assay, targeting neurodegenerative disease–related targets, inflammation and immune response–related cytokines and chemokines at the Department of Psychiatry and Neurochemistry, University of Gothenburg as described previously. To allow for comparison with the Tanzanian cohort, another cutoff was employed, where cutoff positivity was defined as values exceeding the 70th percentile of p-tau217 (NULISA) among cognitively unimpaired participants. Such approach was derived by prior meta-analysis, which reported that approximately 30% of the cognitively unimpaired individuals are amyloid PET-positive between 55-59 years of age and applied to both the VALIANT and Tanzanian cohorts. The rationale of use of such cutoff was due to the unavailability of another standard of truth or biomarker in common between the African cohorts."

Statistical analysis

"For both Tanzanian and VALIANT cohort, linear models evaluated the differential protein expression between amyloid-positive vs. amyloid-negative (with cutoff positivity defined as values exceeding the 70th percentile of p-tau217 (NULISA) among cognitively unimpaired participants, as previously described) for which the target proteins were set as the dependent variable and the group as the predictor variable. The analyses were adjusted for age, sex and the individual average of the proteins. Volcano plots reported, using colour schemes, the proteins that were statistically significant when contrasting groups either nominally or after false discovery rate (FDR) correction for multiple comparisons. Venn Diagrams were employed to visualize the differences in proteomic signatures in the two cohorts, employing the 'InteractiVenn' tool"

Additionally, we addressed the importance of conducting other studies in the context of Africa, highlighting that our research represents an initial step and encouraging further studies and evaluation by other researchers given its relevance in this context.

"*Finally, given the substantial ethnic and sociodemographic heterogeneity across the African continent, it is important to note that this study was conducted in a representative population from Nigeria, and its findings may not generalize fully to all African regions. To further expand the understanding of the proteomic findings, we have explored proteomic profiles from a study conducted in rural Hai district of northern Tanzania. Building on this initial endeavor, studies in other African cohorts, as well as genetic profiling will be required in the future, to gain more in-depth insights into the AD proteomic scenario in the African population."*

"Additionally, given the substantial ethnic and sociodemographic heterogeneity across the African continent, it is important to note that this study was conducted in representative populations from Nigeria and rural Tanzania, and findings may not generalize fully to all African regions. Finally, further proteomic profiling in other African cohorts, as well as genetic profiling, will be required in the future, to gain more in-depth insights into the AD proteomic scenario in the African population."

The cognitive profiles of subjects span across normal, MCI and AD, i wonder if they excluded other subtypes of dementia (Vascular, FTD, Mixed ..) to focus on AD? It would have been interesting to check and compare the proteomic profiles of different dementia phenotypes.

Regarding clinical diagnosis, participants were categorized into Cognitively Unimpaired (CU), MCI and Dementia according to NIA-AA Criteria, Peterson's criteria and the DSM IV (McKhann et al. 2011; Petersen et al. 2001; American Psychiatric Association, 1994) by the independent evaluation of two neurologists (OO and RA), who reached a consensus when discordance was found. These evaluations were supported by cognitive and functional assessments. However, dementia subtyping was not performed for this cohort.

The study collected bio-samples, was there any chance to do genetic sequencing? If so, it would be very interesting to correlated proteomic profiles to genetic make up of the studied subjects.

We thank the reviewer for this important comment. Genetic sequencing was not performed in the present study. We agree that the inclusion of genomic data would provide valuable mechanistic insights and enable the investigation of genetic modifiers of biomarker associations. We are planning to perform genetic sequencing in this cohort in the future, pending the availability of funding. Given the sample size, comprehensive genomic analyses will require substantial additional resources, and we are actively pursuing funding to support this work.

However, we agree with the reviewer that integrating genomic profiling in future work would be highly informative and we aim at expanding this in the future and we have included this as a future direction of the study.

"Finally, further proteomic profiling in other African cohorts, as well as genetic profiling, will be required in the future, to gain more in-depth insights into the AD proteomic scenario in the African population."

On the other hand, can the authors provide more comprehensive environmental exposure data? This can be obtained through simple surveys, chemical analysis of bio samples or even correlating to address locations. This would be very interesting to study the correlation between the exposome and proteomic profile

We thank the reviewer for this question, and we agree that the environmental exposure data could provide interesting insights. From the available data we have available for this cohort, we examined whether global proteomic profiles differed according to type of domicile (rural areas or urban areas). Principal Component Analysis (PCA) was performed, and the first two principal components were visualized and statistically tested. Visual inspection of the PCA plot showed no clear separation between the two domicile groups. A Welch t-test comparing PC1 between groups was not statistically significant ($p = 0.68$), and ANOVA testing PC1 and PC2 jointly was also not significant ($p = 0.11$). Overall, these results indicate no evidence of a global proteomic difference between domicile types.

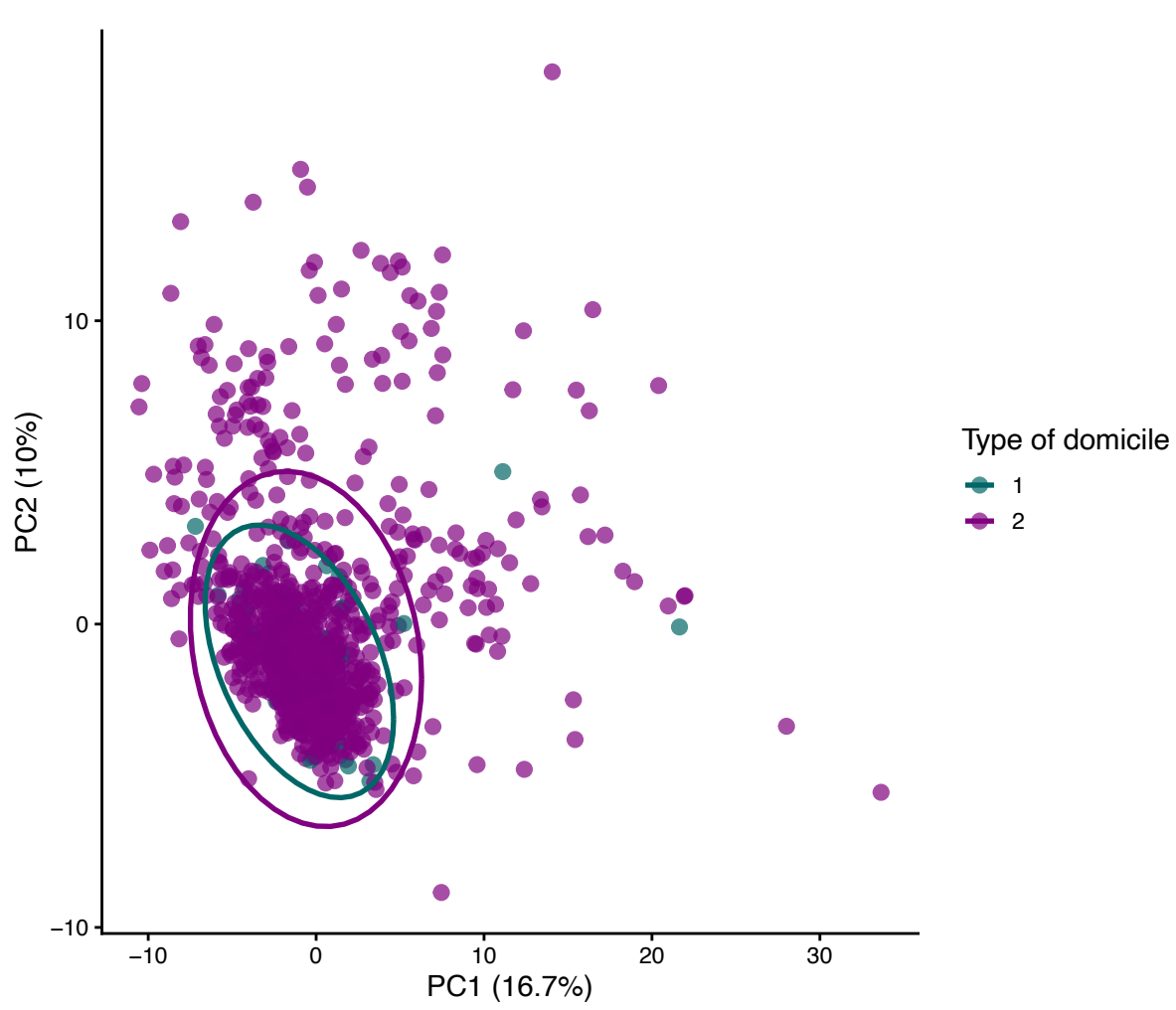

Reviewer #3 (Remarks to the Author):

Understanding the proteomic profiles in Alzheimer's and aging across geographic, ancestral and socioeconomic distributions is of great importance to the scientific community. In this work, the

authors provide the plasma proteomics profile from a community -based elderly cohort in Nigeria that includes cognitively normal and mildly impaired individuals. They provide amyloid-plaque, cognitive category and sex-based effects on the up or down regulated proteins and the related GO categories. Overall, the findings are novel based primarily on the population, but the lack of confirmatory AD biomarkers, imaging-based measures of cerebrovascular burden does limit the meaning of the findings.

Below are some of the limitations that I have identified:

The authors rightly point out the high genetic diversity of African populations makes studies like these important, but it is important to note that this study is restricted to, presumably, a relatively limited population that likely has less genetic diversity and thus the results are limited to this cohort , as opposed to a broader African population.

We thank the reviewer for this insightful comment and fully agree that comparisons with other African cohorts would substantially strengthen the interpretation of our findings, particularly given the considerable genetic and ethnic diversity across the continent.

At present, proteomic data in African-ancestry populations remain scarce. Ongoing initiatives, such as the Recruitment and Retention for Alzheimer's Disease Diversity Genetic Cohorts in the ADSP (READD-ADSP), aim to expand biomarker research in African-ancestry and Hispanic/Latinx populations and are expected to generate proteomic data in the near future.

In an effort to address this important point within the revision timeframe, we sought to expand our analyses by incorporating data from an additional African cohort. Specifically, we accessed and analyzed data from a study conducted in the rural Hai district of northern Tanzania, comprising 196 participants with available NULISA, clinical, and sociodemographic data. To our knowledge, this represents the only other African cohort currently available with comparable proteomic measurements.

These analyses have now been incorporated and discussed in detail in the revised manuscript. Briefly, in both the Tanzanian cohort and the VALIANT cohort, linear models were applied to assess differential protein expression between amyloid-positive and amyloid-negative participants. Of note, the Tanzanian cohort, similarly to the VALIANT cohort, did not have amyloid pathology indexed by PET or CSF nor plasma p-tau217 quantified by another platform. For this reason, the cutoff of > 0.42 used for VALIANT was not applicable for this cohort. Therefore, trying to replicate the models used for VALIANT, a cutoff for amyloid positivity was then defined as values exceeding the 70th percentile of p-tau217 (NULISA) in the cognitively unimpaired group. The same cutoff strategy was then applied to the VALIANT cohort to enable comparison. The analyses were adjusted for age, sex and the individual average of the proteins as previously. Importantly, in both cohorts several proteins such as p-tau217, p-tau231, p-tau181, MAPT, NEFL, NRGN, NPTX2, CX3CL1 and FABP3 were upregulated in the amyloid-positive group, while proteins such as HTT, PARK7 and S100A12 were upregulated in the amyloid-negative group. Such proteins largely overlapped with the proteins identified in the original comparison of amyloid-positive and amyloid-negative in the VALIANT cohort.

To fully address the question of the reviewer and to expand and complement our research question, we have included such results in the manuscript, which we believe provide a valuable addition to the work. We have updated as follows throughout the manuscript in Abstract, Introduction, Results, Discussion and Methods sections.

Abstract:

"Participants from a study conducted in Tanzania (n=196) and with available NULISA data were included to replicate the analyses for replication."

"Findings were largely consistent amongst the African cohorts."

Introduction:

"The primary analyses were further replicated in an independent indigenous African cohort from northern Tanzania."

Results:

Participants and biomarker characteristics.

*"To replicate the main analysis, were included 196 participants from the Tanzanian study, of which 196 had available blood-based biomarker data together with age, sex and sociodemographic data; 131 participants had available information on education level (66.5%). The average age of this cohort was 80.3 (SD ± 10.4) years, 34.1% were male, and 61.1% and 40.8% had cognitive impairment as mild cognitive impairment (MCI) or dementia, respectively (**Supplementary Table 1**)."*

*"Importantly, when comparing amyloid-positive with amyloid-negative individuals in both VALIANT and the Tanzanian cohorts, proteins such as p-tau217, p-tau231, p-tau181, MAPT, NEFL, NRGN, NPTX2, CX3CL1 and FABP3 were consistently found upregulated in amyloid-positive compared to amyloid-negative participants, while proteins such as HTT, PARK7 and S100A12 were consistently downregulated. Detailed reports related to the Tanzanian analyses were included in (**Supplementary Figure 2A-B and Supplementary Table 7**). Furthermore, the use of the two different cutoffs for amyloid positivity within the VALIANT cohort (Simoa p-tau217 cutoff or as defined by NULISA p-tau217 values exceeding the 70th percentile in cognitively unimpaired individuals) yielded similar results; proteins including p-tau231, p-tau181, p-tau217, MAPT, FABP3, KLK6 and FABP3 were found elevated in amyloid-positive individuals (Padj < 0.05), while IL6R, TIMP3, HTT, CD40LG , ANXA5, BDNF had lower levels in comparison with amyloid-negative individuals (Padj < 0.05) **(Supplementary Figure 2C-D, Supplementary Table 8**)."*

Discussion:

"Such findings were consistently observed in all cohorts, but more similarities in protein expression were found among the African cohorts as compared with the North American cohort."

"Such findings were observed in both African cohorts, with p-tau217, p-tau181 and p-tau231 appearing as the most significant biomarkers."

Methods:

Study design and participants

"This study included participants from Nigeria (VALIANT cohort), Tanzania (Hai Dementia study) and Canada (TRIAD cohort)."

"To replicate the main analysis, participants from the rural Hai district of northern Tanzania were included. The cohort consists of community-dwelling adults aged 65 years and over, recruited from six villages within Hai and selected to be representative of upland and lowland areas and different economic status. The study was a community-based door to door dementia prevalence study using the 10/66 protocol. Dementia diagnosis was made by consensus following a detailed interview and based on the DSM-IV criteria. Mild cognitive impairment (MCI) was defined by Petersen criteria. More detailed information about the recruitment process for this cohort has been previously reported. Ethical approval for this study was granted by the National Institute for Medical Research, Tanzania. Informed consent for each participant was obtained after verbal explanation of the information sheet from a Tanzanian medical officer or nurse. Individuals unable to give written consent due to illiteracy or severe visual impairment indicated consent using a thumbprint. Where participants were deemed to lack capacity to consent due to cognitive impairment, assent was taken from a close relative provided the participant actively demonstrated willingness to participate. Blood samples were obtained at a home visit, transported on ice within two hours of being taken, spun into aliquots and stored in a -80 freezer at Kilimanjaro Christian Research Institute before transport for proteomic analysis. 196 participants had available blood-based biomarkers and sociodemographic data."

Sociodemographic data

"For the Tanzanian cohort, sociodemographic data were collected from all participants, including sex (male and female) and age. Information about education were available for 131 participants (66.5%) and categorized as no education at all, 4 years or less of education, more than 4 years of education. Education was coded as 1 for no education and as 0 for some primary education or higher level."

Cognitive assessment

"For the Tanzanian participants, cognition was assessed at baseline using the IDEA cognitive screen, as described previously"

Blood-based Biomarker Analysis

"Biomarker data were obtained for 949 study participants from VALIANT, 196 study participants from Tanzanian cohort and 376 study participants for TRIAD cohort."

"For the Tanzanian cohort, 10 milliliters of whole blood samples were also collected in EDTA tubes and transported in coolers (ice-boxes) to the laboratory at the Kilimanjaro Clinical Research Institute (KCRI) within ~3 hours after collection. They were similarly separated into three fractions (plasma, buffy coat and RBC) after centrifugation at 4◦C, 4000 × g for 10 min and stored at -80oC at KCRI. One ml duplicates of the original plasma samples were eventually received at the Campus for Ageing and Vitality, Newcastle upon Tyne for further analyses. Two hundred and fifty µl aliquots of these plasma samples were then sent to Gothenburg from Newcastle for the NULISA analysis. "

"Plasma samples were shipped frozen on dry ice to and analysed at the Department of Psychiatry and Neurochemistry, University of Gothenburg, Gothenburg, Sweden. All the biomarker analyses were done blinded to diagnosis and sample information."

"The three cohorts were also analysed on the ARGO-HT platform, with the NULISA™ CNS-disease assay, targeting neurodegenerative disease–related targets, inflammation and immune response–related cytokines and chemokines at the Department of Psychiatry and Neurochemistry, University of Gothenburg as described previously. To allow for comparison with the Tanzanian cohort, another cutoff was employed, where cutoff positivity was defined as values exceeding the 70th percentile of p-tau217 (NULISA) among cognitively unimpaired participants. Such approach was derived by prior meta-analysis, which reported that approximately 30% of the cognitively unimpaired individuals are amyloid PET-positive between 55-59 years of age and applied to both the VALIANT and Tanzanian cohorts. The rationale of use of such cutoff was due to the unavailability of another standard of truth or biomarker in common between the African cohorts."

Statistical analysis

"For both Tanzanian and VALIANT cohort, linear models evaluated the differential protein expression between amyloid-positive vs. amyloid-negative (with cutoff positivity defined as values exceeding the 70th percentile of p-tau217 (NULISA) among cognitively unimpaired participants, as previously described) for which the target proteins were set as the dependent variable and the group as the predictor variable. The analyses were adjusted for age, sex and the individual average of the proteins. Volcano plots reported, using colour schemes, the proteins that were statistically significant when contrasting groups either nominally or after false discovery rate (FDR) correction for multiple comparisons. Venn Diagrams were employed to visualize the differences in proteomic signatures in the two cohorts, employing the 'InteractiVenn' tool"

Somewhat related, the method for determining the pT217 level for determine amyloid-plaque +/- is that this cut-off was not based on a population with a large % of African ancestry and was also based on a group with a substantially different % of CI than in the VALIANT cohort. Have the authors repeated their analyses based on different pT217 levels?

We acknowledge the reviewer's thoughtful comment and thank them for it. Indeed, the lack of a standard of truth for the assessment of amyloid pathology is a limitation of the study. Given the still recent research on African populations, it is difficult to establish what would be a good cutoff to be applied here, as well as to have a good sense of what are reasonable to expect in terms of disease prevalence. Therefore, our obvious approach was to use a known published cutoff for the method, although not ideal considering the large differences between the populations where the cutoff was derived from and the one where it is applied to.

When working on the rebuttal of this manuscript, we have been able to incorporate data from another African cohort and therefore partially replicate the analysis. This new cohort only had plasma biomarker data from the NULISA platform, so we adopted another strategy to derive the p-tau217 cutoff for amyloid pathology status. Based on the literature, which has shown that approximately 30% of the individuals without cognitive impairment have amyloid pathology between 55-59yo (PMID: 25988462), we defined the 70th percentile of p-tau217 in the CU individuals in the African cohorts (independently) to be the cutoff for amyloid positivity status. What we observed with this new cutoff is that a higher proportion of the VALIANT participants are considered to have amyloid pathology, although the biomarkers identified as significant in the initial analysis remained significant using this approach. Indeed, proteins including p-tau231, p-tau181, p-tau217, MAPT, FABP3, KLK6 and FABP3 were found elevated in amyloid-positive individuals ($P_{adj} < 0.05$), while IL6R, TIMP3, HTT, CD40LG , ANXA5, BDNF had lower levels in comparison with amyloid-negative individuals ($P_{adj} < 0.05$). We have included this in the manuscript as follows:

"*Importantly, when comparing amyloid-positive with amyloid-negative individuals in both VALIANT and Tanzanian cohorts, proteins such as p-tau217, p-tau231, p-tau181, MAPT, NEFL, NRGN, NPTX2, CX3CL1 and FABP3 were consistently found upregulated in amyloid-positive compared to amyloid-negative participants, while proteins such as HTT, PARK7 and S100A12 were consistently downregulated. Detailed reports related to the Tanzanian analyses were included in (**Supplementary Figure 2A-B and Supplementary Table** 7). Furthermore, the use of the two different cutoffs for amyloid positivity within the VALIANT cohort (Simoa p-tau217 cutoff or as defined by NULISA p-tau217 values exceeding the 70th percentile in cognitively unimpaired individuals) yielded similar results; proteins including p-tau231, p-tau181, p-tau217, MAPT, FABP3, KLK6 and FABP3 were found elevated in amyloid-positive individuals (Padj < 0.05), while IL6R, TIMP3, HTT, CD40LG , ANXA5, BDNF had lower levels in comparison with amyloid-negative individuals (Padj < 0.05) (**Supplementary Figure 2C-D, Supplementary Table 8**..*"

Although we understand that, again, the prevalence of amyloid pathology was estimated with data from research cohorts from well-educated high-income countries with a potential selection bias towards higher positivity rates as compared with a population-based studies -such as the African cohorts used here-, we believe this bias would be balanced by the higher prevalence of dementia and increased risk factors that the low-and-middle income countries face (PMID: 34569714, 38696263).

For the comparisons with the TRIAD cohort:

It would be helpful to provide a comparison of the % positive for amyloid (based pT217) for each clinical category from the TRIAD cohort as well, to get a better sense of the population characteristics. Likewise, information on the comorbidities that were used in the VALIANT population. If there are substantial differences in medical comorbidities that were demonstrated to be associated with proteomic levels in the VALIANT population, it would be important to try to match for this in the TRIAD population so as not to attribute differences between the populations to differences in characteristics.

We thank this reviewer for bringing this up. We have included the Tables in "**Supplementary Table 1**" to better characterize the TRIAD cohort as a whole, as well as the subsamples used for "Match 1", "Match 2" and "Match 3".

"*The demographic and biomarker information for the matched data ("Match 1", "Match 2" and "Match 3") are presented in **Supplementary Table 1.***"

Regarding the question about comorbidities, even though we have not been able to statistically evaluate whether there is a higher prevalence of certain types of comorbidities in one cohort or another, we presume based on the knowledge we have from the cohorts and from the literature (PMID: 39096926, 36968620) that considerable differences are expected in terms of comorbidities and risk factors between these two groups of cohorts. While TRIAD represents a HIC and is a research-based cohort where several health conditions were part of the exclusion criteria of the study (see description below), the African cohorts represent populations from LMIC and are population-based studies, where the inclusion criteria for the studies were not so stringent. We totally agree that it would be incredibly interesting to be able to also match the populations based on their medical comorbidities but this is unfeasible at the moment. Such approach would need a detailed registration and assessment of the different conditions and a very large sample size from both cohorts to enable statistical inferences. Furthermore, we would like to point out that our main focuses of interest with this initial work are to characterize and present the data from the Nigerian cohort as well as to establish a comparison with another cohort which represents the context of which most of the biomarker research has been done with so far. We are interested in showing where the data agrees, which is largely represented by the core AD biomarkers, but also where the disagreement lies. We have no power or knowledge at this moment to defend that the disagreement in the results is due to genetic background. Perhaps we could dare to say that comorbidities are driving these differences, and, on our perspective, this is interesting enough and highlights the need for future research on this matter.

Following is further information about the TRIAD cohort. In the Translational Biomarkers in Aging and Dementia (TRIAD) cohort, individuals undergo extensive clinical and neuropsychological assessments to confirm their health status. The cognitively unimpaired population consists of participants who do not exhibit any cognitive complaint and do not meet the criteria for mild cognitive impairment or any form of dementia (McKhann et al, 2011). Furthermore, participants with these medical conditions are excluded: clinically significant hypertension, anemia, liver or kidney disease, neurodevelopmental cognitive syndromes, medical

history of psychiatric or neurological conditions, history within 2 years prior to screening, or current evidence of, a psychotic disorder, a major depressive disorder, alcoholism or drug dependency/abuse, history of cancer, significant multiple and/or severe allergies, history of endocrine, gastrointestinal, cardiovascular, hematological, hepatic, immunological, renal, respiratory, genitourinary or major neurological abnormality or disease, which is not adequately controlled through a stable medication regimen, positive test result for hepatitis B surface antigen, hepatitis C antibodies, or human immunodeficiency virus (HIV), and if they had a major surgery or donated/lost 1 unit of blood (approx.. 500 mL) within 4 weeks prior to screening. Are also excluded participants with contra-indication to MRI or PET: such as pacemaker, cardiac defibrillator, implanted material with electric or magnetic system, vascular clips or mechanical heart valve, cochlear implant or claustrophobia. Patients in regular use of vitamin E at greater than daily dose of 600 and users of systemic or inhalation corticosteroids, anti-coagulants such as warfarin, ticlopidine, clopidogrel, or similar are excluded along with those individuals treated with any inflammatory or chronic pain condition that necessitates regular use of opiates (e.g., oxycodone, hydrocodone, tramadol, meperidine, hydromorphone). The use of commercially available memantine or acetyl-cholinesterase inhibitor including donezepil, rivastigmine, or galantamine can be allowed. We have included the appropriate references in the manuscript.

Another limitation of the comparison between the two cohorts is the substantial difference in the proportion of participants in the TRIAD cohort that had cognitive impairment. This is likely to impact the proteomic profile of this group, despite controlling for age and pT217 levels

We thank the reviewer for the insights, and we will be happy to clarify some aspects.

The comparative analysis consisted of 3 different subset “Matches” between cohorts:

- “Match 1” included 315 participants, matched by age, sex and clinical status (cognitively unimpaired (CU), MCI, Dementia)
- “Match 2” included 376 participants matched by age, sex and plasma p-tau217 levels (Simoa) - this was the only analysis where the cognitive status was not considered in the model.
- “Match 3” included 290 participants matched by age, sex and clinicopathological groups (meaning, a combination of cognitive and amyloid pathology status).

Therefore, “Match 1” and “Match 3” are balanced in the proportion of participants with cognitive impairment. As for “Match 2” we have included a Table to provide further information on the distribution of individuals per clinical status in both cohorts.

For transparency and clarity, we have included the Tables in “Supplementary Table 2” for the 3 “Matches”, both for VALIANT and TRIAD cohort.

“*The demographic and biomarker information for the matched data (“Match 1”, “Match 2” and “Match 3”) are presented in **Supplementary Table 2.***”

Match 1

TRIAD cohort			
	**CU (N=192)**	**MCI (N=91)**	**Dem (N=32)**
**Sex (Male)**	63 (32.8%)	39 (42.9%)	26 (81.3%)
**Age**	69 (± 9.7)	71 (± 7.3)	75 (± 6.3)
**Years of education**	15 (± 3.4)	16 (± 3.7)	15 (± 5.0)
**Aß positive**	37 (19.3%)	47 (51.6%)	23 (71.9%)
**p-tau217 Simoa**	0.40 (± 0.45)	0.64 (± 0.55)	1.3 (± 0.94)

VALIANT cohort			
	**CU (N=192)**	**MCI (N=91)**	**Dem (N=32)**
**Sex (Male)**	90 (46.9%)	15 (16.5%)	8 (25.0%)
**Age**	67 (± 10)	73 (± 8.8)	77 (± 11)
**Years of education**	6.8 (± 4.8)	2.3 (± 3.3)	1.9 (± 3.7)
**Aß positive**	26 (13.5%)	19 (20.9%)	11 (34.4%)
**p-tau217 Simoa**	0.25 (± 0.14)	0.30 (± 0.24)	0.34 (± 0.27)

Match 2

TRIAD cohort			
	**CU (N=192)**	**MCI (N=91)**	**Dem (N=93)**
**Sex (Male)**	63 (32.8%)	39 (42.9%)	43 (46.2%)
**Age**	69 (± 9.7)	71 (± 7.3)	67 (± 9.2)
**Years of education**	15 (± 3.4)	16 (± 3.7)	14 (± 4.0)
**Aß positive**	37 (19.3%)	47 (51.6%)	58 (62.4%)
**p-tau217 Simoa**	0.40 (± 0.45)	0.64 (± 0.55)	1.1 (± 0.86)

VALIANT cohort			
	**CU (N=305)**	**MCI (N=51)**	**Dem (N=20)**
**Sex (Male)**	125 (41.0%)	14 (27.5%)	7 (35.0%)
**Age**	67 (± 11)	75 (± 9.1)	79 (± 10)
**Years of education**	5.8 (± 4.6)	2.3 (± 3.3)	2.4 (± 4.2)
**Aß positive**	62 (20.3%)	19 (37.3%)	10 (50.0%)
**p-tau217 Simoa**	0.28 (± 0.17)	0.38 (± 0.29)	0.42 (± 0.32)

Match 3

TRIAD cohort			
	**CU (N=192)**	**MCI (N=66)**	**Dem (N=32)**
**Sex (Male)**	129 (67.2%)	27 (40.9%)	9 (28.1%)
**Age**	69 (± 9.7)	71 (± 8.1)	73 (± 6.7)
**Years of education**	15 (± 3.4)	16 (± 3.6)	14 (± 4.2)
**Aß positive**	37 (19.3%)	22 (33.3%)	11 (34.4%)
**p-tau217 Simoa**	0.40 (± 0.45)	0.56 (± 0.56)	0.87 (± 0.89)

VALIANT cohort			
	**CU (N=192)**	**MCI (N=66)**	**Dem (N=32)**
**Sex (Male)**	101 (52.6%)	50 (75.8%)	24 (75.0%)
**Age**	67 (± 9.9)	72 (± 10)	77 (± 11)
**Years of education**	6.7 (± 4.8)	2.7 (± 3.7)	1.9 (± 3.7)
**Aß positive**	37 (19.3%)	22 (33.3%)	11 (34.4%)
**p-tau217 Simoa**	0.26 (± 0.15)	0.34 (± 0.27)	0.34 (± 0.27)

REVIEWER COMMENTS

We would like to start by expressing our gratitude to the reviewers and editors who devoted their time to provide meaningful contributions to our work. We have given our best trying to address all the suggestions below.

Reviewer #1 (Remarks to the Author):

This is a revised study by Pola et al on the NULISA plasma proteomic findings in the VALIANT cohort. The authors have addressed some of the previous critiques, including now providing more legible figures and tables, although the supplementary tables are not available for review. On re-review of the manuscript, there remain a number of points that should be addressed:

1. Tables for the matches are now provided. Match 1 does not seem to be well matched by sex. Match 2 does not seem to be well matched by sex or pTau217 levels. Match 3 does not seem to be well matched by sex or pTau217 levels. If the goal is to compare to a non-African cohort while controlling for these variables, then ideally one would want to better match the variables. There do not seem to be any statistics presented to describe the match. It is recognized that it may not be possible to exactly match participants on these variables between the two cohorts, but if so, then this should be discussed as a caveat and some statistics provided to demonstrate where the matches have variables that differ between the cohorts.

We thank the reviewer for this important comment. Matching was performed using nearest neighbour propensity score matching implemented with the R package "*MatchIt*", as described in the Methods section. We acknowledge that exact matching across all variables is not always achievable, particularly when comparing cohorts with differing underlying characteristics. As has been presented in manuscript, VALIANT has a large predominance of females and individuals without cognitive impairment, while in TRIAD these differences in proportions are much less evident. While nearest neighbour matching aims to optimize similarity between groups while retaining an adequate sample size, some residual imbalance may remain, unfortunately. It is important to note, however, that in addition to "pairing" the cohorts, we have also included age, sex and groups in the statistical models, still trying to account for these differences.

We agree that this is a limitation that we could not entirely circumvent despite our attempts. Therefore, we have added the text below to the discussion, acknowledging residual differences between cohorts as a study limitation and a possible caveat when interpreting comparisons with the non-African cohort.

"These demographic and clinical differences highlight important distinctions between the present cohort and the predominantly North American cohorts commonly represented in AD research, such as TRIAD. To attenuate these differences and improve comparability while retaining an adequate sample size, neighbour propensity score matching was applied to pair participants across c3ohorts prior to statistical analyses. Nevertheless, exact matching between cohorts was

not fully achievable, and despite adjustment for relevant covariates in the models, this limitation should be acknowledged when interpreting the findings."

2. It is recognized that the same platform is helpful to isolate cohort rather than assay effects when comparing to other populations. However, robustness of the assay itself is also a matter for replication. To this end, it would increase robustness of the findings if the authors could compare their observations to those of Pichet Binette et al Nat Neurosci 2024, for those proteins measured in common between NULISA and the Olink 3k platform employed in this study. A Venn diagram showing the overlap of proteins positively and negatively associated with amyloid, and which proteins are common or uniquely positively and negatively associated with amyloid in the VALIANT cohort, would be helpful. This is a cohort from a high-income Caucasian population, and the underlying platform technologies are similar but not identical.

We thank the reviewer for this thoughtful suggestion, and we will gladly address this point.

When comparing the protein panels of the two methods/platforms, only 21 proteins overlapped for direct comparison (namely: ARSA, CALB2, CNTN2, CST3, CX3CL1, ENO2, FABP3, FCN2, FOLR1, GOT1, ICAM1, IGF1R, IGFBP7, IL6R, KLK6, MAPT, MSLN, NRGN, TIMP3, VCAM1, VEGFD, VGF).

To address this question, we selected the proteins that remained significant after FDR correction in the VALIANT cohort from the primary analysis and subsequently compared the direction of effect across cohorts, irrespective of statistical significance in the replication datasets.

The differential expression analysis results were summarized in a Venn diagram, which showed that several proteins displayed consistent associations across all three datasets, in both the amyloid-positive and amyloid-negative groups. Overall, the VALIANT and TRIAD cohorts demonstrated directional trends that were generally concordant with those reported by Pichet Binette et al.

However, several important considerations should be noted when interpreting these comparisons. First, the findings reported by Pichet Binette et al. were derived from CSF measurements, whereas our analyses were conducted in plasma. As such, direct comparisons should be interpreted cautiously, as observed differences may reflect matrix-specific biological processes rather than cohort-related effects. In addition, the characteristics of the groups compared in Pichet Binette et al. differ somewhat from those included in our analyses. Finally, and perhaps most importantly, protein quantification was performed using two distinct analytical platforms, each method relying on different antibodies and buffers. It is generally unknown at this point whether the assays included in each multiplex panel even capture the same protein fragments or reflect identical biological features. Consequently, discrepancies between studies may arise from technical differences rather than true biological or cohort-specific variation.

For completeness and for the reviewer's reference, we have included this exploratory comparison below. However, we believe that including these analyses in the main manuscript

could lead to confusion and potential misinterpretation, and we therefore chose not to include them in the final version.

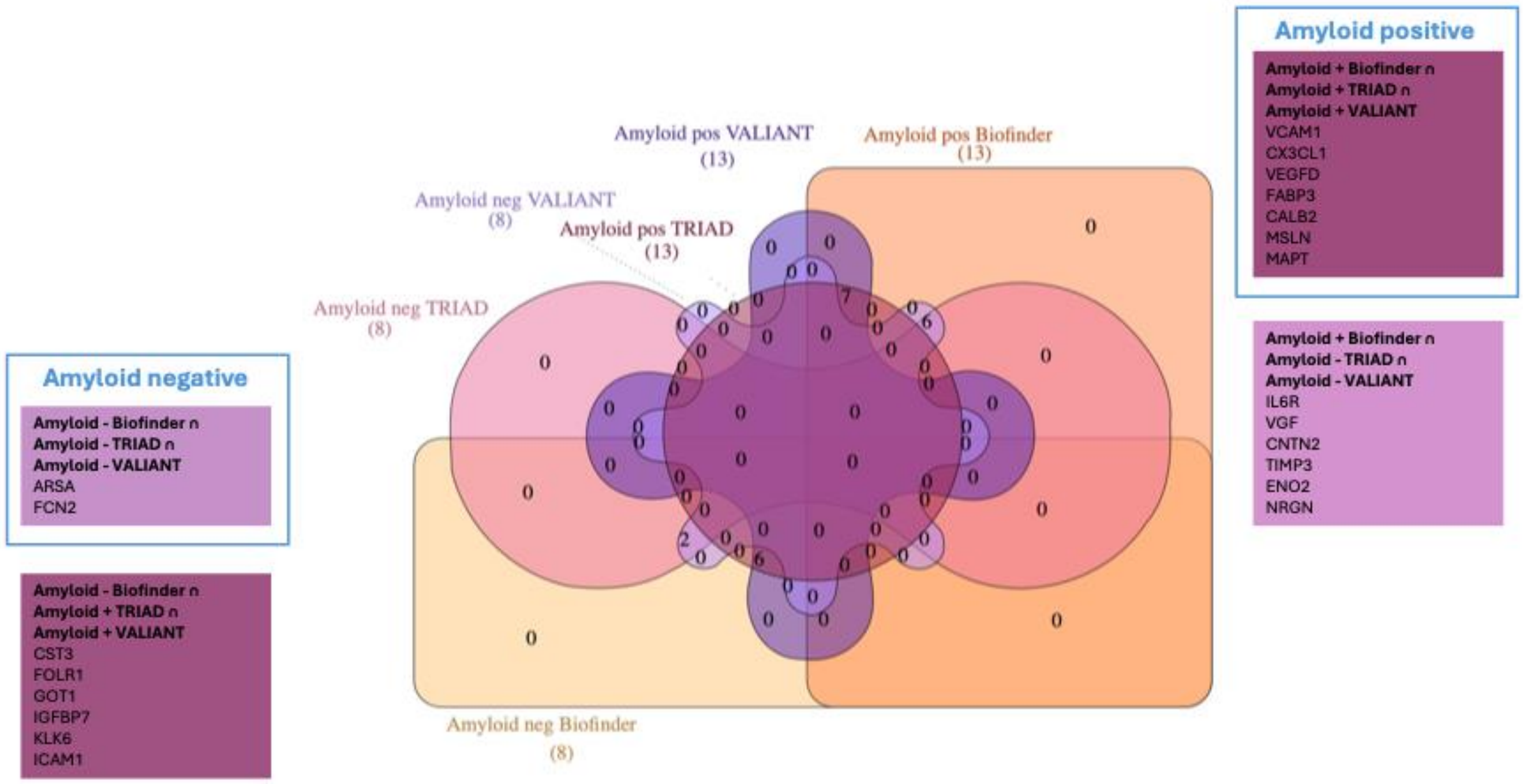

3. In figures 2-4 panels B and C, it should be specified that the analysis is restricted to proteins only positively associated with the outcome (amyloid positivity/negativity, or cognitive impairment/unimpaired).

We thank the reviewer for this comment, and we agree that the legend could benefit from this detail. We have included in the manuscript as follows:

Figure 2B and C:

"The analysis is restricted to proteins identified as significantly differentially expressed between amyloid-positive and amyloid-negative individuals."

Figure 3B and C:

"The analysis is restricted to proteins identified as significantly differentially expressed between CU and CI in amyloid-positive individuals."

Figure 4B and C:

"The analysis is restricted to proteins identified as significantly differentially expressed between CU and CI in amyloid-negative individuals."

4. There should be discussion in the manuscript related to the unexpected finding observed with pTau217 levels compared to other pTau species.

We thank the reviewer for pointing this out, we have included in the manuscript as follows:

"Interestingly, although all plasma p-tau markers were significantly associated with amyloid pathology status, p-tau181 and p-tau231 showed larger effect sizes than p-tau217. A similar pattern was observed in the Tanzanian cohort. However, since amyloid PET data were unavailable for these cohorts, and amyloid pathology status was proxied using plasma p-tau217, direct comparisons will require further investigations."

5. In the legend for figures 2A, 3A, and 4A, if there are no darker points indicating FDR significance in the plots, then the legend should not describe darker points.

We thank the reviewer for this comment. Figures 2A and Figure 4A both includes include nominal and FDR results, therefore remained unchanged. We have updated the legend for Figure 3A accordingly as follows:

"The proteins coloured in light red represent the proteins upregulated in CI participants, while the light purple represent the proteins upregulated in CU participants (nominally significant, P <0.05). ~~The proteins coloured in darker shades represent the proteins with P <0.05 after FDR correction.~~"

6. The legend in 5B-E is confusing to read. Would recommend reworking with punctuation for clarity.

We thank the reviewer for this comment, and we agree that the legend could benefit from this detail. We have updated in the manuscript as follows:

"(**B-E**) Biomarker trajectories using a local weighted regression method (Loess curve). Changes in plasma biomarker levels are represented as Z-scores (anchored to the CU-). **(B)** Loess represents changes of the biomarkers in amyloid-positive participants. **(C)** Loess represents changes of the biomarkers in amyloid-positive participants, stratified by sex. Solid lines represent male participants, while dashed lines represent female participants. **(D)** Loess represents changes of the biomarkers in amyloid-negative participants **(E)** Loess represents changes of the biomarkers in amyloid-negative participants, stratified by sex. Solid lines represent male participants, while dashed lines represent female participants."

7. In the legend for Figure 6, there needs to be a brief description of the goal of each match.

We thank the reviewer for this comment, we have updated as follows, to better describe what each of the "Match" represents. This is both highlighted in the figure and in the legend.

“Figure 6. (A) Venn diagram summarizing the comparison of significant proteins derived from the comparison between amyloid-positive and amyloid-negative individuals in VALIANT (blue shades) and TRIAD (purple shades) cohorts for “Match 1”. Cohorts were matched by age, sex and clinical status (cognitively unimpaired (CU), MCI, Dementia). **(B)** Venn diagram summarizing the comparison of significant proteins derived from the comparison between amyloid-positive and amyloid-negative individuals in VALIANT (blue shades) and TRIAD (purple shades) cohorts for “Match 2”. Cohorts were matched by age, sex and plasma p-tau217 levels (Simoa). **(C)** Venn diagram summarizing the comparison of significant proteins derived from the comparison between amyloid-positive and amyloid-negative individuals in VALIANT (blue shades) and TRIAD (purple shades) cohorts for “Match 3”. Cohorts were matched by age, sex and clinicopathological groups.”
